# Large-scale discovery of protein interactions at residue resolution using co-evolution calculated from genomic sequences

**DOI:** 10.1038/s41467-021-21636-z

**Published:** 2021-03-02

**Authors:** Anna G. Green, Hadeer Elhabashy, Kelly P. Brock, Rohan Maddamsetti, Oliver Kohlbacher, Debora S. Marks

**Affiliations:** 1 Department of Systems Biology, Harvard Medical School, Boston, MA 02115 USA; 2 Biomolecular Interactions, Max Planck Institute for Developmental Biology, 72076 Tübingen, Germany; 3 Institute for Bioinformatics and Medical Informatics, University of Tübingen, Sand 14, 72076 Tübingen, Germany; 4 Department of Computer Science, University of Tübingen, WSI/ZBIT, Sand 14, 72076 Tübingen, Germany; 5 Quantitative Biology Center, University of Tübingen, Auf der Morgenstelle 8, 72076 Tübingen, Germany; 6 Institute for Translational Bioinformatics, University Hospital Tübingen, Sand 14, 72076 Tübingen, Germany; 7 Broad Institute of Harvard and MIT, Cambridge, MA 02142 USA

**Keywords:** Protein sequence analyses, Protein function predictions, Protein structure predictions, Structural biology

## Abstract

Increasing numbers of protein interactions have been identified in high-throughput experiments, but only a small proportion have solved structures. Recently, sequence coevolution-based approaches have led to a breakthrough in predicting monomer protein structures and protein interaction interfaces. Here, we address the challenges of large-scale interaction prediction at residue resolution with a fast alignment concatenation method and a probabilistic score for the interaction of residues. Importantly, this method (EVcomplex2) is able to assess the likelihood of a protein interaction, as we show here applied to large-scale experimental datasets where the pairwise interactions are unknown. We predict 504 interactions de novo in the *E. coli* membrane proteome, including 243 that are newly discovered. While EVcomplex2 does not require available structures, coevolving residue pairs can be used to produce structural models of protein interactions, as done here for membrane complexes including the Flagellar Hook-Filament Junction and the Tol/Pal complex.

## Abstract (web summary)

Our understanding of the residue-level details of protein interactions remains incomplete. Here, the authors show sequence coevolution can be used to infer interacting proteins with residue-level details, including predicting 467 interactions de novo in the *Escherichia coli* cell envelope proteome.

## Introduction

A longstanding goal of molecular biology is to determine the three-dimensional structure of protein interactions at atomic resolution. However, despite the ‘resolution revolution’ in cryo-electron microscopy^1^, atomic resolution of complexes is still labor intensive, lagging far behind the number of known protein interactions in any one organism^2–4^. For instance, even in the well-studied proteome of *Escherichia coli*, less than half of probable protein interactions have been identified^4^ and less than 9% have been structurally characterized, even accounting for similarity to 3D structures of complexes in related species. Hence there is continued interest in computational approaches that can accelerate the discovery of protein interactions in 3D at atomic resolution.

There have been many experimental^5–7^ and computational methods^8–10^ to identify which proteins interact within an organism to scale, but the only computational methods able to determine both interactions and their precise, residue-resolution interfaces are based on coevolution. Coevolutionary methods such as EVcouplings^11,12^ and others^13^ have been successful in determining 3D structures by leveraging the vast corpus of natural sequences using probabilistic graphical models to infer candidate pairs of interacting residues. These coupled positions can be sufficient to fold single proteins^11–13^ and RNAs^14^, without the use of homologous structures, and even resolve protein interaction interfaces de novo^15,16^. However, in the previous work that resolved interacting residues between proteins, coevolution was typically benchmarked on fewer than 100 examples and limited by using co-operonic proteins or phylogeny to identify sequence pairs. Therefore, de novo prediction was limited to select, small datasets or individual runs on a webserver^15–18^. While this work was in preparation, new work was published that used proteome-scale coevolution to provide a dataset of 804 candidate interactions in *E. coli*^19^. Though these predictions are valuable, the methodology is not available for queries of new protein pairs or new large-scale predictions (see Results for detailed comparison).

**Fig. 1:**
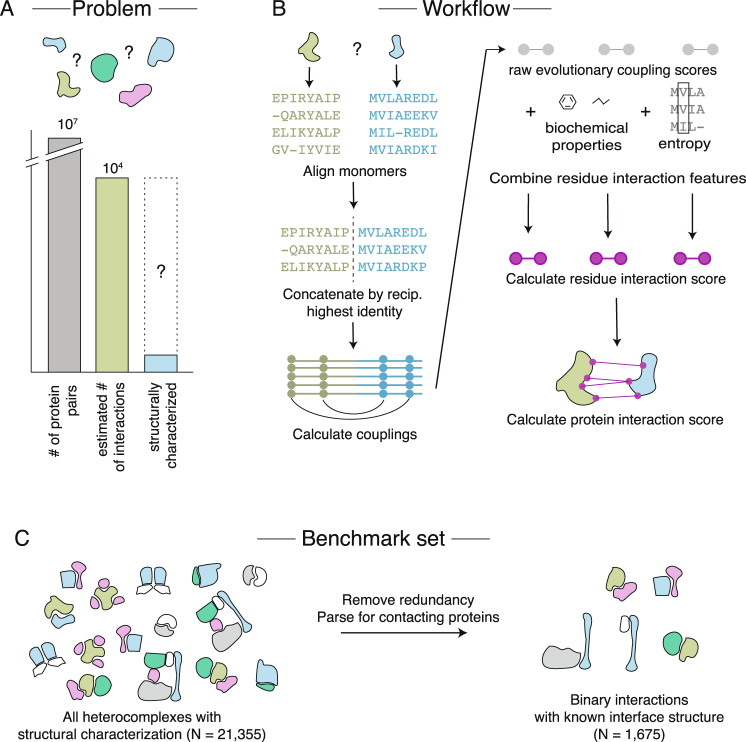
Scaling EVcouplings methods to full bacterial genomes. **A** The search problem for binary protein-protein interactions in *Escherichia coli* involves finding all of the estimated 10^4^ true interactions out of 10^7^ possible pairs. Only approximately 9% of these true direct interactions have a crystal structure solved in *E. coli* or a homologous structure in another organism. **B** Evolutionary couplings learned directly from protein sequences can resolve interfaces. Sequence alignments of both monomeric proteins are created and concatenated by the reciprocal highest identity procedure before inference of evolutionary couplings. Raw evolutionary coupling scores can be combined with features of their distribution, biochemical properties, and sequence entropy to improve inference. **C** A benchmark dataset of all non-redundant protein interactions with known interface structure.

Here we address the challenges of concatenation and statistical scoring to provide a new method that integrates information from residue-level scores to determine whether two proteins interact and the residues involved in their interaction (Fig. 1) without reliance on co-operonic positions, phylogeny, or docking. We estimate that the scope is 53% of all possible protein pairs for a bacterial genome such as *E. coli*, compared to previous methods that rely on genomic position (~10%). As proof of principle, we present results on a new benchmark set, which allows us to assess the specificity and sensitivity of the method. As a case study, we predict 504 interactions involving cell envelope and membrane proteins, which are some of the most challenging to determine experimentally. We provide docked models of these complexes, some of which are of particular biological interest and are discussed in more depth (the Tol/Pal systems and the Flagellar Hook-Filament Junction). We also provide predicted protein interactions at residue resolution for an AP/MS dataset where the precise details of interactions are unknown, as well as residue-level predictions for a Yeast Two-Hybrid dataset. To demonstrate the potential for eukaryotic complexes, we also show successful predictions for eukaryotic-exclusive complexes including the human spliceosome.

## Results

### Most protein interactions in *E. coli* do not have structural resolution

Most protein interactions within an organism remain structurally unresolved, even for well-studied model organisms such as *E. coli*. Of the estimated 10,000 protein interactions in *E. coli*, approximately 3,946 have been observed^4^, involving about 50% of all *E. coli* proteins. Of these protein-protein interactions, 9% have an experimentally determined structure or can be inferred trivially from solved homologous interactions^19^, hence leaving a large fraction of interactions unknown or without structural resolution (Fig. 1).

The majority of the protein monomers in the *E. coli* proteome (3,189 out of a total of 4,391) have high-quality monomer alignments and are therefore amenable to EVcomplex2 (Methods). We verify that these alignments are of high quality by testing the precision of the top ECs for those monomers with an experimental structure (either in *E. coli* or another organism), finding that 78% have reasonable precision of the top ECs (60% for the top *L* ECs, where *L* is the protein sequence length) (Supplementary Data 1, Supplementary Fig. 1). We therefore restrict our computational predictions of co-evolution to the set of 3,189 monomers with high-quality alignments (i.e., 75% of proteins, covering 53% of possible interaction space).

### Probabilistic score allows flexibility for computing residue resolution interactions

This work addresses two main challenges for determining residue resolution of interactions. The first challenge is to avoid the use of genome location or operonic structure and hence be positioned to investigate any protein pair for interacting residues. To solve this, we constructed alignments of pairs of proteins from different organisms by identifying a single protein per species with reciprocal highest identity to the query sequence (Methods). This increases the number of protein interactions that can be tested in *E. coli* by approximately fivefold across the proteome when compared to reliance on genome distance and opens the door to genome-scale interactions in eukaryotic organisms (Supplementary Note 1). This scope and improved coverage will continue to increase as sequencing becomes even more commonplace, as this is a major remaining limitation.

The second challenge is calibrating a method that can both predict residue contacts for known interactions and also use predicted residue contacts to detect protein interactions with high confidence. True interactions are extremely sparse, representing a small fraction of a very large number of possible combination of proteins; for instance, it has been estimated that only about 10,000 of the nine million protein pairs in *E. coli* will interact^4^ (Fig. 1). Therefore, even small false positive rates will result in large numbers of predicted interactions that are false. To minimize the number of false positive interactions, we calibrate on both positive and negative benchmarks: a ‘gold standard’ non-redundant set of 561 interactions with known structure, and a set of 3,987 non-redundant “non-interacting” protein pairs with no signal for interaction in prior high throughput experiments^2,4^ (Supplementary Data 2, Methods).

On these benchmarks, our previous state-of-art method, the EVcomplex score threshold^15^, has a 13% false positive rate, resulting in large numbers of incorrect pairs predicted to interact. These and other methods^15,16,18^ may still be useful if the interaction is already presumed known, but the false positive rate is prohibitively high for unbiased proteome-scale screens. We therefore built a semi-supervised learning method combining the unsupervised EVcomplex score with structure-agnostic features of coevolving residues in a logistic regression: the EVcomplex score, sum of intra-protein EC coupling constraints, the rank of inter-EC relative to intra-EC pairs, and sequence conservation (Supplementary Data 3, Methods). This model achieves a recall of 20.7% at a false positive rate of 0.1% on the held-out test set and outperforms the previous EVcomplex score across all false positive rate thresholds. For cases where the three-dimensional structure of both monomers, is known, the recall can be further increased to 22.6% at a false-positive rate of 0.1% (Supplementary Fig. 3A). We provide full tables of the performance of these scores across all false positive rates, so that users can select thresholds appropriate for their application (Supplementary Data 4). Our model performs comparably to Yeast Two-Hybrid experiments, achieving a recall of 27.9% versus the 29% obtained experimentally at a false positive rate of 1%^4^, with the added feature of providing residue-level interaction for all protein pairs found.

**Fig. 2:**
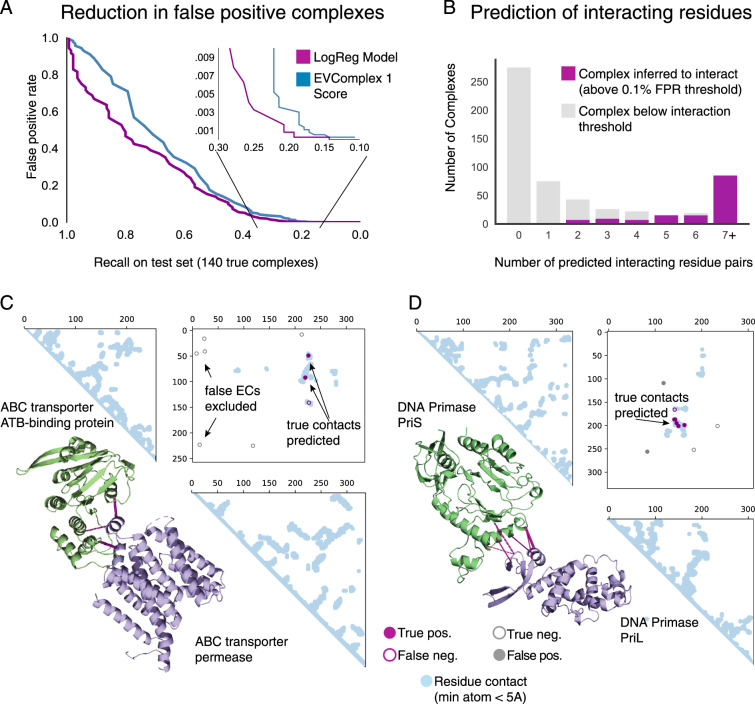
Model predicts interacting residues and interacting proteins with high precision. **A** Recall on the held-out fraction of the positive benchmark set (x-axis) and false positive rate on the held-out dataset of non-interacting complexes (y-axis) at a score threshold that gives the corresponding recall. Our logistic regression model (purple) reduces the false positive rate compared to the previous EV Complex score (blue). **B** Prediction of protein interaction is based on the prediction of interacting residues. Number of predicted interacting residue pairs for complexes inferred to interact (purple) or not inferred to interact (gray) based on our stringent protein complex prediction threshold. **C** Example performance on known interaction between ABC transporter permease and ATP binding subunit (UniProt IDs: Y1470_HAEIN and Y1471_HAEIN, PDB ID: 2NQ2 chains C and A [https://www.rcsb.org/structure/2NQ2]. **D** Example performance on known interaction between DNA primase PriS and PriL (UniProt IDs: PRIS_SULSO and PRIL_SULSO, PDB ID: 1ZT2 chain A and B [https://www.rcsb.org/structure/1ZT2]).

The logistic regression model also outperforms the previous scoring method at detecting residue contacts when the interaction of the protein pairs is presumed known. Considering only the protein pairs in the positive benchmark set, at a score threshold that gives a precision of 80% of true inter-protein ECs, we recover 59.9% of the true ECs in our held-out test set using our previous scoring method, 65.6% of true ECs with the logistic regression model, and 69.3% with the logistic regression model when incorporating features of known monomer structures (Fig. 2, Supplementary Fig. 3). Both our previous EVcomplex score and our new models outperform other previously used scoring methods (raw EC score^19^, Z-score^20^, and raw EVcomplex score^15^) at both predicting interacting proteins and identifying interface residue contacts (Supplementary Fig. 3).

Recently, another computational method using co-evolution to search for proteome scale interactions at residue resolution was published^19^. Based on their reported false-positive rates on their benchmark set, our method is less sensitive for determining the interaction of proteins (our method: 25% recall at FPR 0.34% (Supplementary Data 5), their method: 36% recall at FPR 0.34%). However, this was not a head-to-head comparison and their increased recall may be due to the fact that they did not filter their positive benchmark set for redundancy. In a head-to-head comparison, our method performed better for predicting inter-protein contacts given a known interaction (58.1% vs 75.3% precision across all residues for their reported interacting complexes with known structure, Methods, Supplementary Data 5). Our increased precision comes at some expense in terms of recall, in some cases because accurate inter-protein ECs fall below the chosen scoring threshold, and in other cases possibly because numerous paralogs rendered our concatenation method inaccurate (4% and 18% of the dataset, respectively) (Supplementary Note 1, Supplementary Fig. 4). These comparisons should be considered provisional as their pipeline^19^ is not yet available, so we were not able to test their method on our benchmark dataset, nor were we able to adjust their scoring threshold. An additional advantage of our model is that we provide distinct scoring methods for when interactions are known and unknown. We anticipate this will be important for users who have candidate interactions, e.g., from mass spectrometry experiments.

### New predicted membrane protein interactions with strong co-evolution

**Fig. 3:**
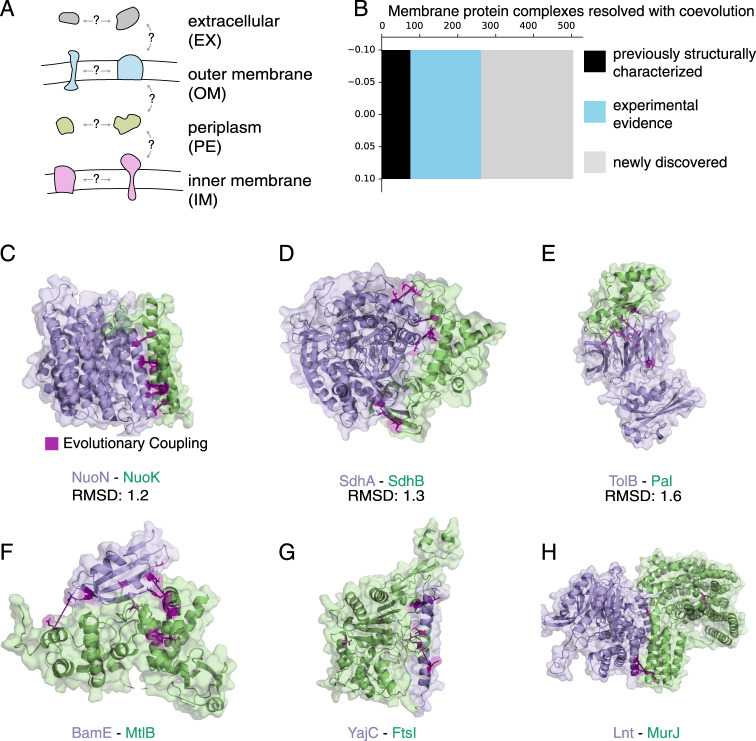
Discovery of hundreds of new interactions in the *E. coli* membrane proteome. **A** We searched a high-value subset of the 10^∧^7 possible interactions in the *E. coli* proteome by searching membrane compartments with themselves and with adjacent membrane compartments. **B** We found 504 high-scoring protein interactions in the cell envelope, including 75 with structural characterization and 186 with previous experimental evidence (and no structural characterization). **C**–**E** 3D configurations of previously structurally characterized interactions are accurately predicted by molecular docking with inferred restraints. RMSDs calculated by comparison to known structures with PDB IDs 3RKO [https://www.rcsb.org/structure/3RKO], 2WU2 [https://www.rcsb.org/structure/2WU2], and 2HQS [https://www.rcsb.org/structure/2HQS], respectively. **F**–**H** Example of three docked models of newly resolved protein complexes: BamE/MltB, YajC/FtsI, and Lnt/MurJ. Evolutionarily coupled residues used as restraints in docking are shown in magenta and connected with solid lines.

Since the cell membrane contains many protein interactions essential for life, but is notoriously difficult to study experimentally^21^, we targeted the cell envelope proteome for detailed analysis. We based our analysis on 1583 proteins previously described as localized to the *E. coli* cell envelope^2^. We assayed each compartment of the *E. coli* cell envelope proteome with itself and with adjacent compartments (Methods) (Fig. 3A), for a total of 939,159 protein pairs. After monomer sequence alignment, concatenation, and EC calculation, 198,534 protein pairs comprised of 1053 proteins (566 non-redundant protein families) pass quality thresholds for analysis. The majority (771) of these proteins are inner membrane proteins. 49% of these proteins have at least a partial structure known of themselves or a homologous protein.

We predict 504 interactions in the *E. coli* cell envelope as well as the details of their interacting residues, including 243 interacting pairs not previously observed experimentally (Fig. 3B, Supplementary Data 7, Supplementary Fig. 3). Despite having chosen a stringent false positive rate threshold of 0.1%, we expect that up to 39.1% of these interactions may be false positives due to the large size of the tested space. Of 305 known membrane protein interactions with known structures that can be analyzed by our method, we recover an interaction signal for 76 of them (Supplementary Fig. 5), similar to the recall expected based on our calibrations (24.9% here vs 20.7% in calibration). The protein interactions in the *E. coli* membrane with the highest interaction score are involved in essential cellular processes; for instance, the RodA and penicillin-binding protein cell wall polymerases, which we previously characterized in a detailed study^22^, as well as components of the electron transport chain, components of the ATP synthase, and components of the bacterial flagellum.

Although AP/MS has recently been used to identify 12,807 co-purifying membrane proteins, it is not always known which proteins interact with which other proteins within their respective complexes^2^. We therefore computed the probability score of the pairs within each complex to determine interacting pairs, finding significant residue-level coevolution for 872 pairs (three or more ECs above the 80% precision residue interaction threshold), 262 of which would have been blindly identified using our stringent protein interaction scoring threshold (Supplementary Data 7). These predictions can be used to disambiguate directly interacting proteins in complexes identified via AP/MS, such as the Tol-Pal complex reported below.

Yeast Two-Hybrid experiments have provided a rich dataset of candidate interactions in *E. coli*^4^. EVcomplex2 can both increase confidence in the inferred interactions and also provide residue resolution for the predicted complexes. For 21% (*N* = 559) of complexes found to interact by Yeast Two-Hybrid, we find at least one EC above the 80% precision residue interaction threshold (and 198 complexes have three or more ECs) (Supplementary Data 7). This does not necessarily mean that complexes with no residue coevolution signal do not interact, as lack of signal may be due to chosen threshold in our benchmarking or to lack of evolutionary conservation of complexes.

### Docking of newly resolved membrane-associated interactions

The inter-protein residue pairs identified by our method can be used as restraints for molecular docking, to resolve the 3D structure of protein complexes^15,16^ (Fig. 3C–E). In addition, docking lends additional confidence to ECs below the residue interaction scoring threshold: ECs below the threshold end up satisfied in the final model for 58% of the docked benchmark complexes (Supplement, Supplementary Fig. 6, Supplementary Data 8, Supplementary Data 10).

Of all inferred membrane protein interactions, we docked 59 pairs that had structures of both monomers available, including 36 with no previous structure of their interaction (Supplementary Data 9, Supplementary Data 11). This includes the proteins BamE and MltB (Fig. 3F), which had previously been determined to be in the same complex in an AP/MS experiment but the details of their interaction were unknown^2^. We determine an interaction and resolve the interface for the peptidoglycan biosynthesis protein FtsI and the subunit of the Sec translocation complex YajC (Fig. 3G), as well as for Lnt and MurJ (Fig. 3H), involved in lipoprotein maturation and peptidoglycan biosynthesis, respectively.

**Fig. 4:**
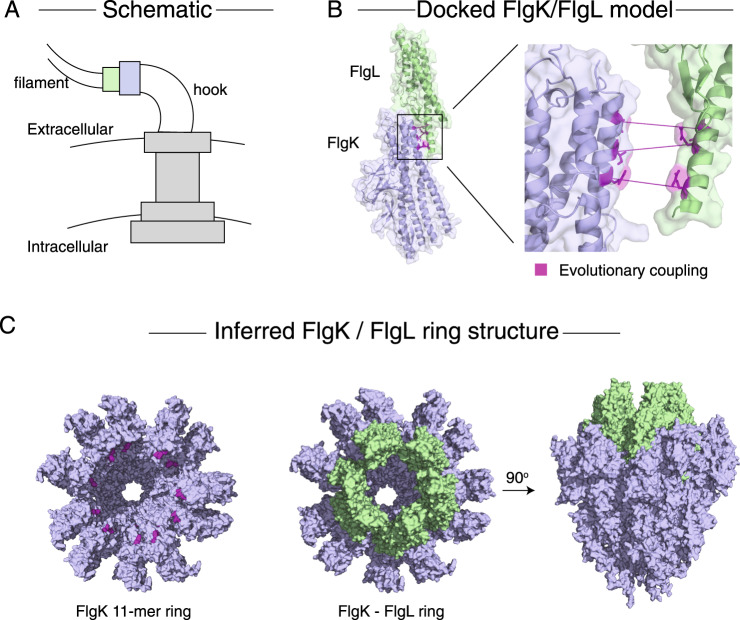
Model of the flagellar hook-filament junction. **A** Schematic of the orientation of the bacterial flagellum. The proteins FlgL (green) and FlgK (blue) form two rings which create the junction between the hook and filament of the flagellum^24^. **B** Docked model of FlgL and FlgK using evolutionary couplings. PDB structures of homologous proteins from *Salmonella typhimurium* were used in docking (PDB IDs 2D4Y [https://www.rcsb.org/structure/2D4Y], and 2D4X [https://www.rcsb.org/structure/2D4X], respectively). Predicted interface residues are highlighted in purple. **C** A previously inferred model of the FlgK ring from *Campylobacter jejuni*^24^ was used to infer the structure of the entire hook-filament junction. Evolutionary coupled residues (purple) show the interface for FlgL ring insertion into the FlgK ring. By aligning our docked model to the *C. jejuni* ring, we show that an 11-mer ring of FlgL fits inside the FlgK structure.

Our method resolves the interaction interface between flagellar proteins FlgL and FlgK, which form the junction between the flagellar filament and flagellar hook^23^. Extrapolating from our docked model of the monomers and a previous model of the FlgK ring from *Campylobacter jejuni*^24^, we infer the configuration of an 11-mer ring of FlgL inside an 11-mer ring of FlgK^24^ (Fig. 4, Supplementary Fig. 7).

**Fig. 5:**
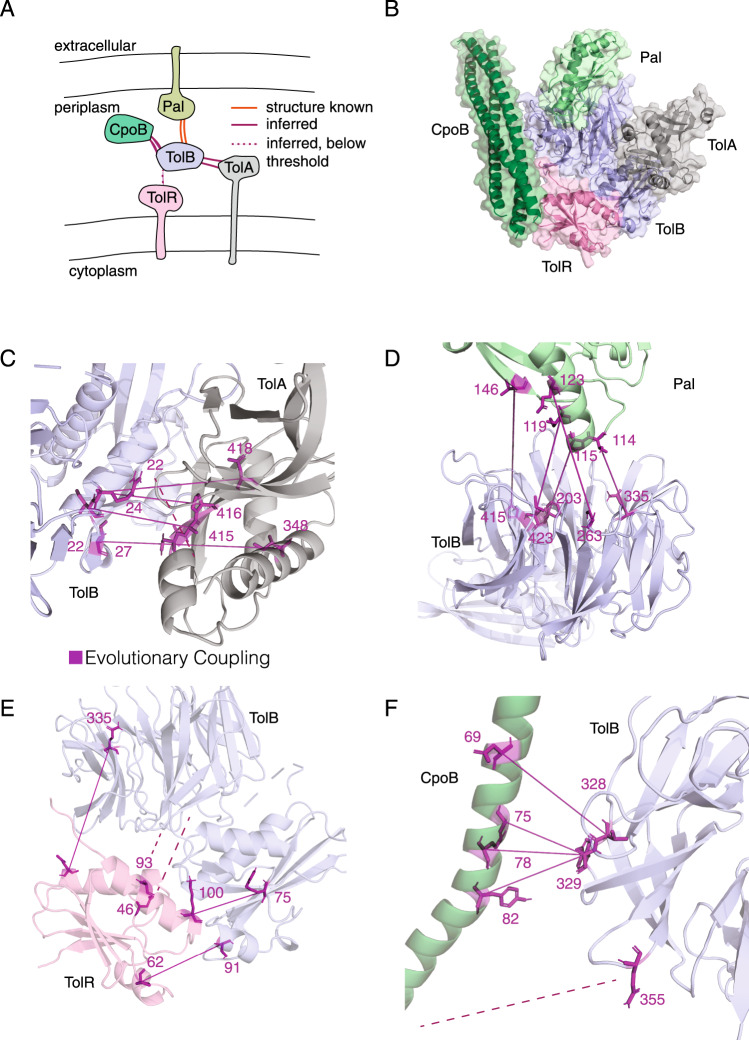
Atomic resolution model of the Tol-Pal complex. **A** Schematic of the proteins involved in the Tol-Pal complex^2^ (TolABR, CpoB, and Pal). Interactions with previously solved interfaces are shown in orange and interactions inferred by our method are shown in purple. **B** Complete model of the Tol-Pal complex inferred by aligning results of docked pairwise models. Note that CpoB is inferred to be a trimer in vivo but was docked as a monomer for modeling purposes **C**–**F** Residue resolution of TolB-Pal, TolB-TolA, TolR-TolB, and CpoB-TolB interfaces. The top 5 inferred interface contacts are shown in purple. Dashed lines indicate inferred contacts where one or more residues are missing from the solved structure. Structures used are 1TOL_A (TolA) [https://www.rcsb.org/structure/1TOL], 2HQS_A (TolB) [https://www.rcsb.org/structure/2HQS], 5BY4_A (TolR) [https://www.rcsb.org/structure/5BY4], 2HQS_H (Pal) [https://www.rcsb.org/structure/2HQS], and 2XDJ_A (CpoB) [https://www.rcsb.org/structure/2XDJ].

We constructed an atomic model of the Tol/Pal system (Fig. 5), a molecular machine spanning the inner and outer membranes of Gram-negative bacteria. These proteins may play a role in membrane constriction during bacterial cell division^25^. For the known interacting interface between TolB and Pal^26^, we accurately recapitulate the known structure (PDB ID: 2HQS [https://www.rcsb.org/structure/2HQS]). We predict interaction and residue contacts for the known interaction between TolA and TolB^4,27^ as well as residue contacts and a likely interaction of TolR and TolB in the membrane^28^. For CpoB, which interacts with the Tol system and the cell division septum site^29^, we provide a molecular model of its interaction with TolB.

### Current scope of EVcomplex for interactions exclusive to Eukaryotes

**Fig. 6:**
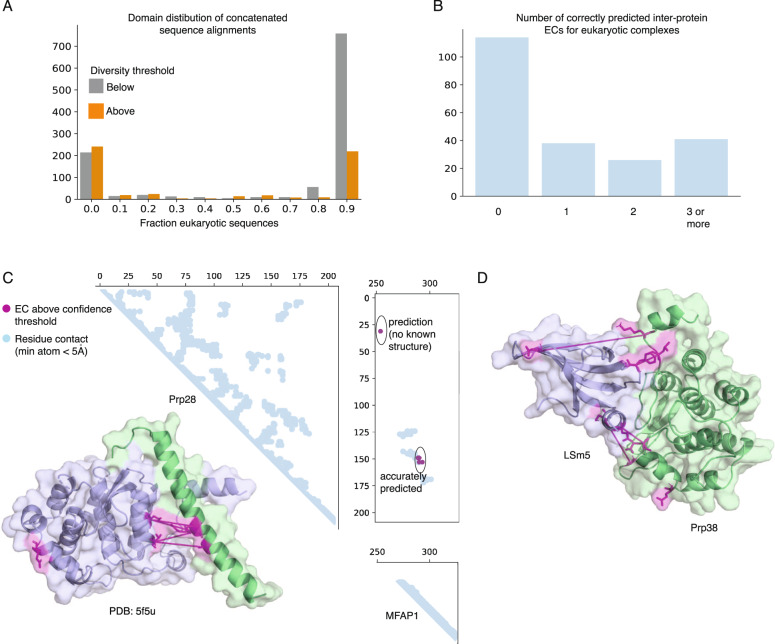
Predicted protein interactions in eukaryotic proteomes. **A** Distribution of eukaryotic sequences in concatenated sequence alignments. Shown in orange are alignments that passed our sequence diversity threshold, and in gray are those that did not. **B** Number of correctly predicted inter-protein ECs for eukaryotic-exclusive complexes above the sequence diversity threshold. Eukaryotic-exclusive complexes are defined as complexes whose concatenated sequence alignment is at least 90% eukaryotic sequences. Inter protein ECs are defined as correct if their minimum atom distance is <8 Å. **C** The human spliceosome proteins Prp38 and MFAP1 have a known interface correctly predicted by our method, and have a protein interaction detected. The inter-protein ECs above the 80% precision threshold used throughout the paper are shown in purple. Known protein structure (PDB ID: 5F5U, [https://www.rcsb.org/structure/5F5U]) is used to visualize the subunits. **D** Schematic of predicted interaction between Lsm5 and Prp38 with top 5 inter-protein ECs.

The prediction of protein interactions using coevolution is not restricted to bacterial genomes. However, predicting protein interactions using coevolutionary methods in eukaryotes is difficult due to the smaller number of sequenced genomes and higher numbers of paralogous genes compared to bacteria. We used our positive benchmark set, which contains structures from all domains of life, to assess the prospects of EVcomplex2 for predicting protein interactions at residue resolution in eukaryotes (Methods). Of the 1,675 protein pairs in our dataset, 977 are found predominantly in eukaryotes (more than 90% of concatenated sequences are eukaryotic in origin) (Fig. 6). As expected, a higher fraction of the eukaryotic protein complexes were excluded from downstream analyses due to low sequence diversity compared to bacterial protein complexes (78% versus 53% excluded, respectively). Approximately 11% of the 977 eukaryotic protein interactions have at least one interacting residue pair correctly predicted by EVcomplex2, and 4% have three or more correctly predicted (Fig. 6).

An important eukaryotic complex is the spliceosome, an RNA–protein complex which splices intronic sequences from immature mRNA in eukaryotes^30^. EVcomplex2 correctly predicts the interface between Prp38 and MFAP1, whose interactions contribute to the activation of the spliceosome^31^ (Fig. 6). Excluding alignments with low sequence diversity, the next two highest-ranked interactions in the spliceosome are between LSm2 and MFAP1 as well as between Prp38 and LSm5, which could plausibly come into contact during rearrangement of the spliceosome during activation. These results demonstrate the possibility of using sequence coevolution-based methods for eukaryotes.

We assessed the current prospects for predicting whether proteins interact and their residue resolution on the 74,449 proteins in the human proteome, by aligning their constituent PFAM domains^32^ (Methods). We find that 42% of proteins in the human proteome can be aligned with medium sequence diversity in at least one domain, and 20% of proteins can be aligned with the high diversity cutoff used for *E. coli* in all of their domains. These represent approximate upper and lower bounds on the number of proteins that would be amenable to coupling-based analysis with any concatenation method. Of the 3 × 10^9^ possible interactions in the human proteome, this means that between 4% and 17% of protein interactions are currently accessible to EVcomplex2.

## Discussion

In this work, we demonstrate a method for inferring protein interactions at residue-level detail from genomic sequences alone. We provide a probabilistic scoring method, calibrated on large, non-redundant datasets, which can be used to determine the probability of interaction of two proteins. If the protein interaction is known or inferred by the protein interaction score, the residue interaction score can be used directly to determine residues in contact at the interface. These methods can be freely accessed via our website (https://marks.hms.harvard.edu/ecolicomplex/) or by downloading the source code (https://github.com/debbiemarkslab/EVcouplings)^33^.

The highest reciprocal identity concatenation method introduced here applies to more complexes than concatenation methods relying on genomic distance to correctly pair interacting paralogs^15^, and perform comparably to a recent method that relies on reciprocal best hits for entire bacterial genomes^19^. Our method only requires the sequence annotation already present in the UniProt database and therefore has the advantage of not requiring curation and all-vs-all sequence searches of full genomes^19^, phylogeny construction^18^, or extensive iterations^34,35^, rendering it very fast with no dependencies. We show that cases where our concatenation method is outperformed tend to have larger numbers of paralogs, suggesting that other more computationally intensive techniques could be selected based on prior knowledge about the protein pair.

Our method assumes that interactions between orthologous proteins are conserved across species, an assertion which is certainly not true for all complexes and will lead to false negatives in the case of newly evolved or poorly conserved interactions. In addition, since our method has been calibrated on experimentally resolved structures, it may inherit their biases. In particular, crystal structures are known to be biased against membrane proteins, alternative conformal states, and transient interactions, which could lead to false-negative predictions. This bias is likely to be more pronounced in eukaryotes, which tend to have more multi-domain and intrinsically disordered proteins than prokaryotes^36^.

With current sequence databases, 53% of the protein pairs in the *E. coli* proteome are eligible for EVcomplex2. By using our highest reciprocal identity criterion, we restrict ourselves to one ortholog per genome, which limits the amount of sequence diversity that can be found for families with multiple paralogs per genome. This limitation is most consequential in eukaryotes, where there are fewer sequenced genomes to draw from. Previous studies showed that eukaryotic protein complexes could be successfully predicted if they had prokaryotic homologs^20,37^, and our study extends this finding to show that some eukaryotic-exclusive complexes now have sufficient sequence diversity for prediction. Growth in sequence databases and the development of fast concatenation techniques to handle paralogous sequences will increase the number of protein pairs that can be analyzed. Two recent papers have shown that sequence diversity generated in the lab can be used to determine protein structures^38,39^, opening an interesting future avenue for using experiments to determine the structure of protein complexes.

Method development using coevolution to resolve protein-protein interactions has thus far been tested on a handful of well-studied protein-protein interactions^15–17,34,35,40^, reaffirming our ability to predict well-conserved, well-characterized protein interactions. While there are many possible avenues for continuing to improve coevolution-based prediction of protein interactions, we suggest that future development should begin with large, non-redundant biological datasets such as ours. By identifying and diagnosing cases where existing methods fail on real datasets, new developments will lead to greater opportunities to discover unknown protein interactions.

## Methods

### Creation of positive benchmark set

We built a dataset of non-redundant heterodimeric protein-protein interactions with resolved crystal structures. A list of all heteromeric structures in the PDBePISA was downloaded^41^ (download date: 2/19/2018) and passed through the RCSB Protein Data Bank with the following query: “Sequence Length is between 30 and 1200 and Resolution is between 1.0 and 5.0 and Representative Structures at 30% Sequence Identity.” IDs were mapped to UniProt^42^ identifiers using the SIFTS database^43^ (download date 2/20/2018). Only complexes with at least two chains that map to different UniProt identifiers were kept. Single-chain and fusion proteins were removed because they may have non-specific interface contacts not found in nature.

For each protein, we extracted the PFAM domains^32^ annotated in that protein, which we call the PFAM set for that protein. We then consider a protein-protein interaction as unique if and only if the interacting proteins constitute a pair of PFAM sets not yet seen in our database, yielding 4154 non-redundant pairs of interactions. We then consider only contacting pairs of proteins, defined as a protein pair with at least 20 pairs of residues with a minimum atom distance less than five angstroms. Because each unique interaction can be represented by many crystal structures, representative crystals were chosen based on the number of other interfaces present in that crystal – i.e., complexes with more subunits were prioritized. We removed any cases where the two proteins map to the same chain, where the two proteins share any PFAM domain, or where either of the proteins was less than 30 amino acids in length, resulting in a final list of 1,675 protein complexes with known interfaces (Supplementary Data 2).

### Creation of negative benchmark set

A total of 2,500 negative examples were selected randomly from all pairs of complexes with no signal for interaction in a large-scale Yeast Two-Hybrid experiment in *E. coli*^4^, as well as all pairs of proteins whose proteins were found in different APMS benchmark complexes^1^. We verified that no pair of proteins had the same PFAM set as any other pair of proteins in the negative set and that no pair had the same PFAM set as any pair of proteins in the positive benchmark set. We further removed protein pairs with any links in the STRING database^44^, protein pairs previously inferred to interact (including protein-ribosome pairs)^19^, and proteins from membrane protein export apparatuses (as annotated in Babu et al., 2017): “BamABCDE Outer Membrane Protein Assembly Complex”, “TatABCE protein export complex”, “SecD-SecF-YajC-YidC Secretion Complex/Sec Translocation Complex”, “Sec Translocation Complex/SecYEG translocase”, “Sec Translocation Complex”, “GspC-O secreton complex.” The final dataset included 3987 protein pairs (Supplementary Data 2).

### Monomer sequence alignment

For alignments of the positive benchmark set, all UniProt^42^ identifiers corresponding to the sequence of interest were extracted from PDB^45^. For alignments of the *E. coli* genome, all identifiers from the reviewed *Escherichia coli* strain K12 reference proteome (UP000000625) were downloaded from UniProt. For both the positive benchmark set and *E. coli* genome set, the entire length of every protein was used for sequence alignment. Alignments were constructed using jackhmmer version 3.1b2^46^ with five iterations against the UniProt database (downloaded February 2018), as implemented in the EVcouplings software package. Alignments were considered to have sufficient coverage if at least 80% of the columns were less than 50% gaps. A range of bit-scores were tested (0.1, 0.2, 0.3, 0.5, and 0.8 times protein length *L*). For each alignment, the N_eff_ was calculated by downweighing each sequence by the number of other sequences with more than 80% identity. The alignment with the largest N_eff_ that had 80% coverage of columns was selected for each protein. Proteins from the *E. coli* genome were deemed eligible for EVcomplex analysis if their monomer sequence alignment had at least 80% column coverage and N_eff_/L > = 2.5, after characterizing the precision of monomer protein couplings across a range of sequence alignment diversities (Supplementary Fig. 1).

### Concatenation

Monomer protein sequences were concatenated for EVcomplex2 analysis. We first annotate the species of origin for each sequence by extracting the species annotation from the UniProt^42^ database. A single representative for each species was chosen based on the highest identity to the query sequence. After excluding closely related paralogs in the query genome with more than 90% sequence identity, we confirm that the candidate hit has the highest identity to the query sequence than to other paralogs from the query species found during our sequence alignment protocol. If for a single species, a reciprocal highest identity hit for both monomers can be identified, those hits are concatenated and added to the alignment.

### Concatenated alignment quality control

To avoid analyzing protein pairs where the two proteins are homologous, we implemented four different quality control metrics. First, we remove all pairs of proteins where the first protein contains a PFAM domain that is found in the second protein. Then, we remove all pairs where our structure comparison protocol found overlapping hits to the same chain of the same PDB structure. Next, we remove all pairs that display high-scoring coevolution along a diagonal between the two proteins (i.e, between position *i* in protein A and the corresponding position *i* in protein B). We consider these contacts to be artifactual due to their very high scores relative to known interactions. Finally, we do not consider proteins from *E. coli* where there is a non-traditional amino acid annotated in the UniProt sequence, because these can indicate pseudogenes. To ensure high-diversity, low gap alignments for inference, we removed from consideration concatenated sequence alignments with N_eff_/L less than 0.2 or coverage of columns less than 80%.

### EC calculation

For calculation of evolutionary couplings (ECs), hits composed of more than 50% gaps were filtered from the alignment, and sequences with homologs more than 80% identical were downweighed to compute N_eff_, the effective number of sequences^12^. ECs were calculated using pseudo-likelihood maximization^14,47,48^. The λ_J_ term was scaled by the number of amino acids minus one times the number of sites in the model minus one^49^. Pre- and post-processing was performed using the EVcouplings Python package^33^.

Raw EC scores, sometimes called the Corrected Norm (CN) score, were calculated by performing the average product correction (APC) on the Frobenius Norm scores output by plmc. Diagonal ECs, where positions *i* and *j* are within 5 amino acids, were not included for further analysis. For analysis of raw ECs, the Z-score of each inter-residue EC was calculated using the distribution of all inter-residue ECs. The EVcomplex score was calculated by dividing each inter-protein EC by the absolute value of the minimum inter-protein EC score (for the raw EVcomplex score), and then normalizing for the N_eff_/L of the concatenated alignment (for the EVcomplex score)^15^. The EVcomplex2 score is calculated by inputting the couplings scores and additional features into the model described under “Detecting inter-protein residue contacts”, for predicting residue interactions when the protein interaction is presumed known, or by inputting the coupling scores and additional features into the model described under “Detecting protein interactions” for predicting protein interactions.

### Comparison of ECs to experimental structures

To identify experimental structures for comparison of evolutionary couplings, the monomer sequence alignments were searched against the PDB database using *hmmsearch* version 3.1b2^46^. For *E. coli* monomer alignments, the top 20 were selected. For the validation dataset, all hits above a bitscore threshold of 0.2 *L* were selected. Once structures were selected for comparison, the minimum of the nearest atom inter-residue distances across all structures were used for comparison to ECs^11^.

### Solvent accessible surface area calculation

Relative and absolute solvent accessibilities were calculated using DSSP (CMBI version by M.L. Hekkelman/2010-10-21)^50^ with a probe size of 1.4 Å and standard van der Waals radii^51^. If multiple structural hits were found for each protein, we take the mean relative solvent accessibility across all structures, to avoid biases caused by incomplete structures.

### Conservation calculation

The conservation *C* for each column *i* in the concatenated sequence alignments were calculated as one minus the normalized entropy for the column *i*,

1 $$C = 1 - H/{\mathrm{log}}_220$$

2 $$H = - \mathop {\sum }\limits_{k = 1}^{20} f_k{\mathrm{log}}_2f_k$$

where *f*_*k*_ is the frequency of the *k*-th amino acid in column *i*.

### Detecting inter-protein residue contacts

We trained a logistic regression classifier to predict the probability that two residues *i* and *j* are in contact (distance < 8 Å), given their EC score and additional residue-level features. This model for residue interaction was trained on the positive benchmark dataset, which includes 560 protein complexes for which the concatenated sequence alignment has sufficient coverage and diversity. Because most of the inter-protein ECs are noise and do not correspond to real contacts^15,16^, we take the top ten inter-protein ECs as input to the model. Residue pairs where one or both residues were missing in all structures were removed from the dataset because their distance is unknown. This resulted in 5,344 residue pairs with a defined minimum atom distance, 26% of which were within 8 Å.

140 (25%) of the 561 complexes had all of their ECs held out and used for testing. Models were trained using the Logistic Regression classifier in Scikit-learn v. 0.23.5^52^, using the LIBLINEAR solver and L1 regularization. Regularization weight of 0.05 was chosen to maximize a tradeoff between recall and precision using ten-fold cross-validation on the training dataset.

The following features were evaluated for their ability to predict residue contacts in a logistic regression model with L1 regularization: the EVcomplex score^15^; the Z-score of the corrected norm (CN) score of all inter-protein ECs; the conservation of the more conserved for the two residues; the relative rank of the inter-protein ECs; the maximum of the intra-protein EC enrichment^11^ for the two residues; the percent of residues in both positions that were hydrophobic (amino acids AVILMC), hydrophilic (amino acids STNQ), bulky (amino acids WFY), or charged (amino acids RHKDE) (4 separate features); and the combined hydrophilicity score at both positions. The features chosen for the final model based on non-zero weights in the L1 regression were the EVcomplex score^12^, the conservation of the more conserved for the two residues, the relative rank of the first inter-protein EC, and the maximum of the intra-protein EC enrichment for the two residues^11^ (Supplementary Data 3). The resulting model is referred to as the “structure-free” score.

We also trained a model that takes advantage of data from known monomer structures by incorporating two additional features: the minimum of the accessible surface area of both residues, and the precision of the ECs ranked above the current inter-protein EC. These two features were found to be significant in combination with the features found above. This model is referred to as the “structure-aware” score.

### Detecting protein interactions

To build a model for determining whether two proteins interact, we use the score of the residue interaction model for the top ten inter-protein residue pairs as inputs to a logistic regression, as well as the relative rank of the highest-scoring inter protein EC (Supplementary Data 3). Our training dataset was composed of positive and negative examples. Positive examples were 420 protein pairs (75%) from the positive benchmark set, filtered so that all examples had at least one of the top ten inter-protein ECs within 8 Å, for a total of 322 complexes. Negative examples were 322 protein complexes randomly chosen from the negative benchmark set of Y2H negative complexes. Our test dataset was composed of the same 140 positive benchmark protein complexes withheld during the training of the residue interaction model, as well as all examples in the negative benchmark set not included during training.

Models were trained using the Logistic Regression classifier in Scikitlearn v. 0.23.5^52^, using the LIBLINEAR solver and L1 regularization (weight = 0.1) to select features and regularization weight, chosen to maximize a tradeoff between recall and precision using ten-fold cross-validation. The final model was trained with L2 regularization using the LIBLINEAR solver in Scikitlearn v. 0.23.5.

The protein pairs from the negative benchmark set that were excluded from the training and test set were used to assess the false-positive rates expected when the model is applied to large datasets.

### Comparison to Cong et al., *Science*, 2019

Recall and false positive rate for prediction of interacting proteins was extracted from Cong et al., *Science*, 2019 Supplementary Table S3^19^. All protein pairs with a crystal structure listed were extracted from their Supplementary Table S8 (“Protein–protein interactions in *E. coli* identified in the co-evolution screen”). 331 of 339 protein pairs were successfully run using our pipeline, with the remaining 8 excluded due to technical issues or pathologies of the protein pair (Supplementary Data 4). Both our and their inter-protein ECs were compared to the same PDB structures as listed in their study, using a minimum atom distance cutoff of 8 Å to define interaction.

Our pipeline was considered to have predicted an inter-protein EC if the structure-free logistic regression score exceeded the 80% precision cutoff. To investigate the causes of discrepancies between prediction methods, we calculated the median number of paralogs per species for each of the constituent monomer alignments.

### Cell envelope proteome

Localizations of *E. coli* proteins were extracted from a previous study^2^. Cell envelope proteins were separated into four categories: inner membrane-bound (integral or lipoprotein), periplasmic, outer membrane-bound (integral or lipoprotein), and extracellular. Proteins annotated as “membrane related” but without a specific location were excluded. Each of the four compartments was tested against itself and against proteins in the physically adjacent compartment(s): inner membrane versus periplasm, periplasm versus outer membrane, and outer membrane versus extracellular.

### Docking

Side chains of all monomers were perturbed and optimized using SCWRL4^53^ to reduce the effect of crystallization in a complex that may bias the docking procedure. Restraints-based docking was done using HADDOCK (High Ambiguity Driven biomolecular DOCKing) v2.2^54^ and CNS^55^. This allows restricting the possible interaction search space using a more sophisticated treatment of conformational flexibility. The top five inter-ECs scores in both structure-aware and structure-free regression models were selected. Unambiguous distance restraints were applied on C-beta (except for glycine, where C-alpha was used) with an effective distance of 5 Å with an upper and lower bound of 2 Å. Default HADDOCK protocol was done starting with a rigid-body energy minimization (500 models), followed by semi-flexible refinement in torsion angle space (100 models), and ending with further refinement in explicit water (100 models). Upon binding, small conformational changes can be accounted for by HADDOCK through explicit flexibility during the molecular dynamics refinement. The resulting models were scored using the default HADDOCK scoring function, and the top ten best-scoring models, as well as the top models from RMSD clusters, are reported. To evaluate docking of our benchmark set where the structure of the interaction is known, we calculated Ligand-RMSD (L-RMSD) as well as Ligand centroid rotation angle and displacement in comparison to the reference structures.

### Annotating domain of life for concatenated alignments

For all concatenated sequence alignments that comprise the positive benchmark set, the UniProt^42^ accession numbers were mapped against the NCBI taxonomy database^56^ We extracted the domain of life for each sequence in the database and calculated the percent of sequences in each alignment that were eukaryotic in origin.

### Generating PFAM domain alignments

For estimating the number of possible human interaction pairs suitable for our analysis, we used a set of EVcouplings runs computed for all ~16 K PFAM domains in 2017 (PFAM release 30.0). A representative sequence from each domain family was chosen, and alignments were built for each sequence using jackhmmer^46^ for five iterations at multiple length-normalized bit scores between 0.1 and 0.5 using the 2017 Uniref100 release^42^. Pseudo likelihood maximization was then used to compute evolutionary couplings scores for each possible pair of positions in the query.

### Estimation of human protein pairs amenable to EVcomplex

We downloaded the set of 74,449 unique translation products annotated by UniProt human reference proteome UP000005640 (dated 12/2/2019). Each entry was then cross-referenced against the PFAM 32.0 release to identify component domains. Using the set of runs described above, we then identified how many domains in each protein had an alignment with high coverage (with over 80% of positions containing fewer than 30% gaps in the alignment) and a sufficiently diverse set of sequences (effective number of sequences per residue > = 1 or 5). These calculations were then used to report the number of human proteins amenable to EVcomplex reported in the text.

### Reporting summary

Further information on research design is available in the Nature Research Reporting Summary linked to this article.

## Supplementary information

Supplementary Information

Description of Additional Supplementary Files

Supplementary Data 1

Supplementary Data 2

Supplementary Data 3

Supplementary Data 4

Supplementary Data 5

Supplementary Data 6

Supplementary Data 7

Supplementary Data 8

Supplementary Data 9

Supplementary Data 10

Supplementary Data 11

Reporting Summary

## Data Availability

The benchmark and prediction datasets generated are available in the Supplementary Material, and full results are available online at https://marks.hms.harvard.edu/ecolicomplex/. The sequences and sequence annotation data analyzed during this study are available from UniProt (download date: Apr 1, 2017). The sequence location data analyzed during this study are available from ENA (download date: Feb 2017). The sequence taxonomy data analyzed during this study are available from the NCBI taxonomy database (download date: May 2020). The 3D-Structural information data analyzed during this study are available from the Protein Data Bank and SIFTS (download date Feb 1, 2018).

## Code availability

EVcomplex2 is available as part of the EVcouplings Python Package, which is available on github under an MIT license: https://github.com/debbiemarkslab/EVcouplings and citable as 10.1093/bioinformatics/bty862.
