# Supplementary material for: Large-scale discovery of protein interactions at residue resolution using co-evolution calculated from genomic sequences
Source: Nat Commun. 2021 Mar 2;12:1396. doi: 10.1038/s41467-021-21636-z (PMC7925567; doi:10.1038/s41467-021-21636-z)
Supplement: Supplementary file 1 — Supplementary Information [file 41467_2021_21636_MOESM1_ESM.pdf]

Supplementary Material for:

Large-scale discovery of protein interactions at residue resolution using co-evolution calculated from genomic sequences

Anna G. Green[1*] and Hadeer Elhabashy[2,3*], Kelly P. Brock[1], Rohan Maddamsetti[1], Oliver Kohlbacher[2,3,4,5,6**], Debora S. Marks[1,7**]

[*] AGG and HE contributed equally to this manuscript. Please cite as Green and Elhabashy *et al.*, or Elhabashy and Green, *et al.*
** Joint corresponding authors (oliver.kohlbacher@uni-tuebingen.de, debbie@hms.harvard.edu)

[1] Department of Systems Biology, Harvard Medical School, Boston, MA 02115, USA
[2] Biomolecular Interactions, Max Planck Institute for Developmental Biology, 72076 Tübingen, Germany
[3] Institute for Bioinformatics and Medical Informatics, University of Tübingen, Sand 14,72076, Tübingen
[4] Department of Computer Science, University of Tübingen, WSI/ZBIT, Sand 14, 72076 Tübingen, Germany
[5] Quantitative Biology Center, University of Tübingen, Auf der Morgenstelle 8, 72076 Tübingen, Germany
[6] Institute for Translational Bioinformatics, University Hospital Tübingen, Sand 14, 72076 Tübingen, Germany
[7] Broad Institute of Harvard and MIT, Cambridge, MA 02142, USA

Supplementary Note 1

Comparison to operon-based sequence concatenation

In initial studies on detecting protein interaction at residue resolution from genome sequences, sequences were concatenated based on distance in their genome of origin, thus limiting the application to proteins found within the same operon in bacteria[1,2]. Our highest reciprocal identity concatenation method was designed to allow many more complexes to be accessible to coevolution-based methods than with our previous operon-based concatenation methods. To compare these two methods, we ran our previously published operon-based concatenation protocol[2] on our true positive benchmark dataset of 561 proteins that achieved sufficient sequence diversity in their concatenated alignment. This previous protocol pairs sequences if and only if they are (1) reciprocally the closest to one another on the genome versus any other paralog and (2) within 10,000 nucleotides. Genome location annotation was extracted

from the ENA database[3] (Download Date: Feb 2017), and protein sequences that could not be mapped to the ENA database were discarded in concatenation.

Of the 561 protein interactions in our positive benchmark dataset, 358 could not be concatenated to sufficient sequence diversity (N_{eff} / L >= 0.2) using operon distance because they are exclusively eukaryotic (**Methods**) or are too infrequently found in the same operon in bacterial genomes. For the remaining 203 complexes, we compared the accuracy of the top 10 ECs found using the two concatenation methods. 26% of protein pairs had the same accuracy with both methods, 39% had higher accuracy with highest reciprocal identity concatenation, and 35% had higher accuracy with the operon distance concatenation.

We investigated the reasons for the differences in performance by comparing the median number of paralogs found per species in the monomer sequence alignments. The complexes where performance is better with the operon-based concatenation method tend to have more paralogs in the monomer sequence alignments, (median of median per-species paralogs = 3) than those where performance is better with the highest reciprocal identity method (median of median per-species paralogs = 2). This indicates that while in general the highest reciprocal identity concatenation renders more complexes eligible and produces comparable or higher accuracy, bacterial complexes that are known to have large numbers of paralogs may be better predicted with an operon-based method.

Comparison of previously published scoring methods for ECs

Many scoring methods for inter-protein evolutionary couplings have been used in the literature, including the raw CN score (the result of applying the Average Product Correction to the Frobenius Norm score)[4], the Z-score of the raw CN scores,[5] and the EVcomplex score[2], a transformation of the CN score using the diversity of the concatenated sequence alignment [2]. Both the Z-score and the EVcomplex score are a monotonic transformation of the CN score. We assessed the performance of these scores at discovering true complexes and true residue contacts on our positive benchmark set of 561 complexes that achieved sufficient sequence diversity for prediction. Because these scoring methods are unsupervised, we used the entire benchmark set for assessment. We find that the corrected EVcomplex score performs best both at recovering true residue contacts, and recovering true complexes in our dataset (**Supplementary Fig. 2)**.

Comparison to Cong et al, *Science,* 2019

We performed a head-to-head comparison of interacting residue prediction by running the positive benchmark set from Cong et al., *Science*, 2019[4] using our pipeline. On their positive benchmark set of 339 complexes, our method reports higher-precision inter-protein ECs (58.1% vs 74.6%, across 220 complexes where we report significant ECs), at the expense of tending to report fewer ECs per complex (**Supplementary Fig. 4A, Supplementary Table 4)**. For the 109 remaining complexes where we report no significant ECs, 25 complexes were reported with a low precision (< 30%) prediction in the prior study as well, 21 complexes had high precision inter-protein ECs from our pipeline that fell below our defined confidence threshold, and 63

complexes were predicted with high precision by Cong *et al*. but not by our method. Those 63 pairs of proteins constitute only 25 non-redundant pairs (based on PFAM domain composition), and tend to have higher numbers of paralogs than complexes which performed well using our method **(Supplementary Fig. 4A)**.

Molecular docking on the positive benchmark set

To assess whether molecular docking is capable of further increasing the accuracy of the predicted inter protein ECs, we performed molecular docking on a subset of our positive benchmark set, for a total of 173 complexes. Our docking procedure tends to produce accurate models, with 54% achieving a centroid ligand-RMSD < 10Å among those where at least three true restraints were used for docking, and 45% among all models **(Supplementary Figure 6)**. The more correct inter-protein ECs are used for docking, the more likely the models are to have low RMSD. Among these 173 protein complexes, we find that docking "rescues" correct ECs for 100 complexes – inter protein ECs that were below the confidence threshold end up in contact in the highest scoring docked model. This indicates that docking is capable of further boosting the signal for accurate inter-protein ECs, even if the top-scoring docked model is not itself accurate.

Supplementary Figure 1: High-quality sequence alignment of protein monomers from *E. coli*. (A) The precision of the top ECs increases as the effective number of sequences (N_{eff}) divided by sequence length (L) increases, with a plateau at approximately $N_{eff}/L = 2.5$. Each point corresponds to a monomeric protein in *E. coli* for which there is a crystal structure or structure of known homolog. The x-axis was truncated at 20 to show detail in the low range. (B) A histogram of the precision of the top L ECs for proteins whose sequence alignments had N_{eff}/L >= 2.5

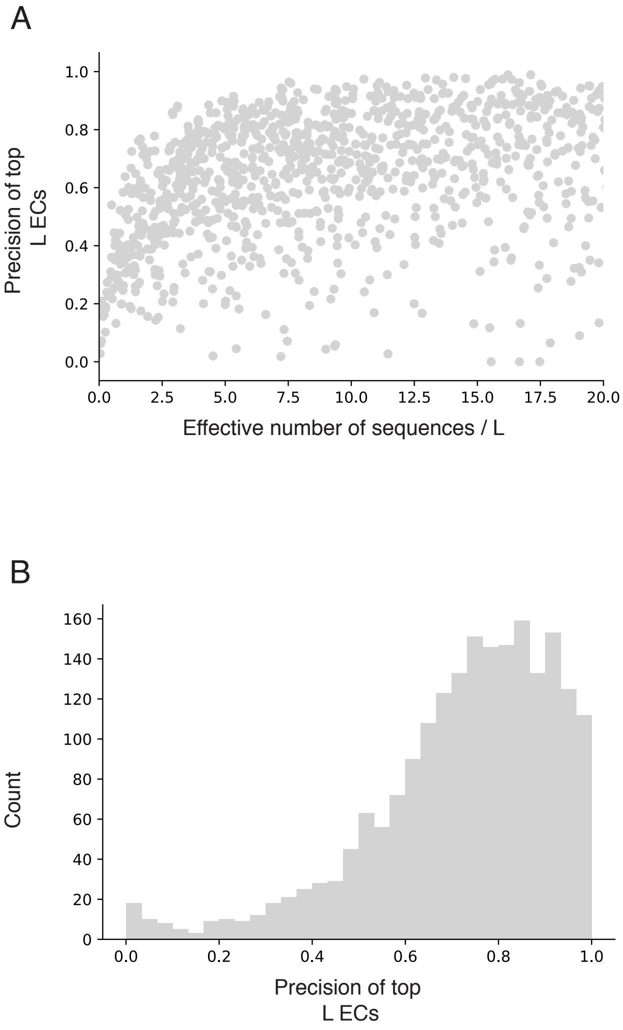

Supplementary Figure 2: Performance of previously published EC scoring methods on the benchmark dataset.

Performance of the corrected norm (CN) score, Z-score of the corrected norm score, and EVcomplex score (both corrected and uncorrected) on the entire benchmark dataset, considering the top 20 ECs for each complex. (A) Precision-recall curve for recovery of true residue contacts. Contacts are considered true if they are within 8Å (B) Precision-recall curve for recovery of true complexes. Complexes are considered 'true' if at least one of the top twenty ECs represents a contacting residue pair. Complexes are considered recovered if at least one EC is above the score threshold.

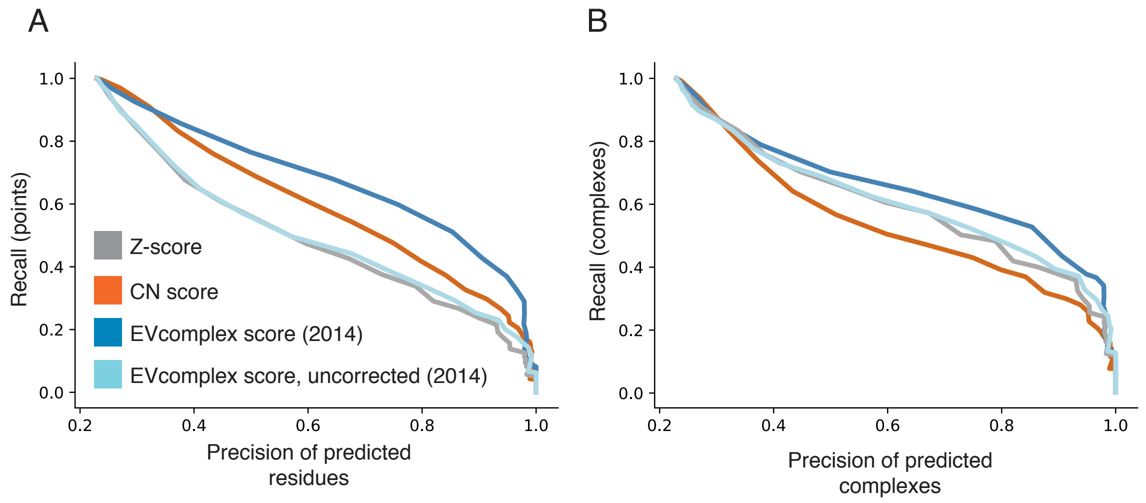

Supplementary Figure 3: Features of monomer protein structure further increase precision and recall on the held-out test set (A) Our logistic regression model (purple) reduces the false positive rate compared to unsupervised assessments of EC scores (grey, blue, and orange). A logistic regression model that also incorporates features of monomeric protein structure (green) further outperforms the structure-agnostic logistic regression (purple). The x-axis shows the recall on the held-out positive benchmark set, and the y-axis shows the false positive rate on the held-out dataset of non-interacting complexes at the score threshold that gives each recall value. (B) Once the interaction of the proteins is known, incorporation of features of monomeric structures increases model recall for a given precision (purple), and precision is further increased when features based on monomer structures are introduced (green). The precision versus recall of the top 10 ECs for protein complexes with known interfaces is plotted. ECs are considered true if inter-residue minimum atom distance is less than 8Å.

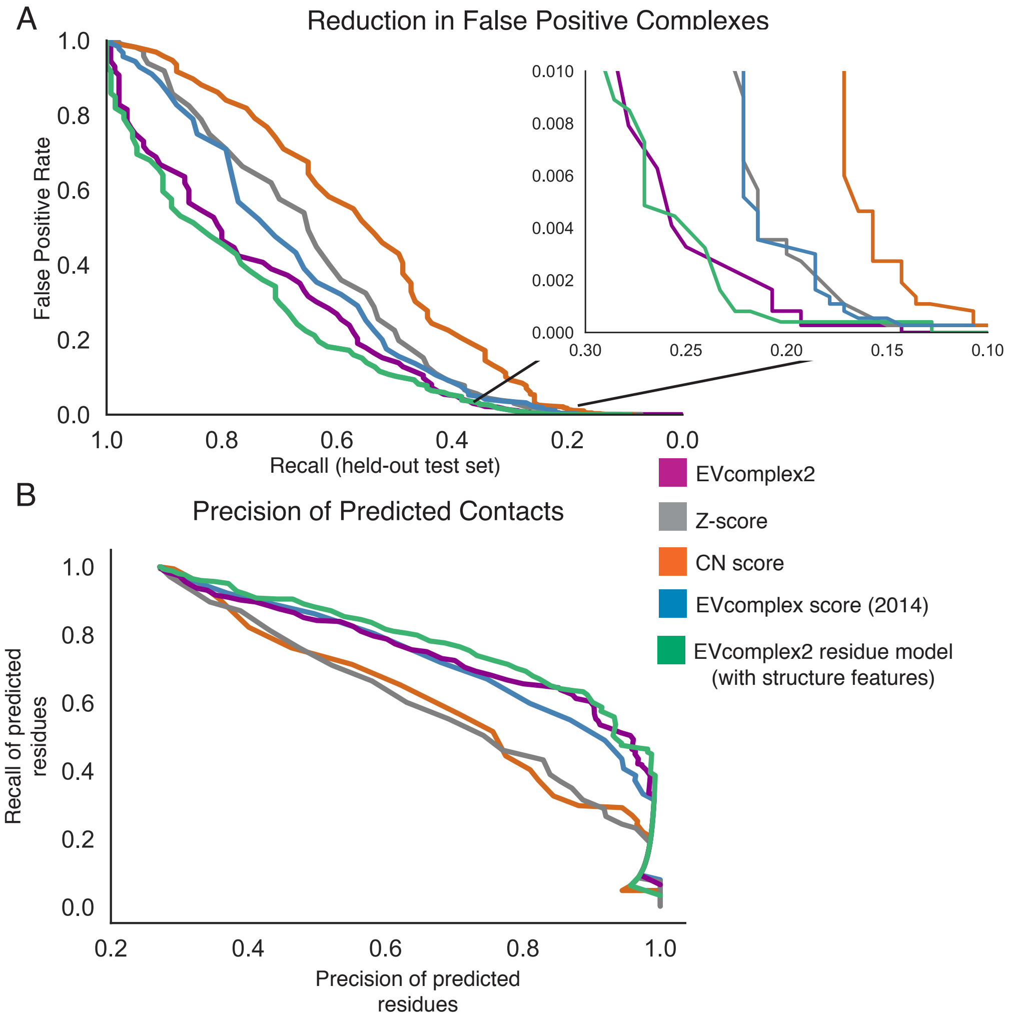

Supplementary Figure 4: Comparison to Cong, et al. Science 2019. (A) Comparison of the precision and recall of our methods to those reported in Cong, *et al.* Science 2019, for 215 complexes from the Cong positive benchmark set for which we report significant inter-protein ECs under our structure-free residue scoring model. Our model tends to report higher precision ECs, at the expense of number of significant residues reported. (B) For those complexes reported with reasonable precision (>= 30%) in Cong *et al.* where our method predicts no significant ECs, we examine the median number of paralogs per species in the sequence alignment, and compare to the 215 with significant ECs using our method. Box plot center line, median; box limits, upper and lower quartiles; whiskers, 1.5x interquartile range; points, outliers.

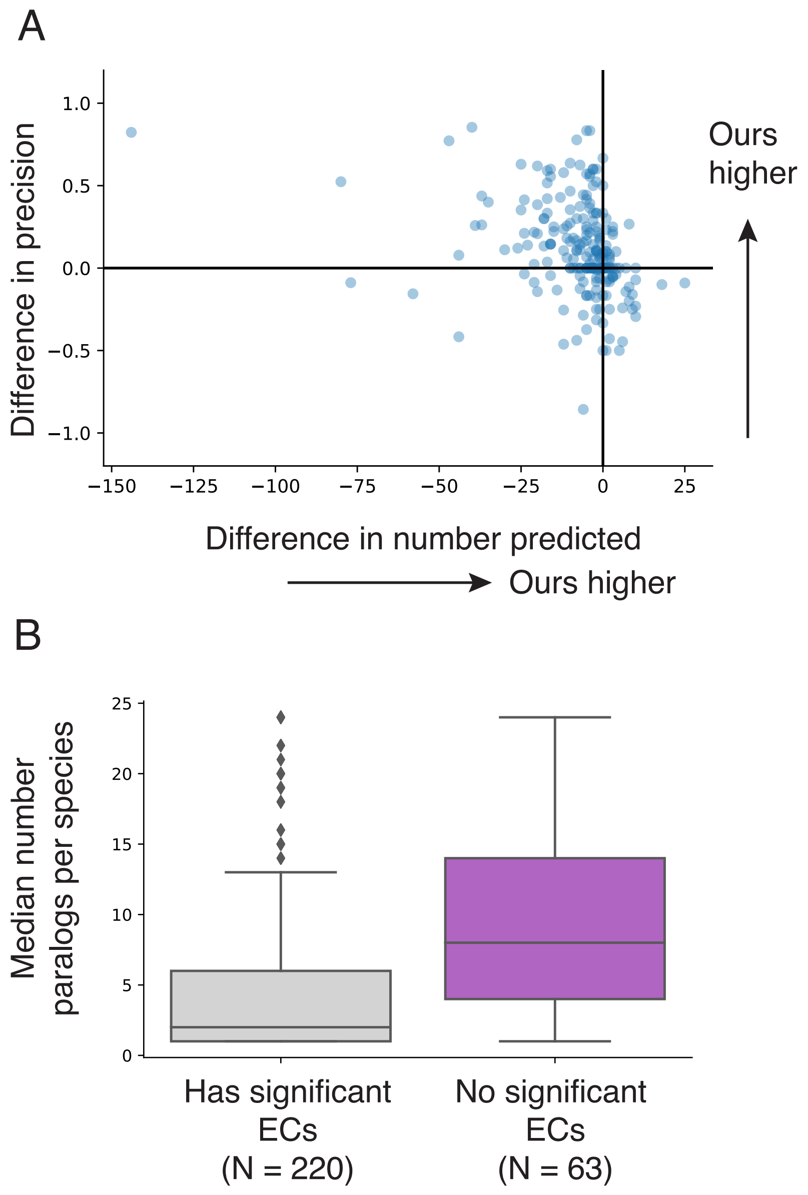

Supplementary Figure 5: Protein interaction scores for cell envelope proteome. Comparison of our new scoring method (Logistic Regression Model without structure-derived features), i.e. "EVcomplex2", to previous scoring method[2]. Orange dots indicate proteins found to interact in APMS experiments[7] with a solved crystal structure of their interaction, blue dots indicate all other proteins. Cutoff for inferring interaction used in this paper is shown as a grey line.

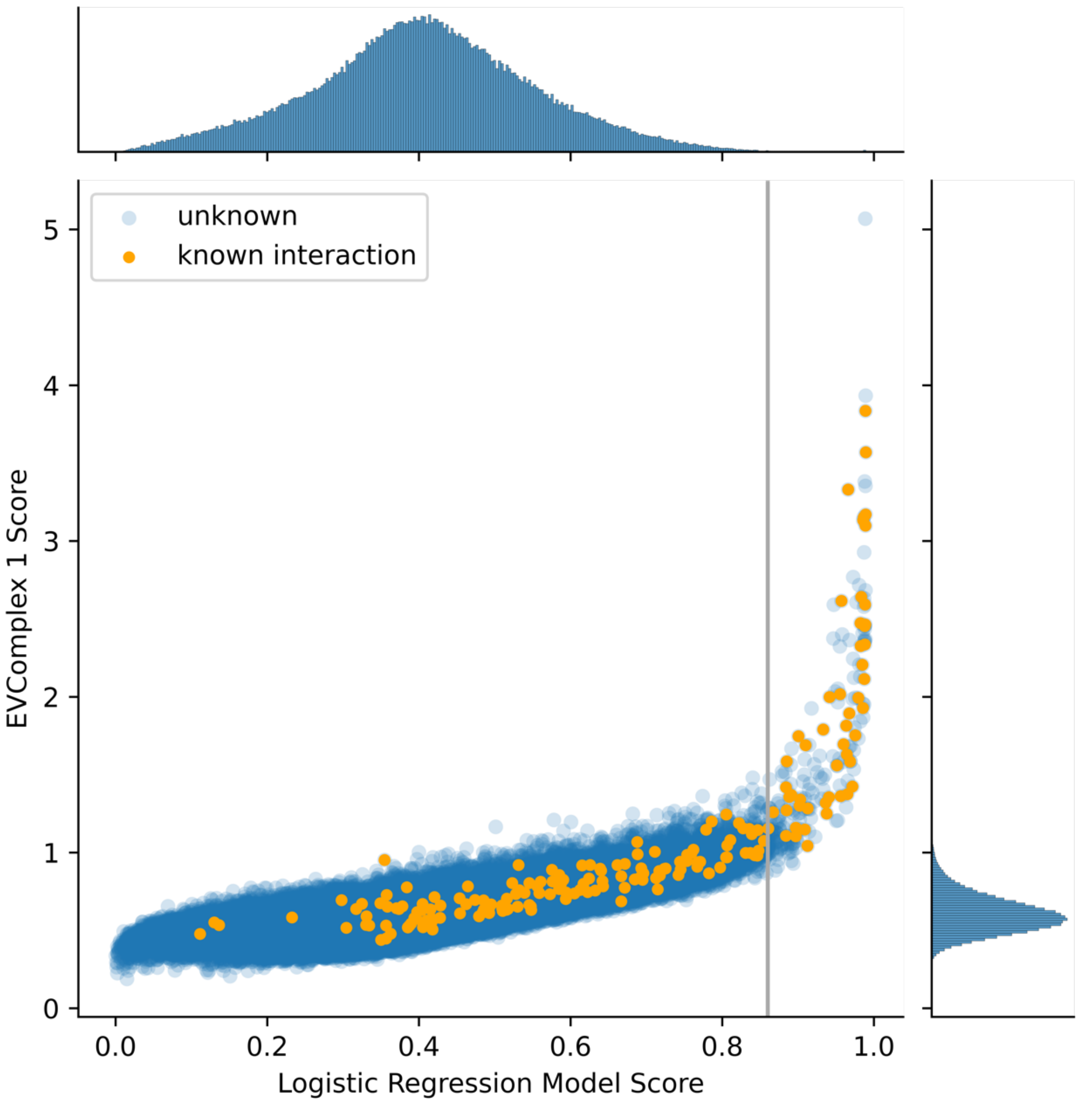

Supplementary Figure 6: Docking performance in the positive benchmark set

(A) RMSD values (Å) for docked models in the positive benchmark set (N=173). The RMSD of the top model, blindly selected based on the HADDOCK scoring function, for each protein complex is shown (B) Effect of the number of residues used for docking on the Centroid Ligand RMSD of the top model. Predictably, CL-RMSD is lower for models generated with more correct ECs (min. atom distance < 8Å). Of note is an outlier (represented here with an 'x'), RpoE/ChrR (PDB ID 2Q1Z [https://www.rcsb.org/structure/2Q1Z][8]), which is unsolvable with rigid-body docking because ChrR penetrates and loops around RpoE when the two are in complex – a major conformational change from the monomer state. (C) Number of true EC's below the structure-aware model residue interaction threshold that are "rescued" (*i.e.* have < 8Å min. atom distance) in the top docked model.

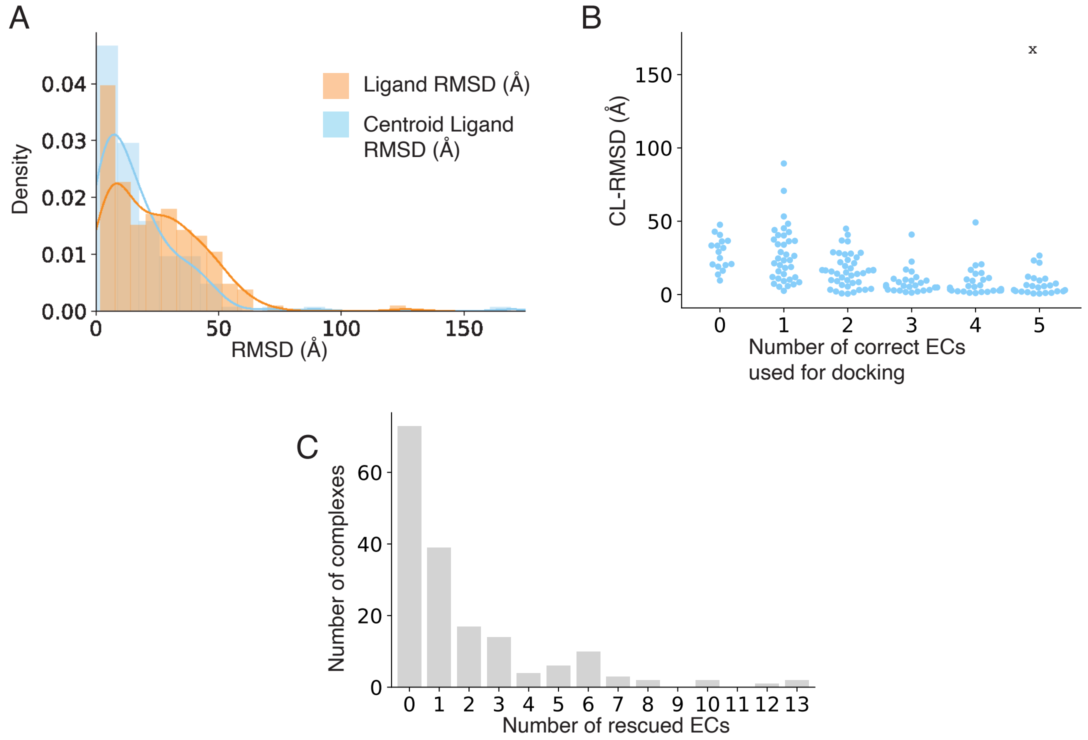

Supplementary Figure 7: Inferred ring structure of flagellar hook-filament junction from three species

Ring structure of FlgK (blue) and FlgL (green) from three different species (A) *Campylobacter jejuni,* (B) *Salmonella typhimurium,* (C) *Escherichia coli*. The *S. typhimurium* ring model was constructed by docking monomer structures 2D4Y [https://www.rcsb.org/structure/2D4Y] and 2D4X [https://www.rcsb.org/structure/2D4X] using evolutionary couplings, and then arranging eleven copies of the lowest energy model in a ring based on the coordinates of the *C. jejuni* FlgK ring model[9]. The *E. coli* ring model was created by making a homology model[10] of the *E. coli* FlgL and FlgK proteins against the monomer structures 2D4X and 2D4Y, performing docking using evolutionary couplings, and then arranging eleven copies of the lowest energy model in a ring based on the coordinates of the *C. jejuni* FlgK ring model. The *C. jejuni* ring was created using the structure 5XBJ [https://www.rcsb.org/structure/5XBJ] of FlgK and a homology model of the *C. jejuni* FlgL created with 2D4X as a template, performing docking using evolutionary couplings, and then arranging eleven copies of the lowest energy model in a ring based on the coordinates of the *C. jejuni* FlgK ring model.

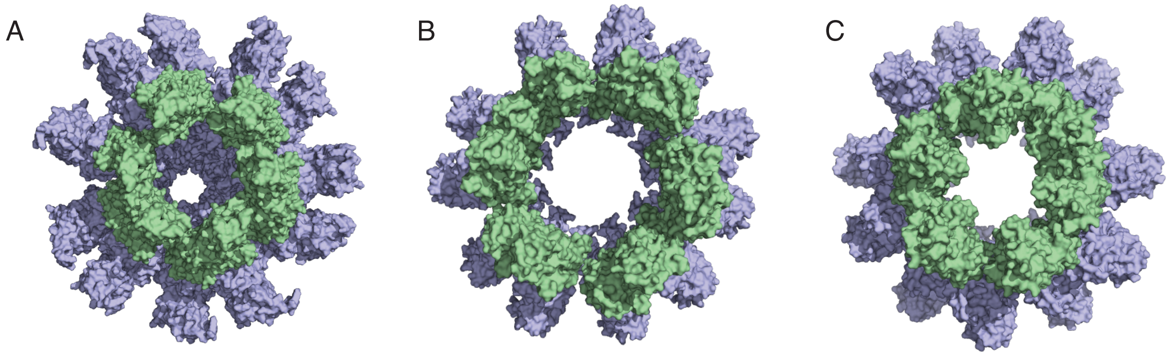

Supplementary References

1 Ovchinnikov, S., Kamisetty, H. & Baker, D. Robust and accurate prediction of residue-residue interactions across protein interfaces using evolutionary information. *Elife* **3**, e02030, doi:10.7554/eLife.02030 (2014).

2 Hopf, T. A. *et al.* Sequence co-evolution gives 3D contacts and structures of protein complexes. *Elife* **3**, doi:10.7554/eLife.03430 (2014).

3 Pakseresht, N. *et al.* Assembly information services in the European Nucleotide Archive. *Nucleic Acids Research* **42**, D38-D43, doi:10.1093/nar/gkt1082 (2013).

4 Cong, Q., Anishchenko, I., Ovchinnikov, S. & Baker, D. Protein interaction networks revealed by proteome coevolution. *Science* **365**, 185-189 (2019).

5 Rodriguez-Rivas, J., Marsili, S., Juan, D. & Valencia, A. Conservation of coevolving protein interfaces bridges prokaryote-eukaryote homologies in the twilight zone. *Proc Natl Acad Sci U S A* **113**, 15018-15023, doi:10.1073/pnas.1611861114 (2016).

6 UniProt Consortium, T. UniProt: the universal protein knowledgebase. *Nucleic Acids Res* **46**, 2699, doi:10.1093/nar/gky092 (2018).

7 Babu, M. *et al.* Global landscape of cell envelope protein complexes in Escherichia coli. *Nature biotechnology* **36**, 103 (2018).

8 Campbell, E. A. *et al.* A conserved structural module regulates transcriptional responses to diverse stress signals in bacteria. *Mol Cell* **27**, 793-805, doi:10.1016/j.molcel.2007.07.009 (2007).

9 Bulieris, P. V., Shaikh, N. H., Freddolino, P. L. & Samatey, F. A. Structure of FlgK reveals the divergence of the bacterial Hook-Filament Junction of Campylobacter. *Scientific Reports* **7**, 15743, doi:10.1038/s41598-017-15837-0 (2017).

10 Bertoni, M., Kiefer, F., Biasini, M., Bordoli, L. & Schwede, T. Modeling protein quaternary structure of homo- and hetero-oligomers beyond binary interactions by homology. *Sci Rep* **7**, 10480, doi:10.1038/s41598-017-09654-8 (2017).
