# Supplementary material for: Large-scale discovery of protein interactions at residue resolution using co-evolution calculated from genomic sequences
Source: Nat Commun. 2021 Mar 2;12:1396. doi: 10.1038/s41467-021-21636-z (PMC7925567; doi:10.1038/s41467-021-21636-z)
Supplement: Supplementary file 2 — Description of Additional Supplementary Files [file 41467_2021_21636_MOESM2_ESM.pdf]

Description of Additional Supplementary Files

File Name: Supplementary Data 1
Description: *Escherichia coli* proteome monomer alignment statistics

File Name: Supplementary Data 2
Description: Positive Benchmark set of non-redundant protein complexes with structural resolution

File Name: Supplementary Data 3
Description: EVcomplex2 model parameters

File Name: Supplementary Data 4
Description: EVcomplex2 model benchmarking and score calibration table

File Name: Supplementary Data 5
Description: Comparison of EVcomplex2 to prior method performance on positive benchmark set

File Name: Supplementary Data 6
Description: Comparison to Cong et al, Science (2019)

File Name: Supplementary Data 7
Description: Predicted membrane protein Interactions

File Name: Supplementary Data 8
Description: Docking results for positive benchmark set

File Name: Supplementary Data 9
Description: Docking results for predicted interactions

File Name: Supplementary Data 10
Description: Docked models for positive benchmark set

File Name: Supplementary Data 11
Description: Docked models for predicted interactions
