# Supplementary figures and images for: Large-scale discovery of protein interactions at residue resolution using co-evolution calculated from genomic sequences
Source: Nat Commun. 2021 Mar 2;12:1396. doi: 10.1038/s41467-021-21636-z (PMC7925567; doi:10.1038/s41467-021-21636-z)

## ECFA2\_LACBA-ECFT\_LACBA

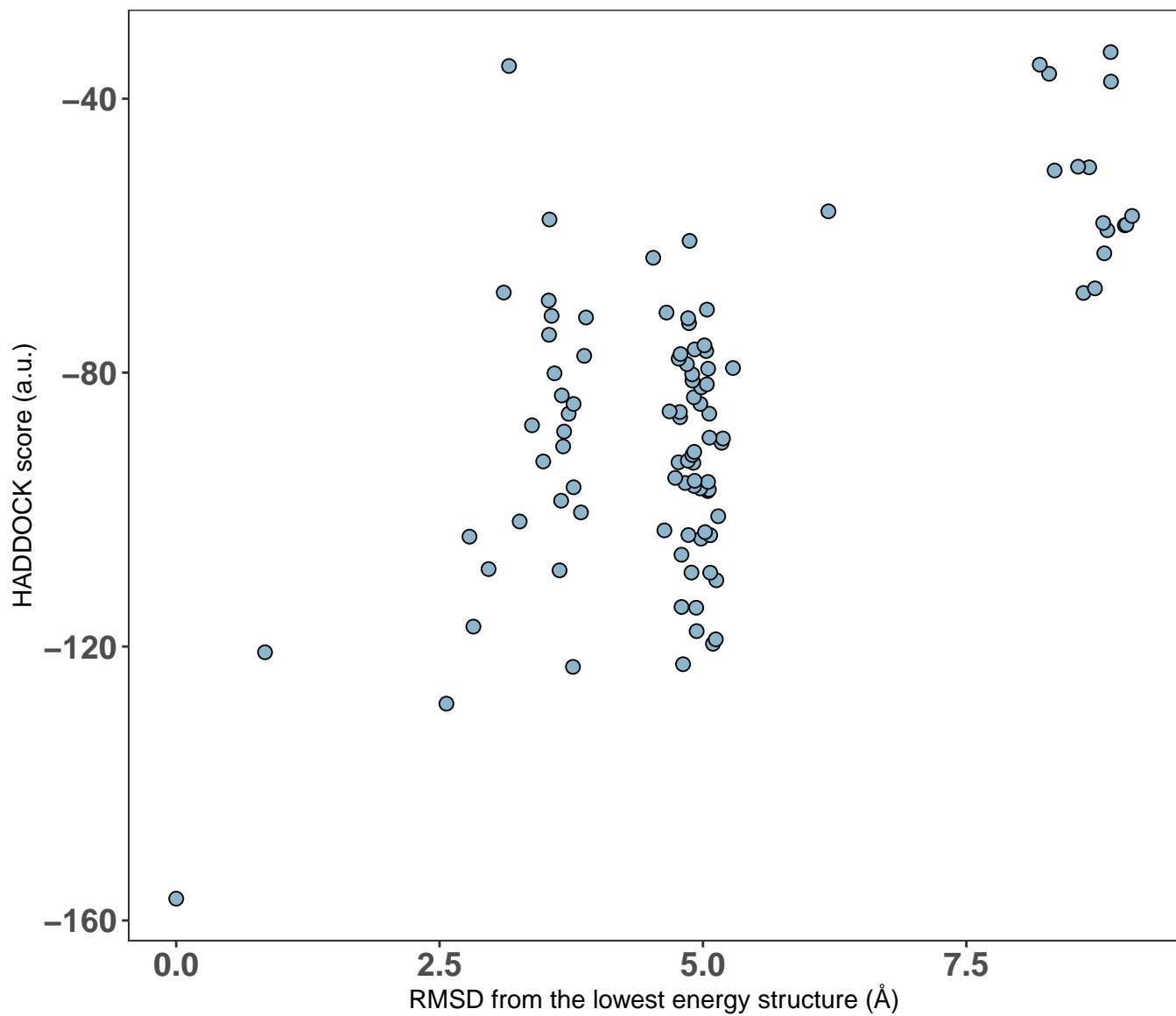

Supplement: Supplementary file 12 — Supplementary Data 10 [file 41467_2021_21636_MOESM12_ESM.zip › supplementary/allpdb0162_ECFA2_ECFT/allpdb0162_ECFA2_ECFT.pdf]

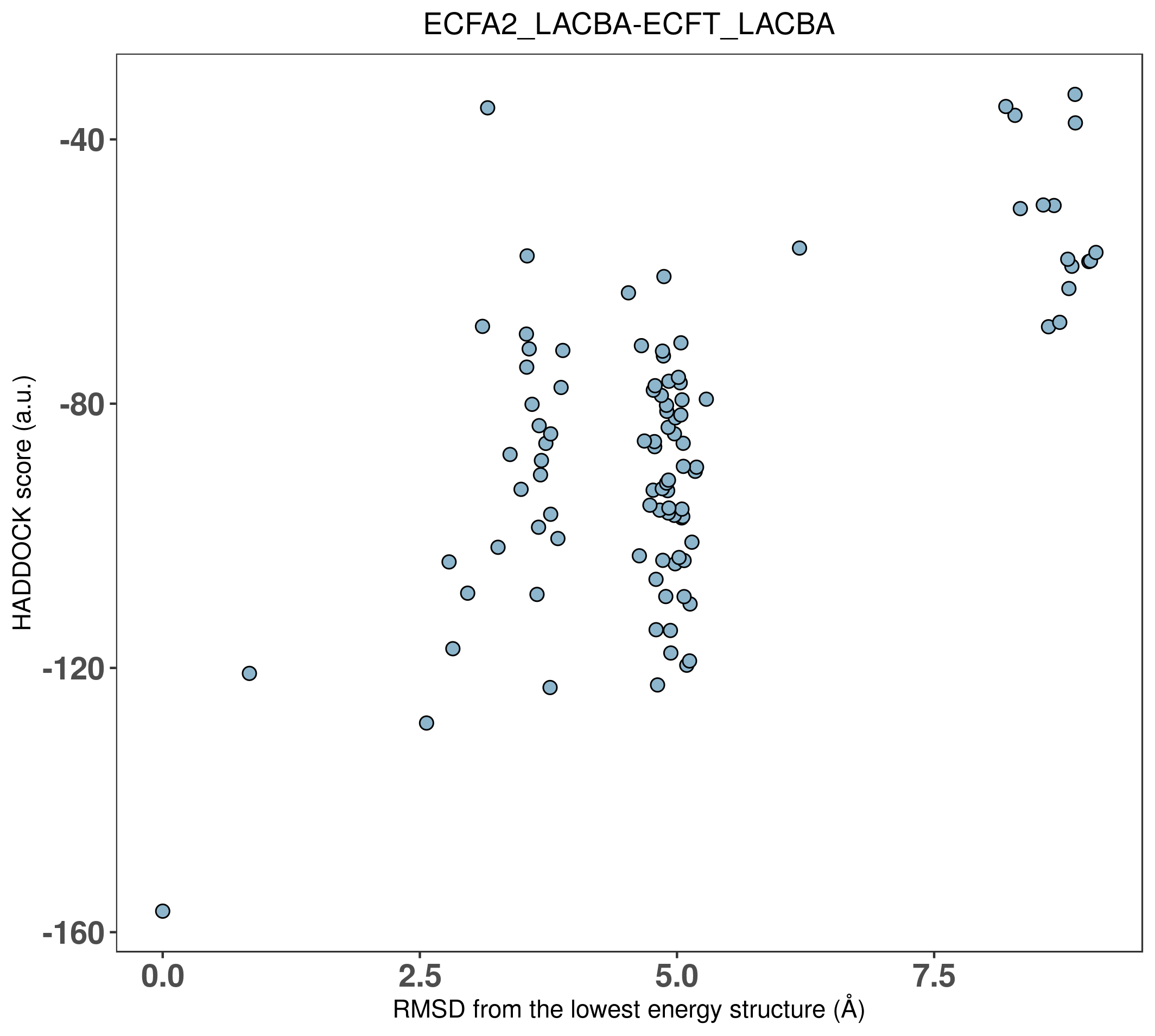

Supplement: Supplementary file 12 — Supplementary Data 10 [file 41467_2021_21636_MOESM12_ESM.zip › supplementary/allpdb0162_ECFA2_ECFT/allpdb0162_ECFA2_ECFT.png]

HIGA\_ECOLI-HIGB\_ECOLI

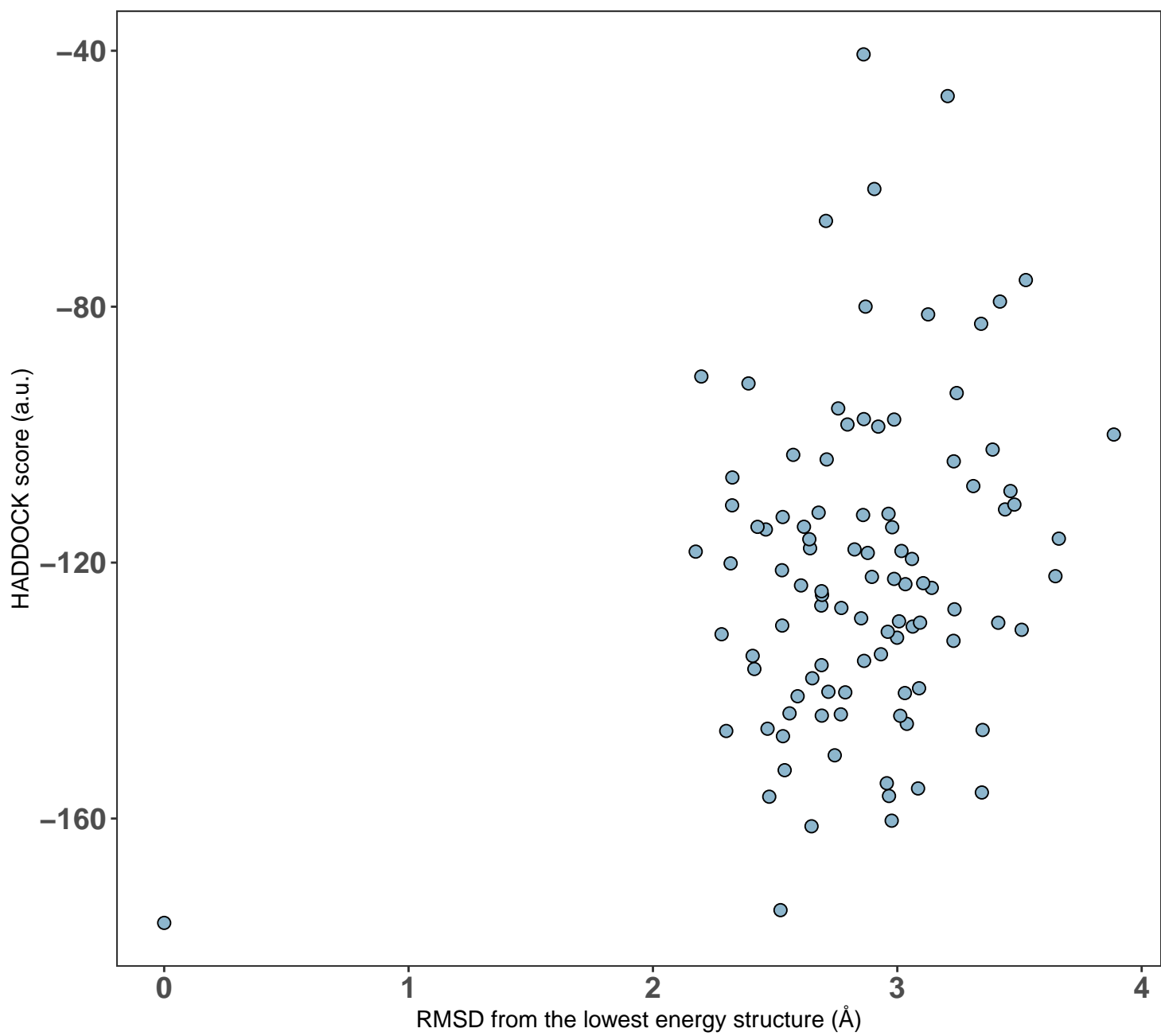

Supplement: Supplementary file 12 — Supplementary Data 10 [file 41467_2021_21636_MOESM12_ESM.zip › supplementary/allpdb1601_HIGA_HIGB/allpdb1601_HIGA_HIGB.pdf]

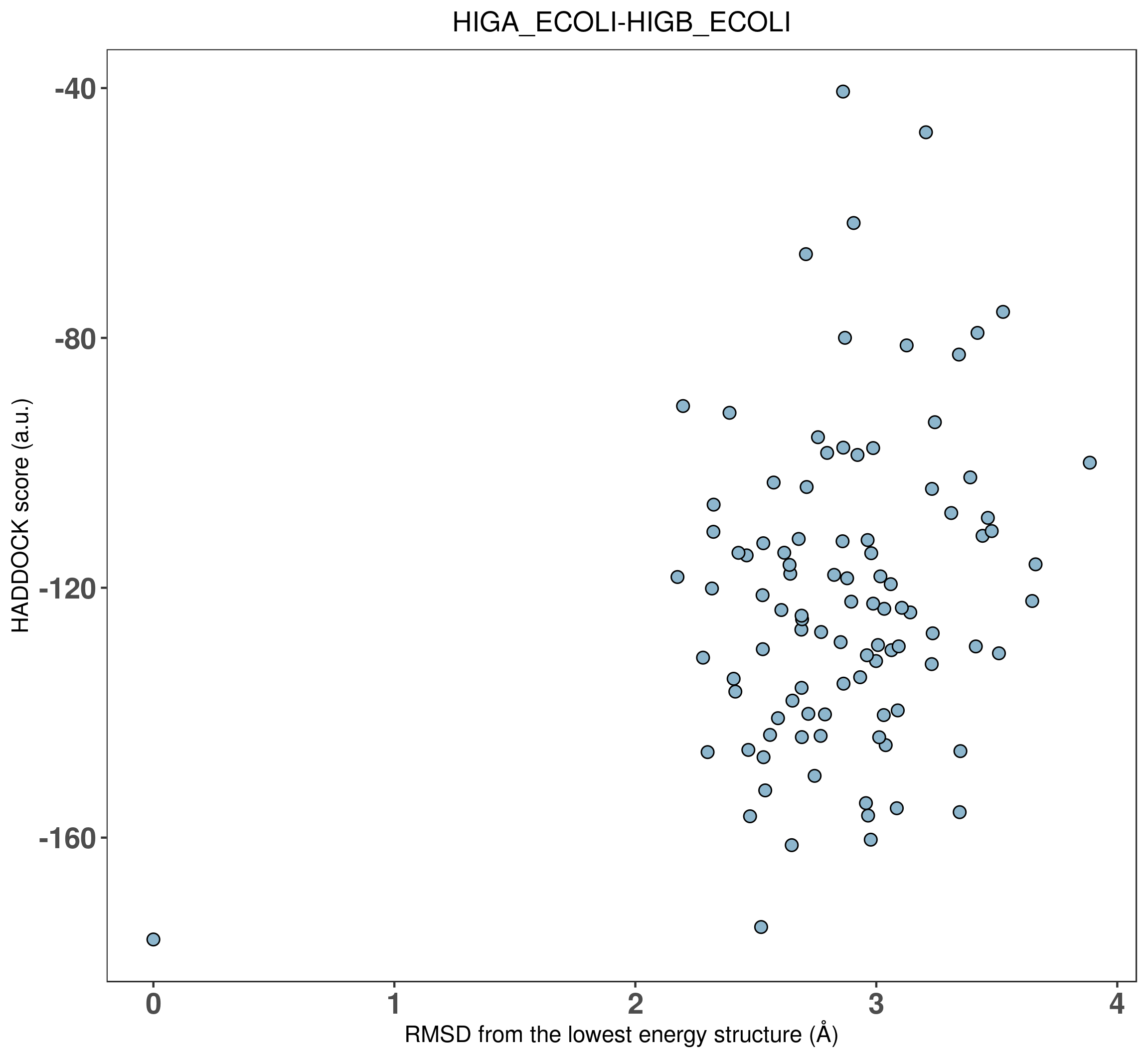

Supplement: Supplementary file 12 — Supplementary Data 10 [file 41467_2021_21636_MOESM12_ESM.zip › supplementary/allpdb1601_HIGA_HIGB/allpdb1601_HIGA_HIGB.png]

CASC\_ECOLI-CAS5\_ECOLI

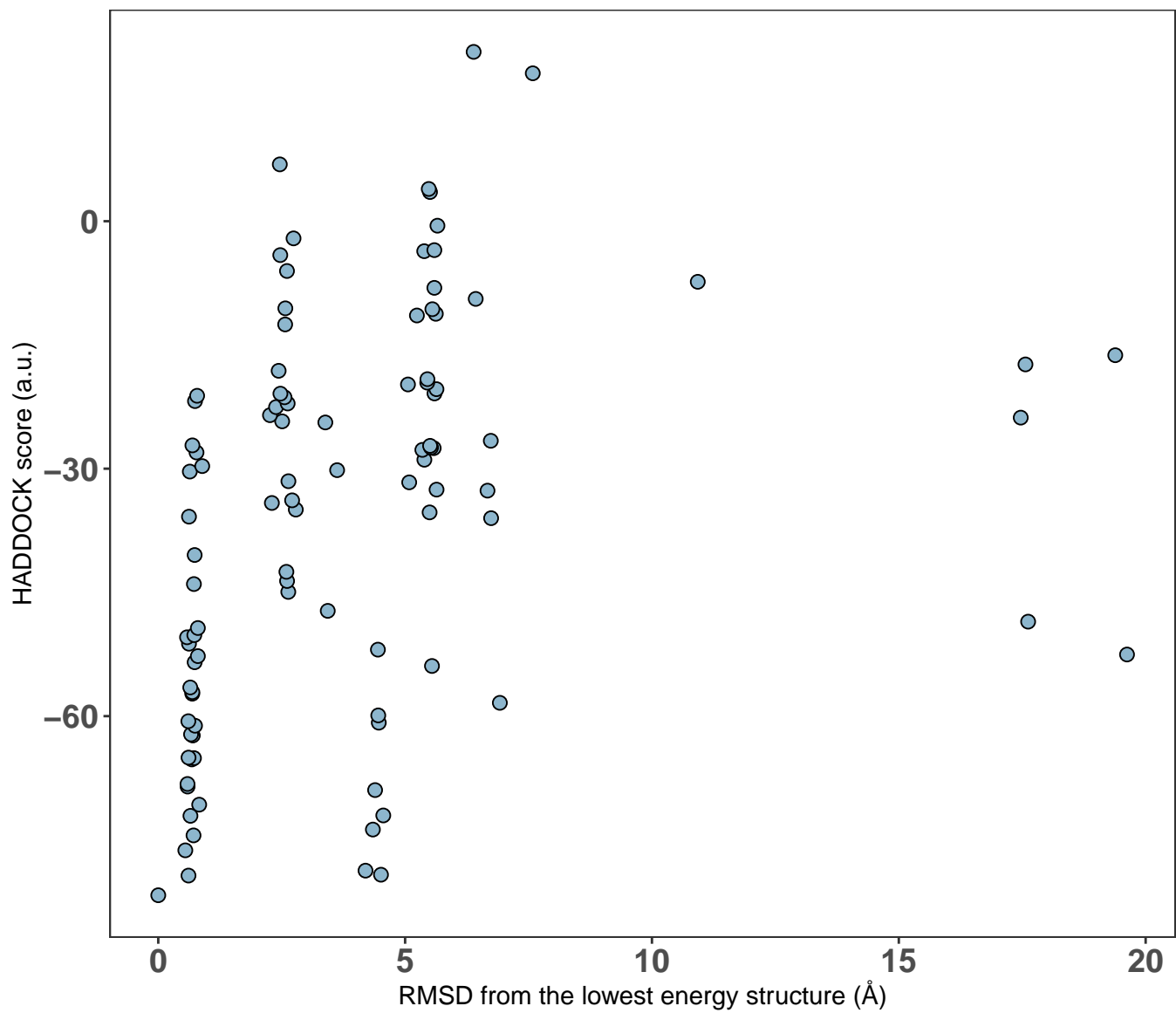

Supplement: Supplementary file 12 — Supplementary Data 10 [file 41467_2021_21636_MOESM12_ESM.zip › supplementary/allpdb0172_CASC_CAS5/allpdb0172_CASC_CAS5.pdf]

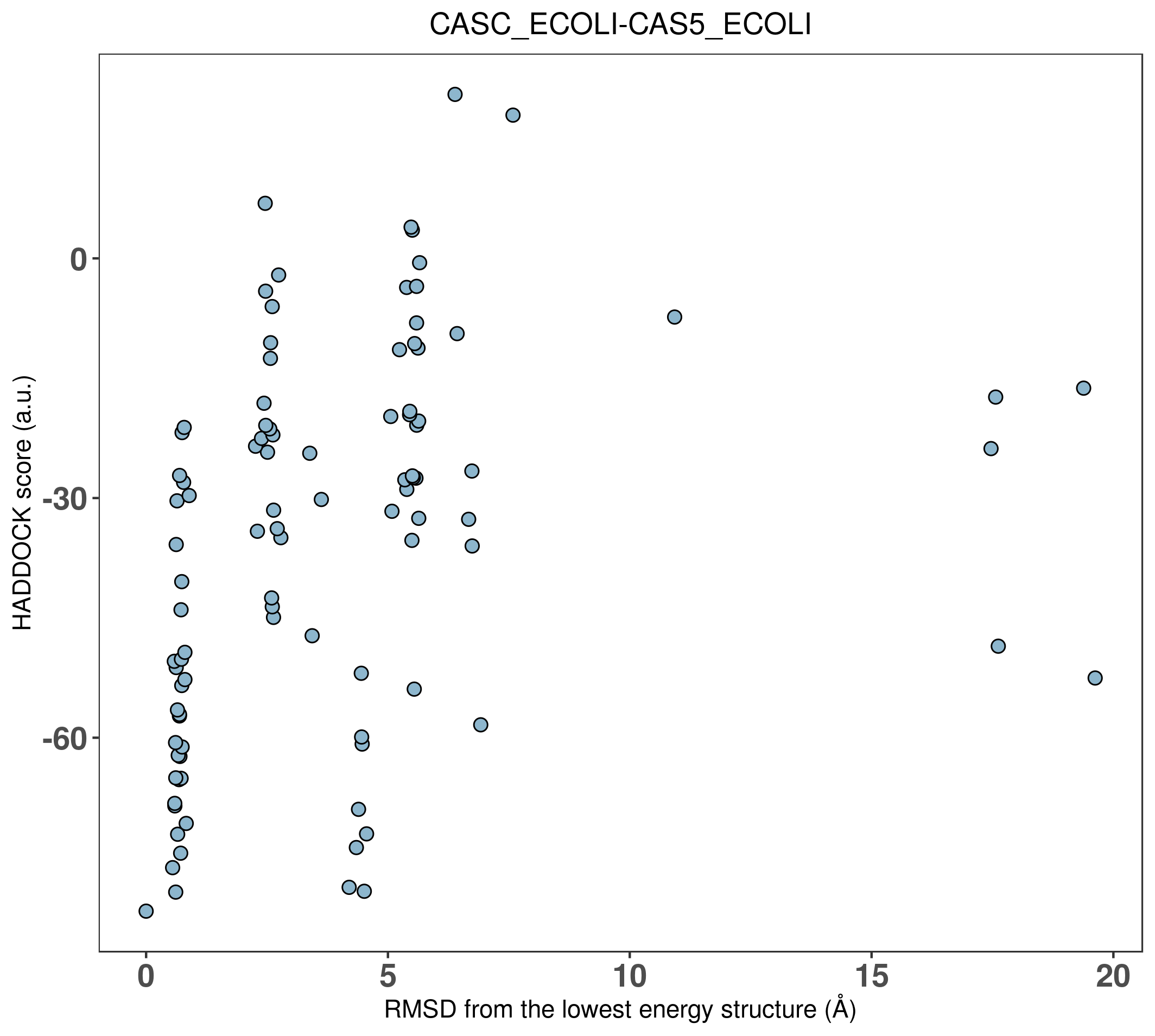

Supplement: Supplementary file 12 — Supplementary Data 10 [file 41467_2021_21636_MOESM12_ESM.zip › supplementary/allpdb0172_CASC_CAS5/allpdb0172_CASC_CAS5.png]

C4YDZ9\_CANAW-C4YFL7\_CANAW

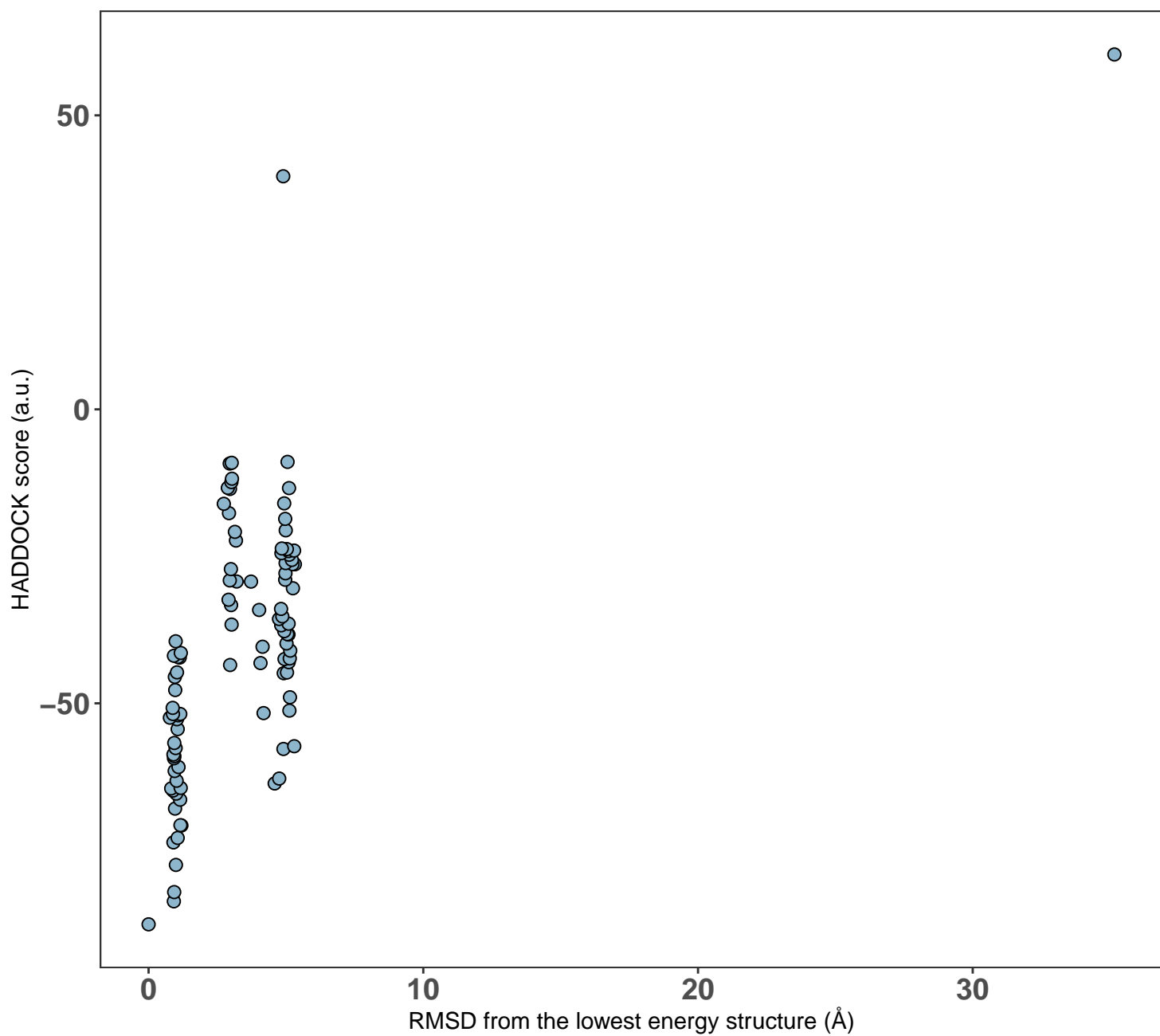

Supplement: Supplementary file 12 — Supplementary Data 10 [file 41467_2021_21636_MOESM12_ESM.zip › supplementary/allpdb1629_C4YDZ9_C4YFL7/allpdb1629_C4YDZ9_C4YFL7.pdf]

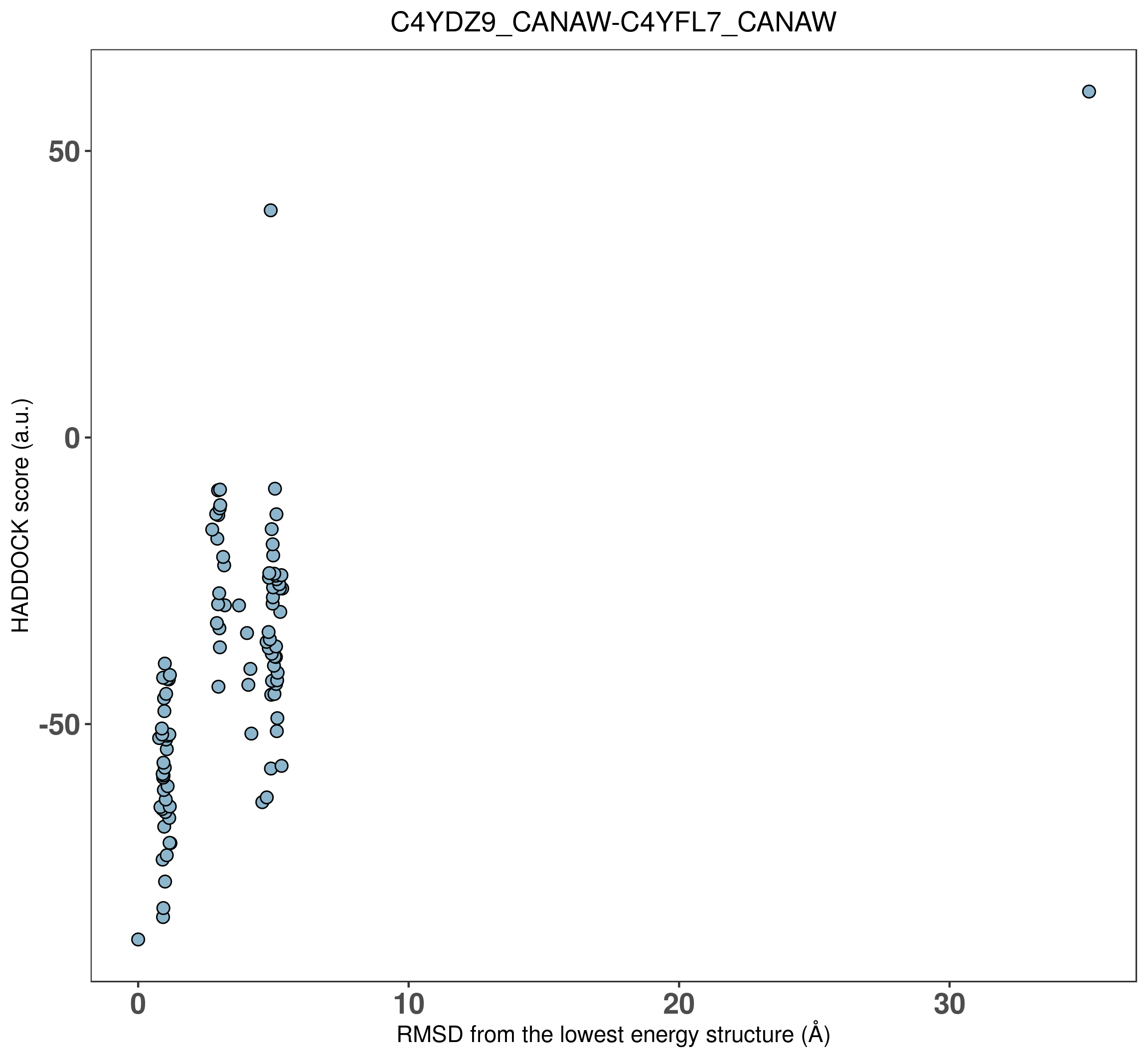

Supplement: Supplementary file 12 — Supplementary Data 10 [file 41467_2021_21636_MOESM12_ESM.zip › supplementary/allpdb1629_C4YDZ9_C4YFL7/allpdb1629_C4YDZ9_C4YFL7.png]

SCNA\_THITI-SCNC\_THITI

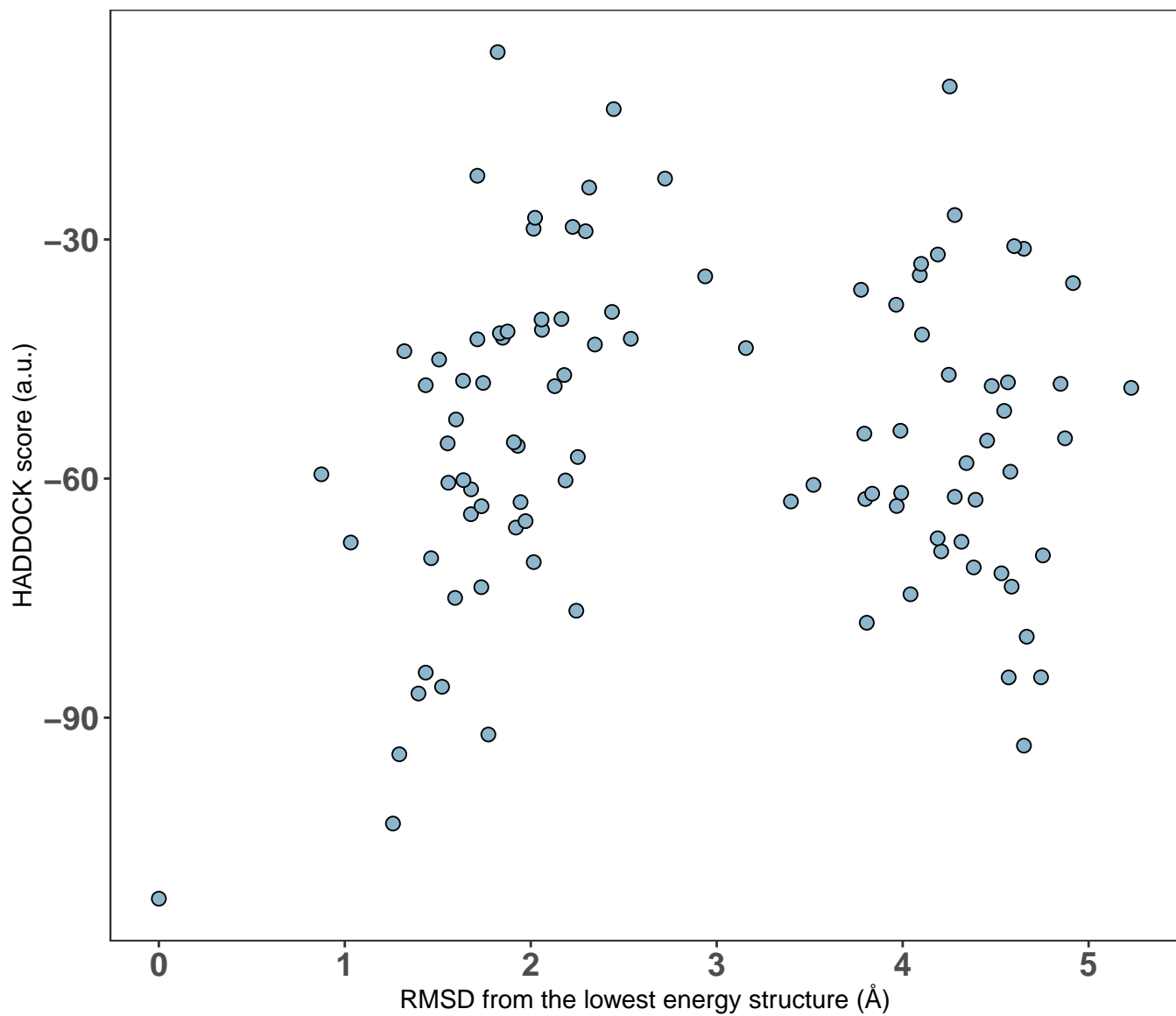

Supplement: Supplementary file 12 — Supplementary Data 10 [file 41467_2021_21636_MOESM12_ESM.zip › supplementary/allpdb0203_SCNA_SCNC/allpdb0203_SCNA_SCNC.pdf]

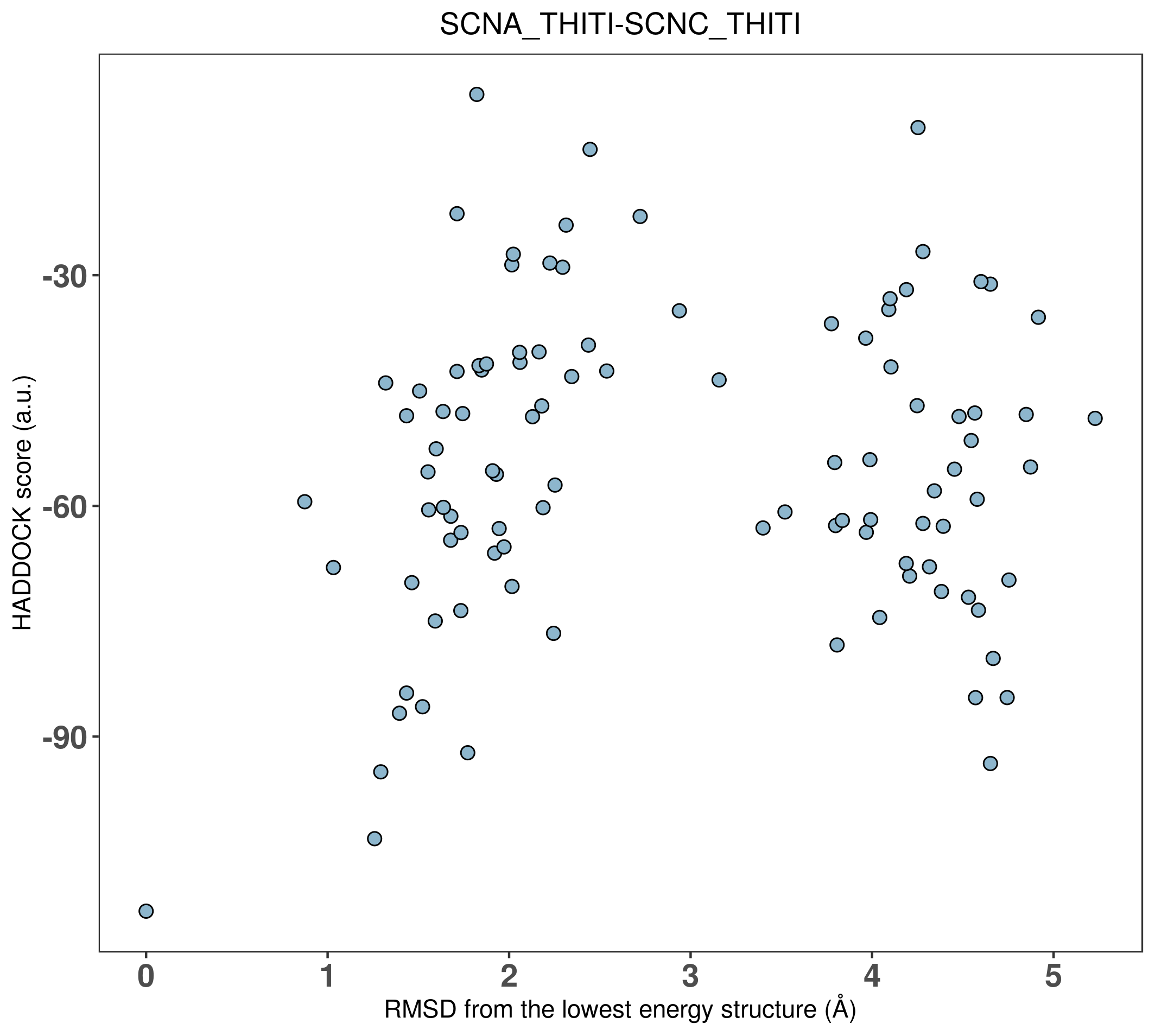

Supplement: Supplementary file 12 — Supplementary Data 10 [file 41467_2021_21636_MOESM12_ESM.zip › supplementary/allpdb0203_SCNA_SCNC/allpdb0203_SCNA_SCNC.png]

Q72GS0\_THET2-Q72GR9\_THET2

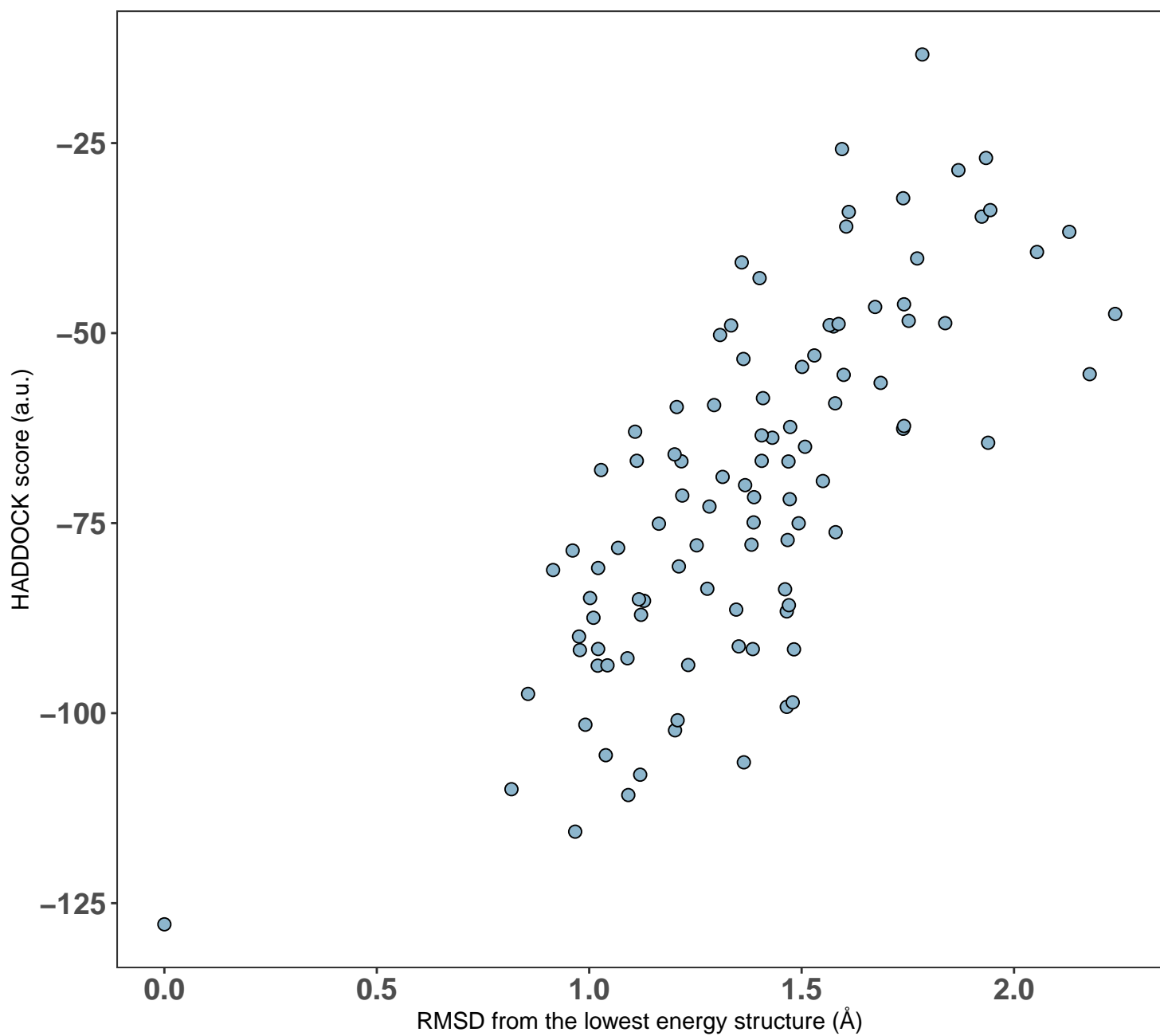

Supplement: Supplementary file 12 — Supplementary Data 10 [file 41467_2021_21636_MOESM12_ESM.zip › supplementary/allpdb1670_Q72GS0_Q72GR9/allpdb1670_Q72GS0_Q72GR9.pdf]

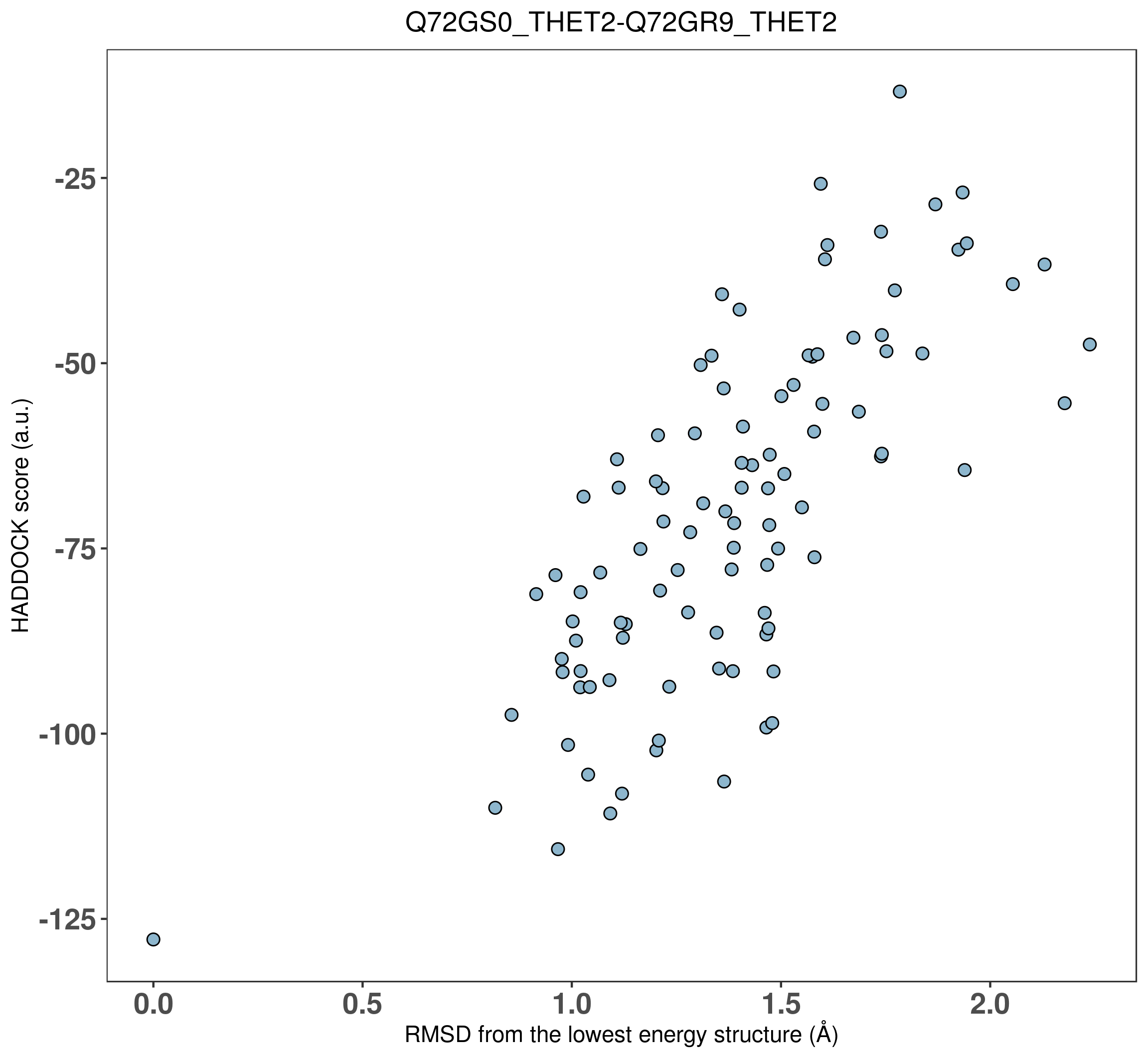

Supplement: Supplementary file 12 — Supplementary Data 10 [file 41467_2021_21636_MOESM12_ESM.zip › supplementary/allpdb1670_Q72GS0_Q72GR9/allpdb1670_Q72GS0_Q72GR9.png]

RRP45\_YEAST-RRP40\_YEAST

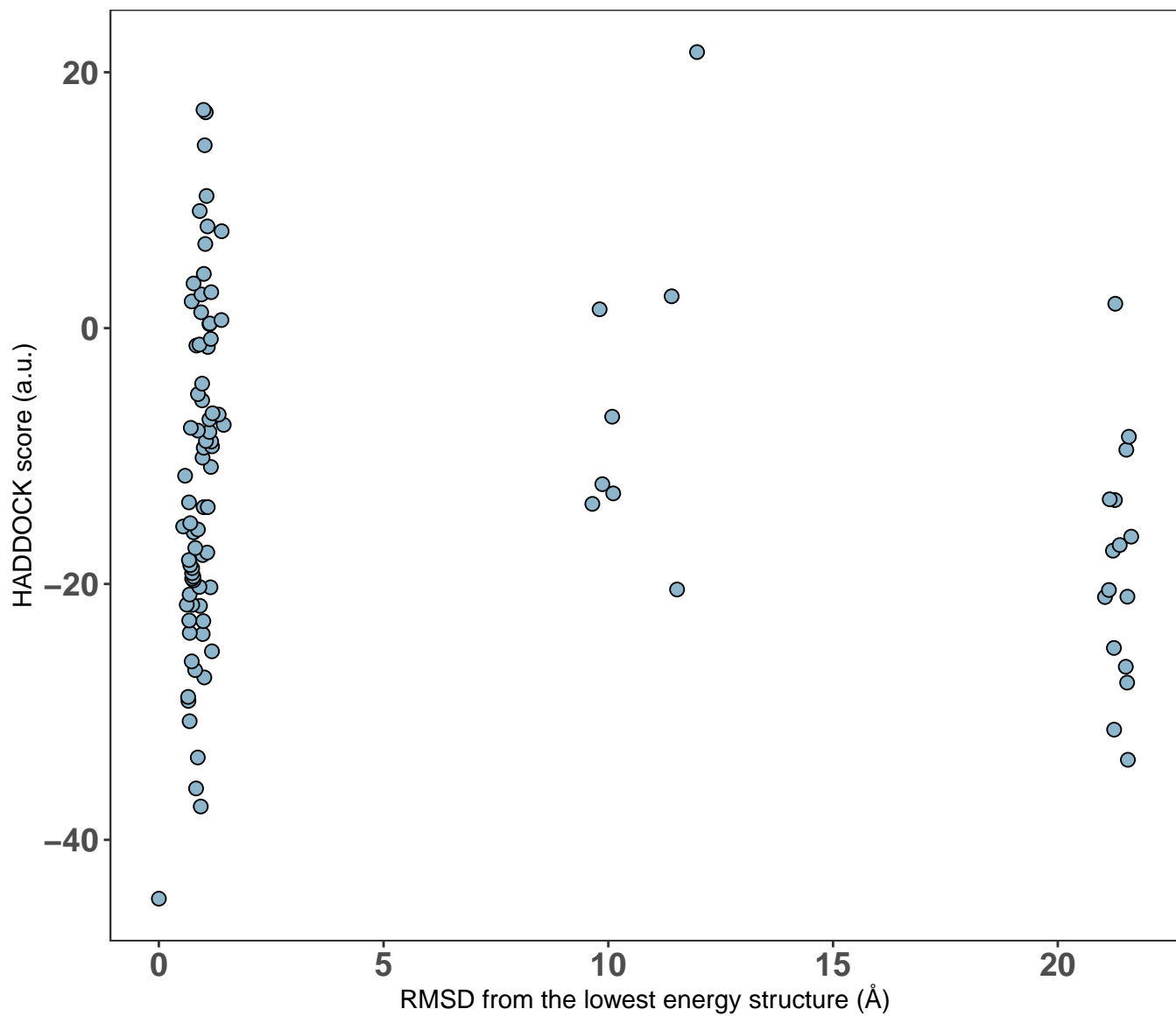

Supplement: Supplementary file 12 — Supplementary Data 10 [file 41467_2021_21636_MOESM12_ESM.zip › supplementary/allpdb0007_RRP45_RRP40/allpdb0007_RRP45_RRP40.pdf]

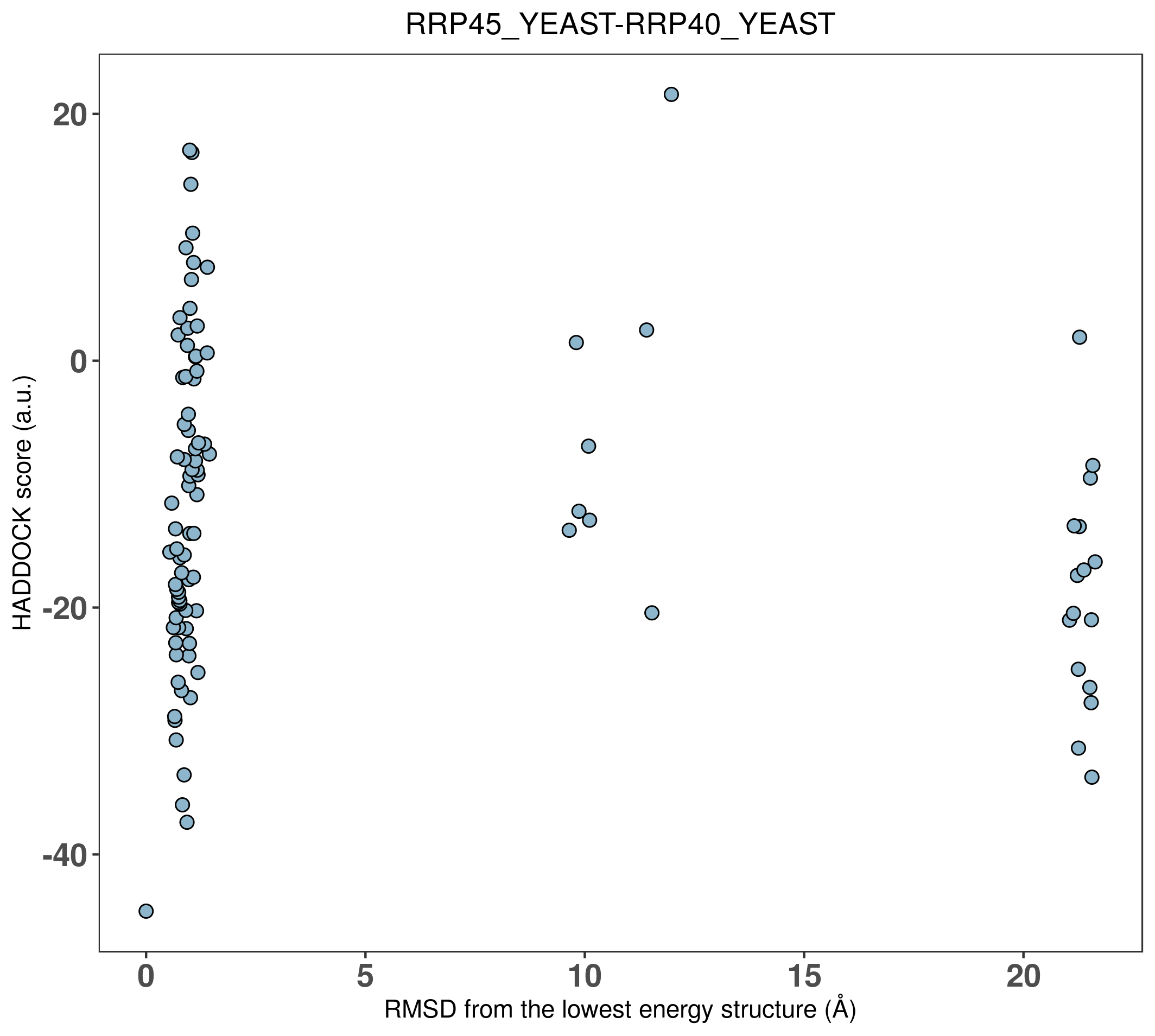

Supplement: Supplementary file 12 — Supplementary Data 10 [file 41467_2021_21636_MOESM12_ESM.zip › supplementary/allpdb0007_RRP45_RRP40/allpdb0007_RRP45_RRP40.png]

## H2B1J\_HUMAN-H4\_HUMAN

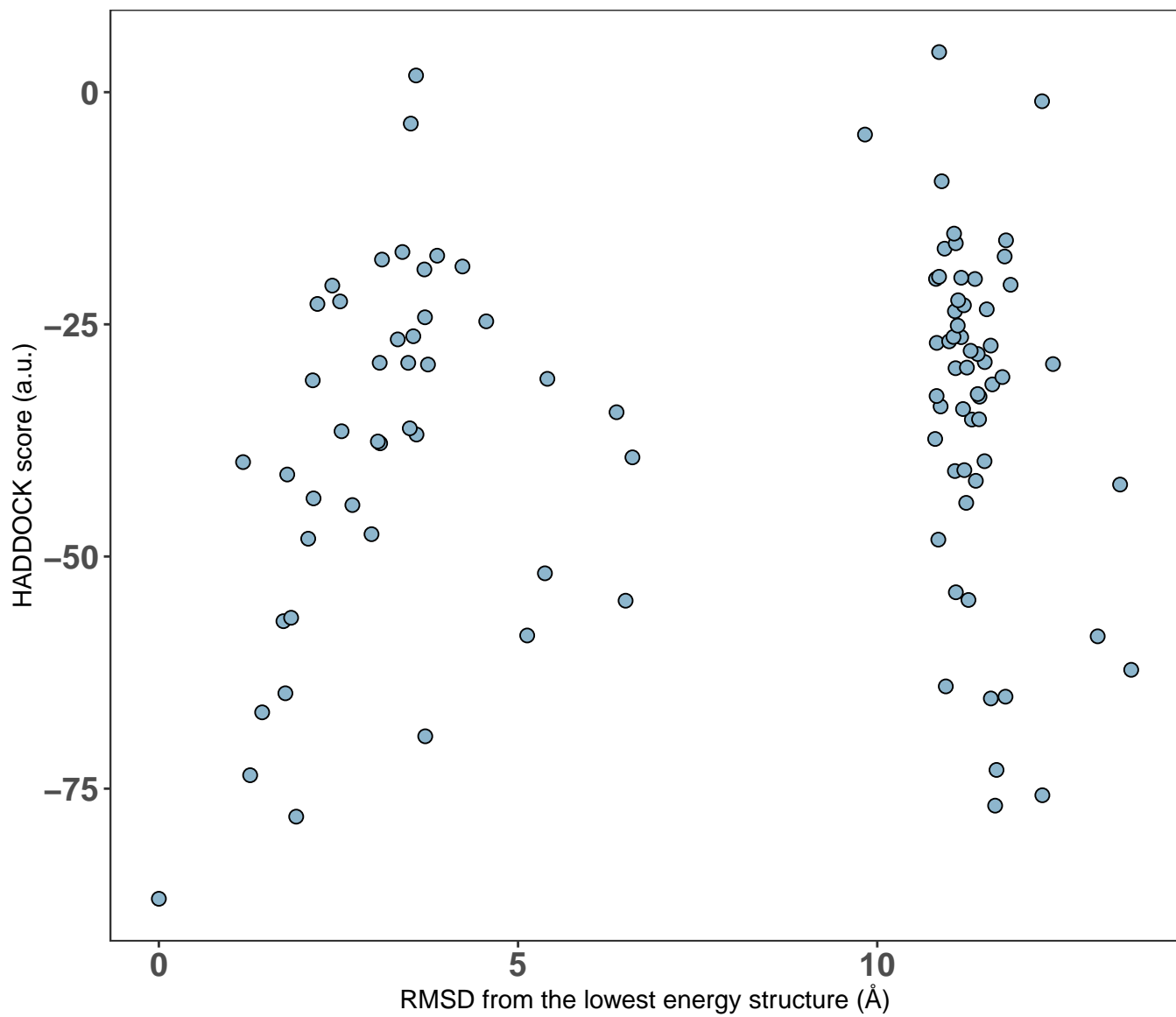

Supplement: Supplementary file 12 — Supplementary Data 10 [file 41467_2021_21636_MOESM12_ESM.zip › supplementary/allpdb0037_H2B1J_H4/allpdb0037_H2B1J_H4.pdf]

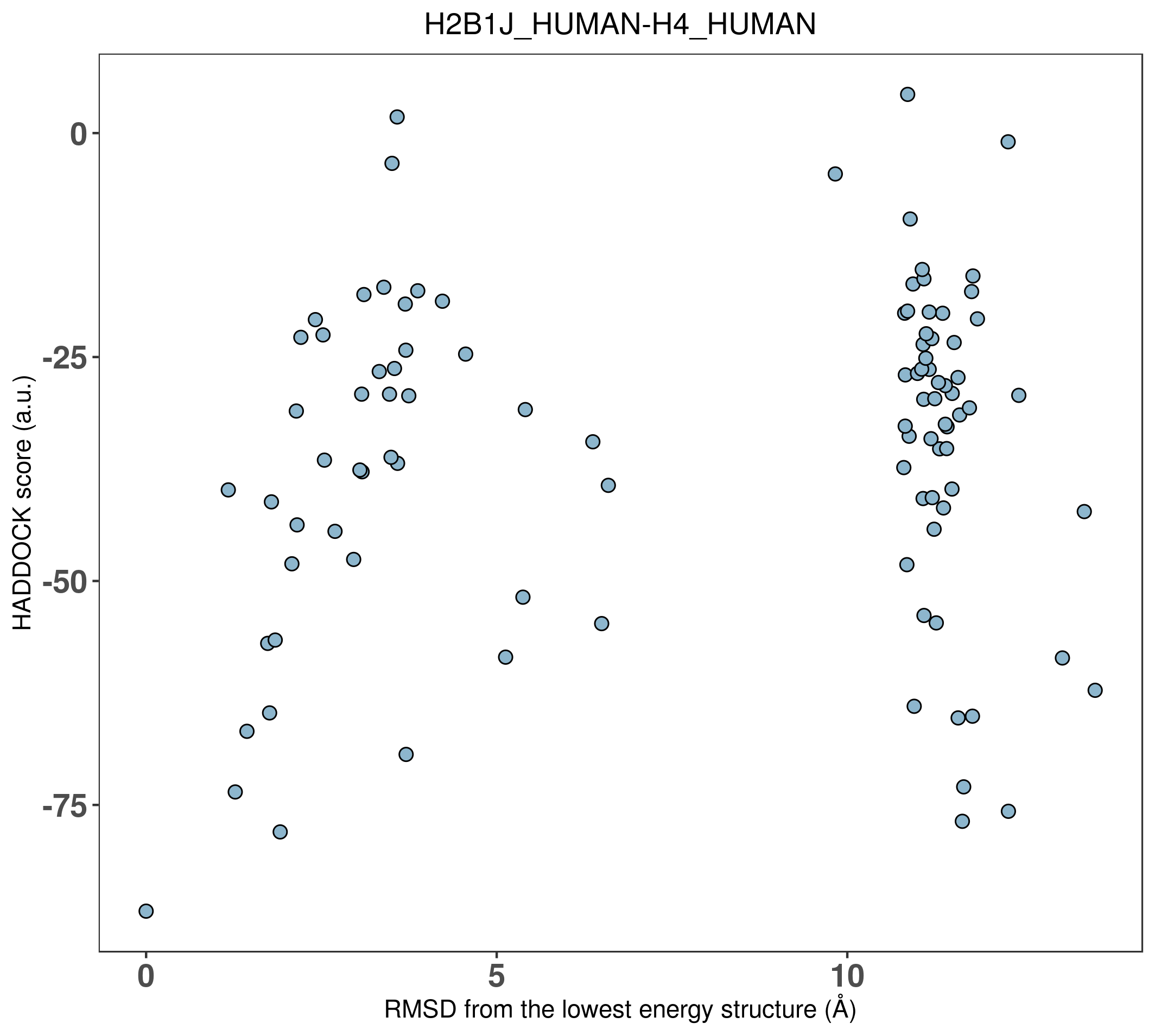

Supplement: Supplementary file 12 — Supplementary Data 10 [file 41467_2021_21636_MOESM12_ESM.zip › supplementary/allpdb0037_H2B1J_H4/allpdb0037_H2B1J_H4.png]

CY1\_BOVIN-QCR6\_BOVIN

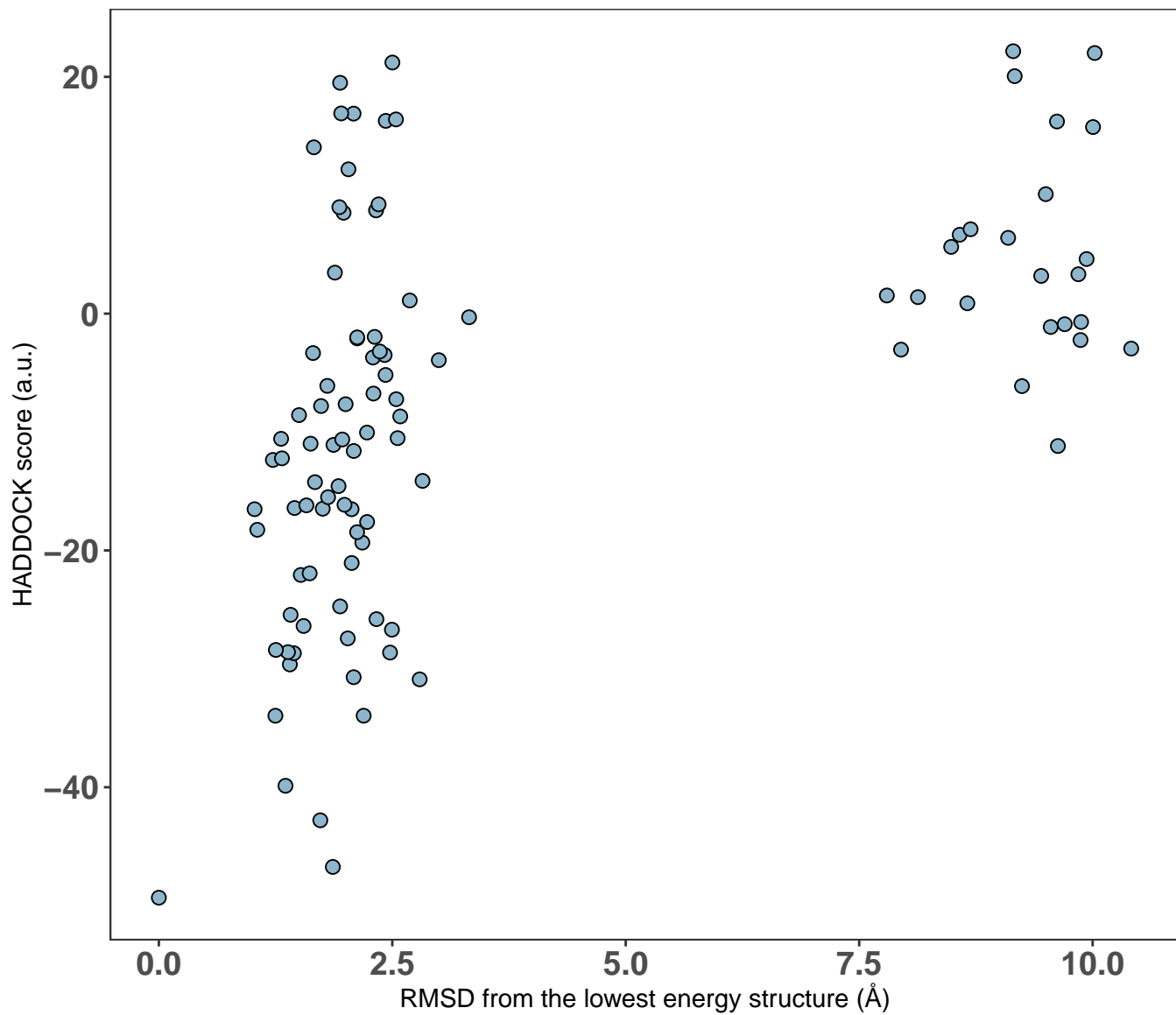

Supplement: Supplementary file 12 — Supplementary Data 10 [file 41467_2021_21636_MOESM12_ESM.zip › supplementary/allpdb0039_CY1_QCR6/allpdb0039_CY1_QCR6.pdf]

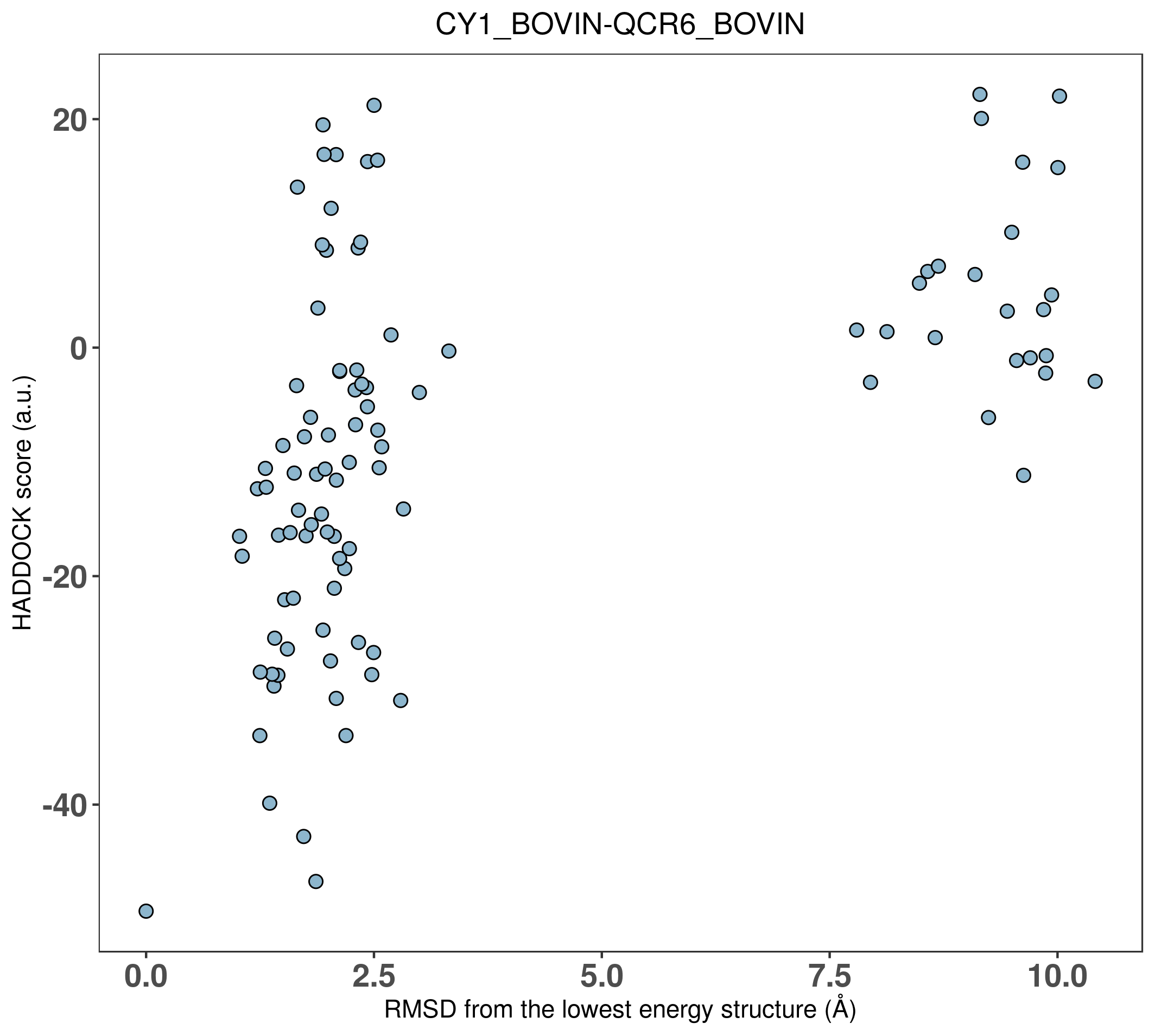

Supplement: Supplementary file 12 — Supplementary Data 10 [file 41467_2021_21636_MOESM12_ESM.zip › supplementary/allpdb0039_CY1_QCR6/allpdb0039_CY1_QCR6.png]

YAJC\_ECOLI-LPTG\_ECOLI

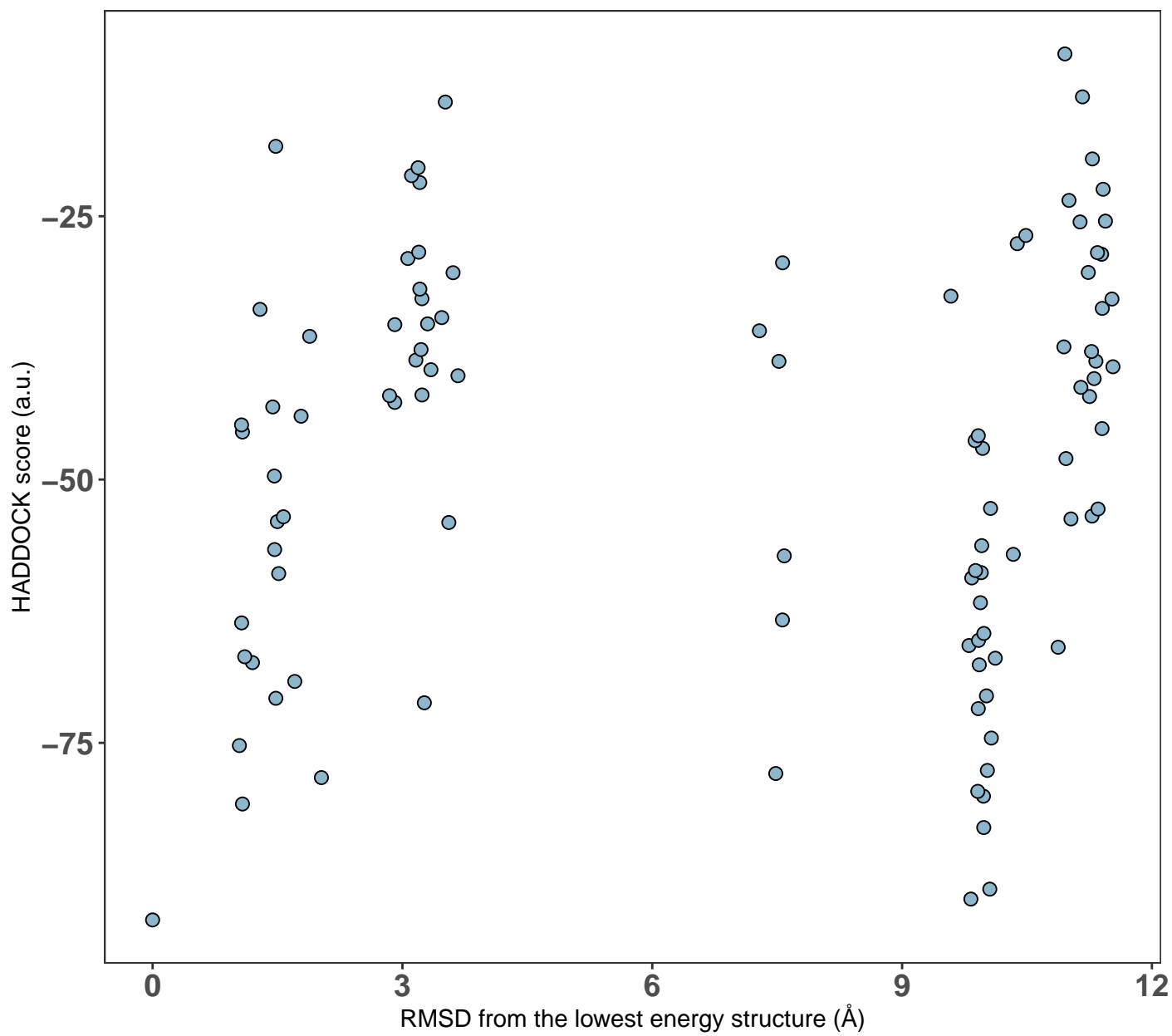

Supplement: Supplementary file 13 — Supplementary Data 11 [file 41467_2021_21636_MOESM13_ESM.zip › supplementary_prediction/membrane088588_YAJC_LPTG/membrane088588_YAJC_LPTG.pdf]

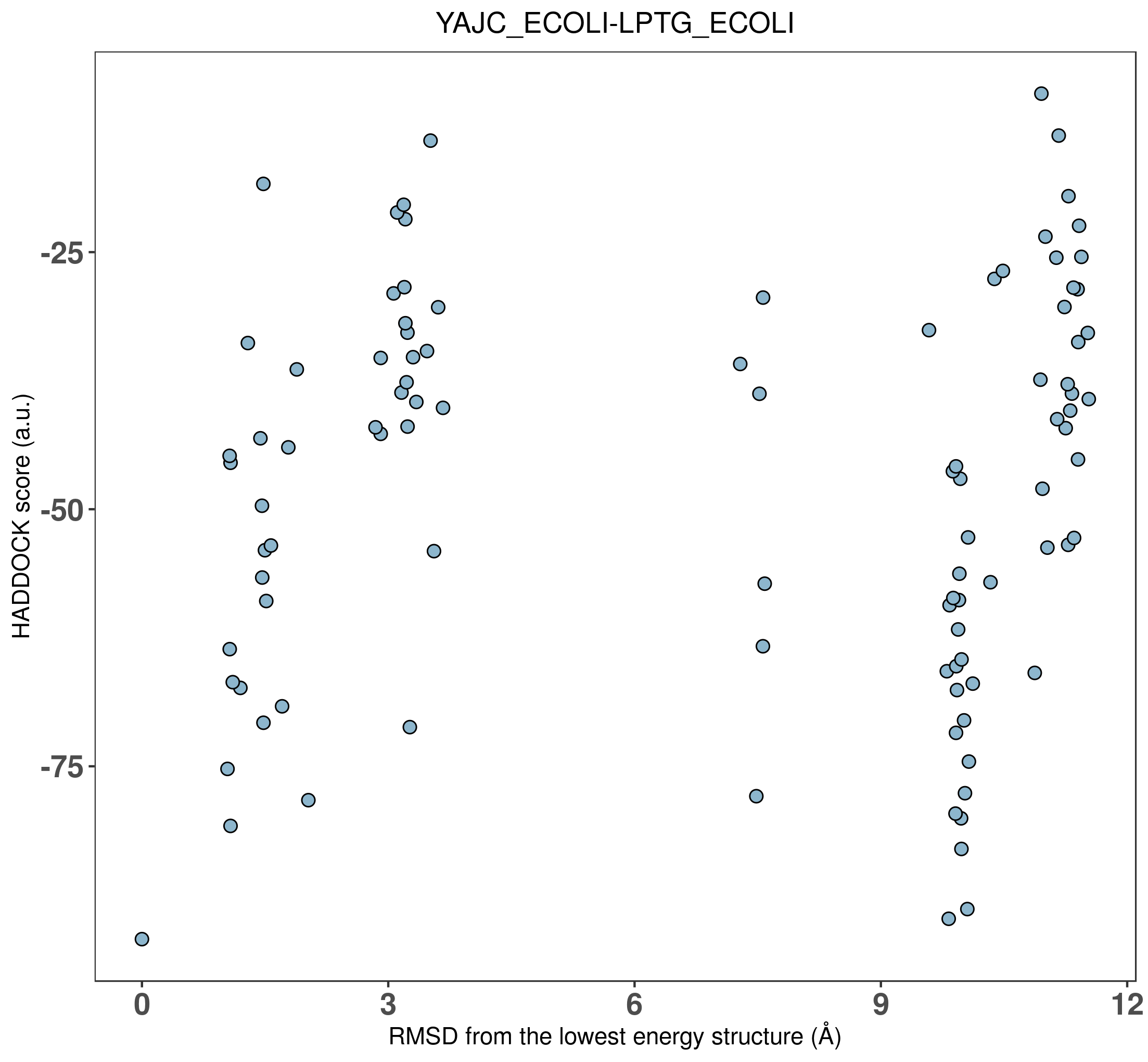

Supplement: Supplementary file 13 — Supplementary Data 11 [file 41467_2021_21636_MOESM13_ESM.zip › supplementary_prediction/membrane088588_YAJC_LPTG/membrane088588_YAJC_LPTG.png]

## YAJC\_ECOLI-LPTG\_ECOLI

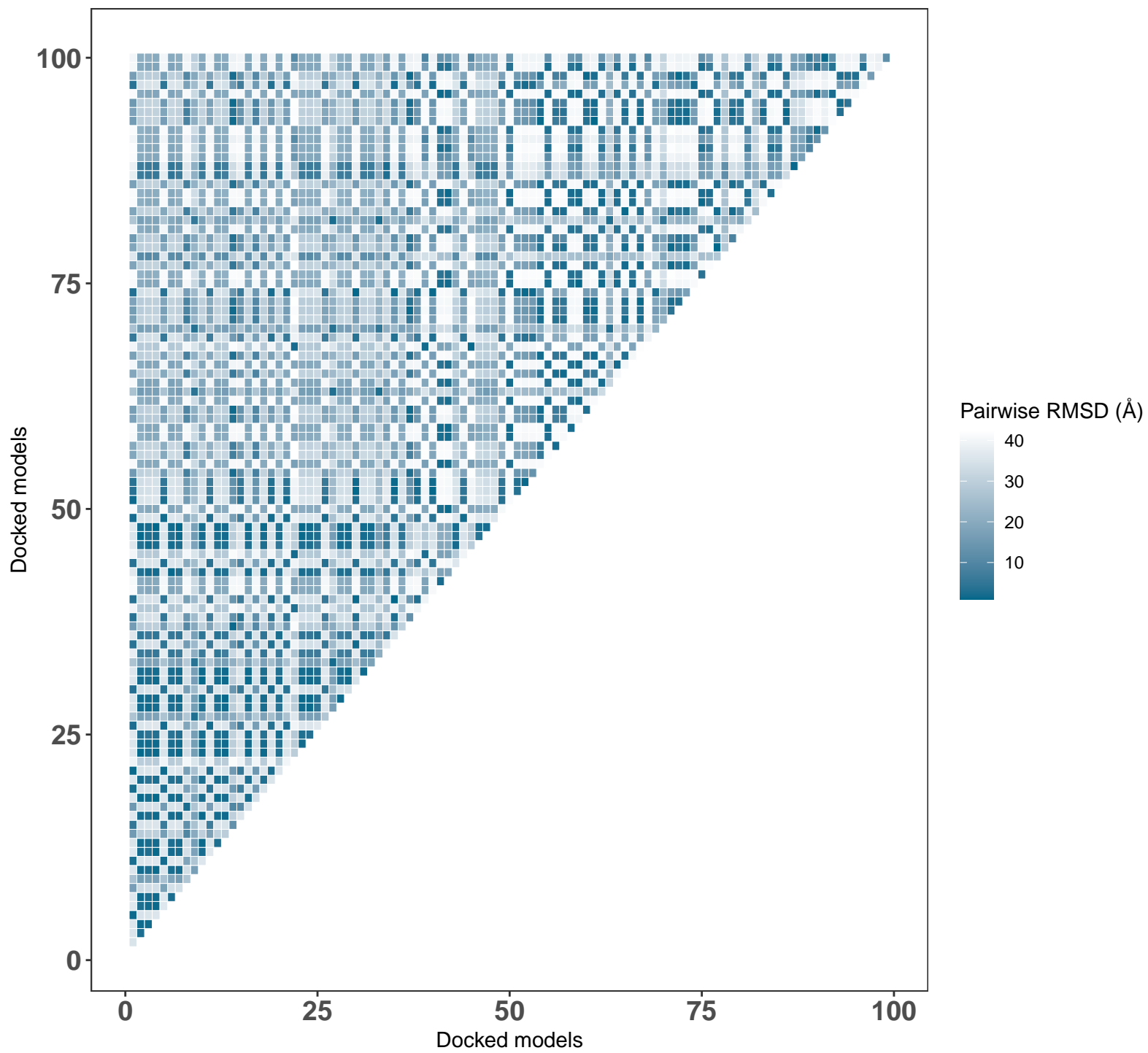

Supplement: Supplementary file 13 — Supplementary Data 11 [file 41467_2021_21636_MOESM13_ESM.zip › supplementary_prediction/membrane088588_YAJC_LPTG/pwrmsd_membrane088588_YAJC_LPTG.pdf]

## TOLB\_ECOLI-PAL\_ECOLI

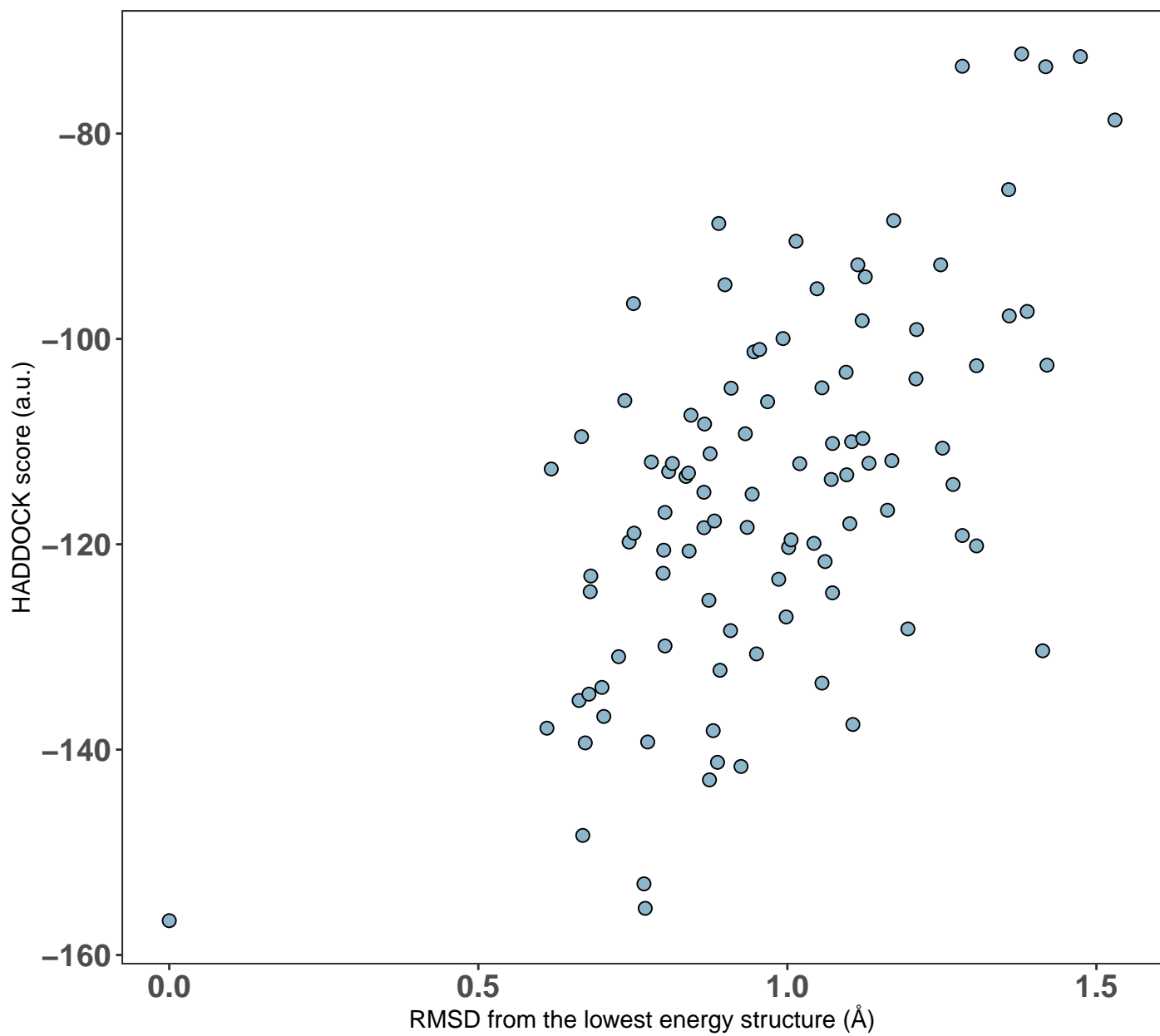

Supplement: Supplementary file 13 — Supplementary Data 11 [file 41467_2021_21636_MOESM13_ESM.zip › supplementary_prediction/cep_tp00839_TOLB_PAL/cep_tp00839_TOLB_PAL.pdf]

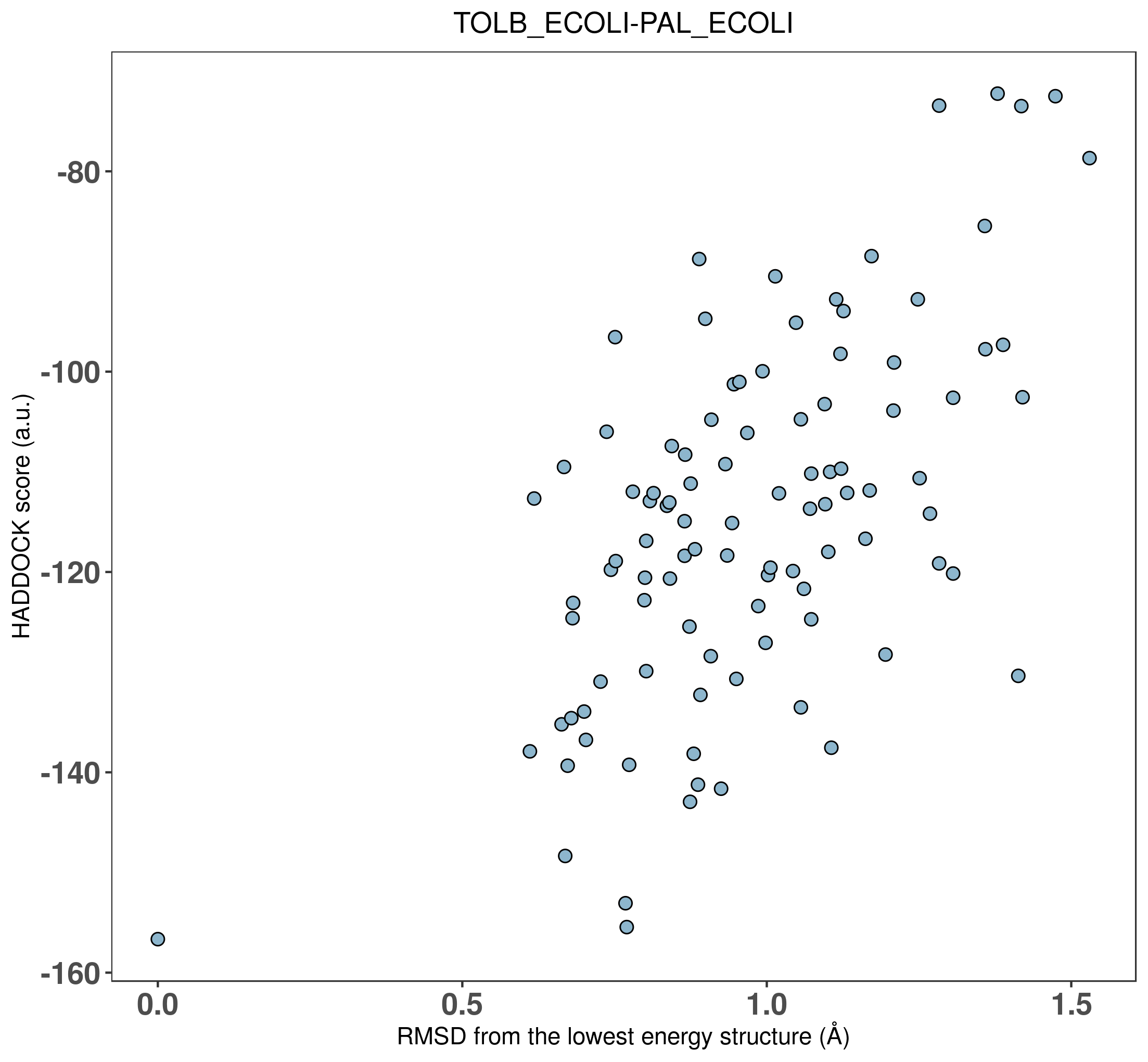

Supplement: Supplementary file 13 — Supplementary Data 11 [file 41467_2021_21636_MOESM13_ESM.zip › supplementary_prediction/cep_tp00839_TOLB_PAL/cep_tp00839_TOLB_PAL.png]

## TOLB\_ECOLI-PAL\_ECOLI

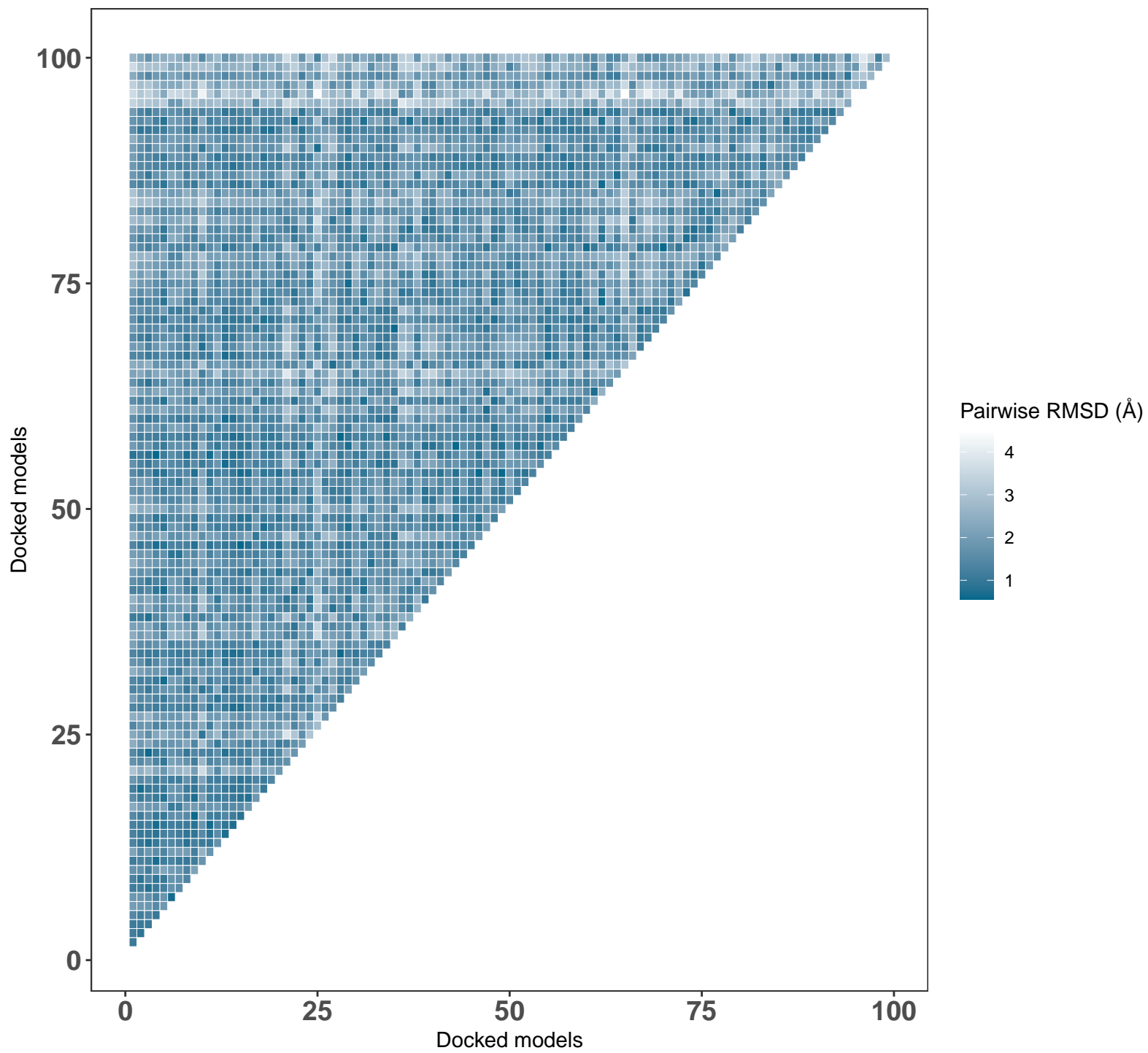

Supplement: Supplementary file 13 — Supplementary Data 11 [file 41467_2021_21636_MOESM13_ESM.zip › supplementary_prediction/cep_tp00839_TOLB_PAL/pwrmsd_cep_tp00839_TOLB_PAL.pdf]

## KDPC\_ECOLI-KDPB\_ECOLI

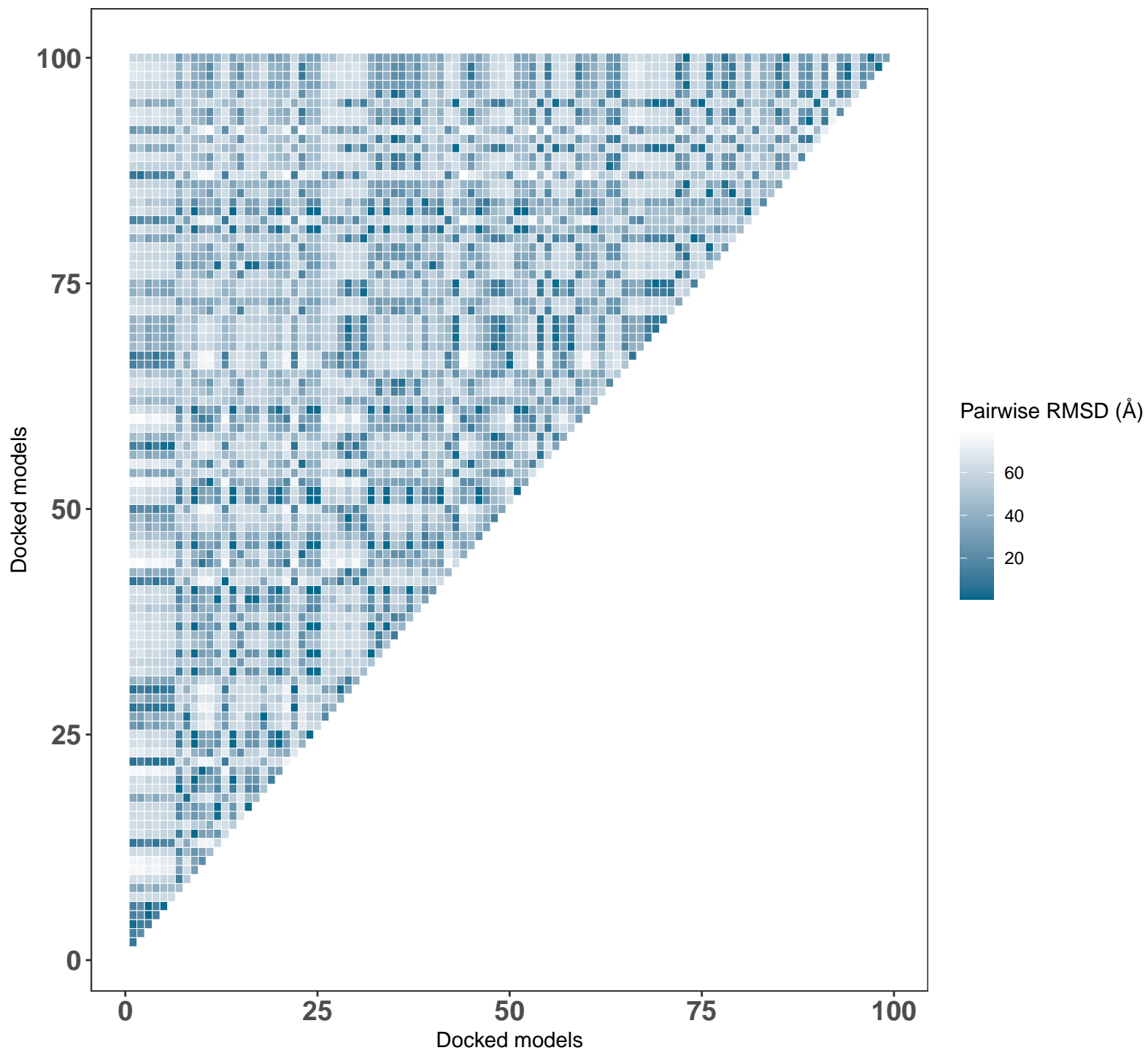

Supplement: Supplementary file 13 — Supplementary Data 11 [file 41467_2021_21636_MOESM13_ESM.zip › supplementary_prediction/cep_tp00607_KDPC_KDPB/pwrmsd_cep_tp00607_KDPC_KDPB.pdf]

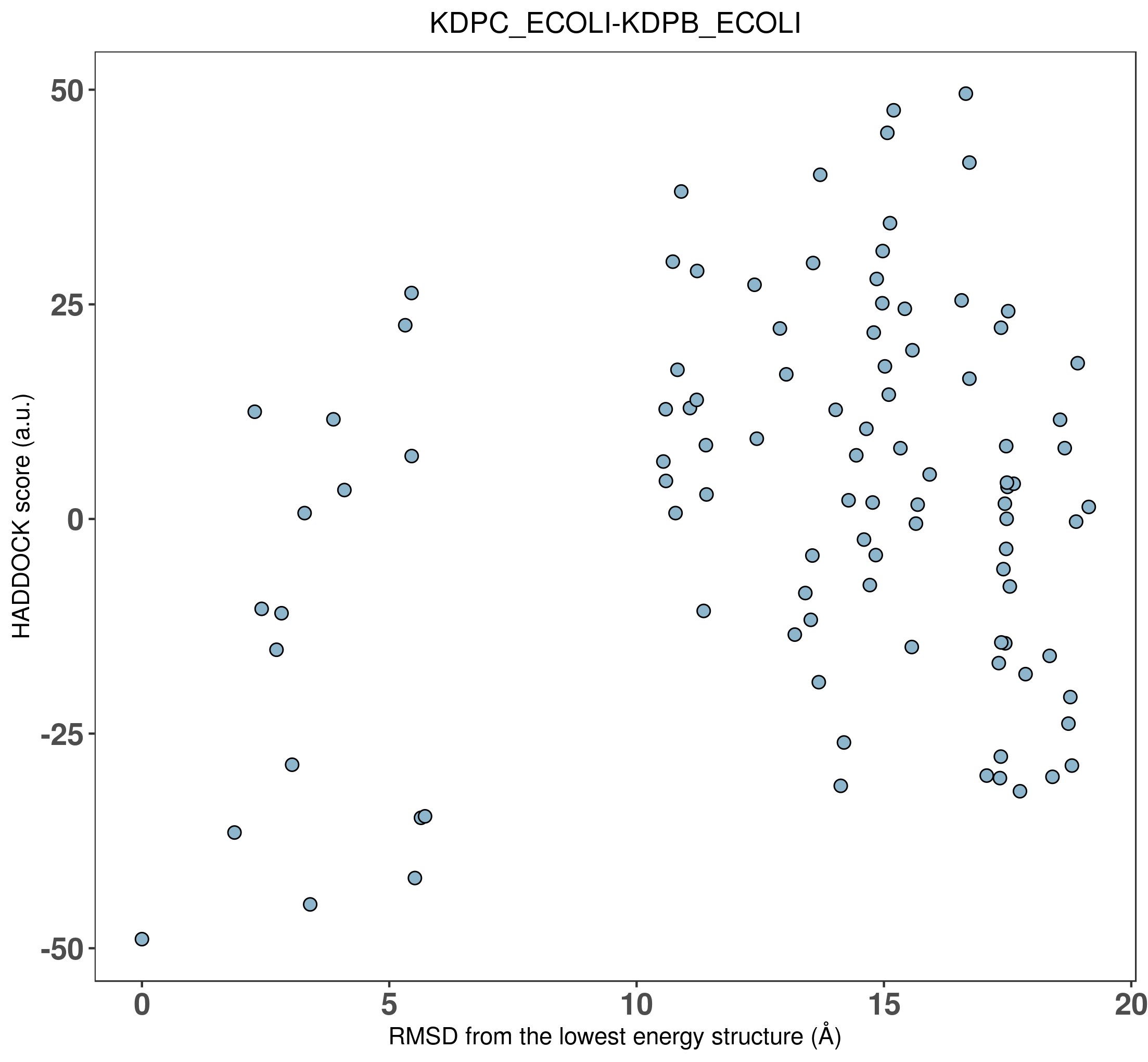

Supplement: Supplementary file 13 — Supplementary Data 11 [file 41467_2021_21636_MOESM13_ESM.zip › supplementary_prediction/cep_tp00607_KDPC_KDPB/cep_tp00607_KDPC_KDPB.png]

## KDPC\_ECOLI-KDPB\_ECOLI

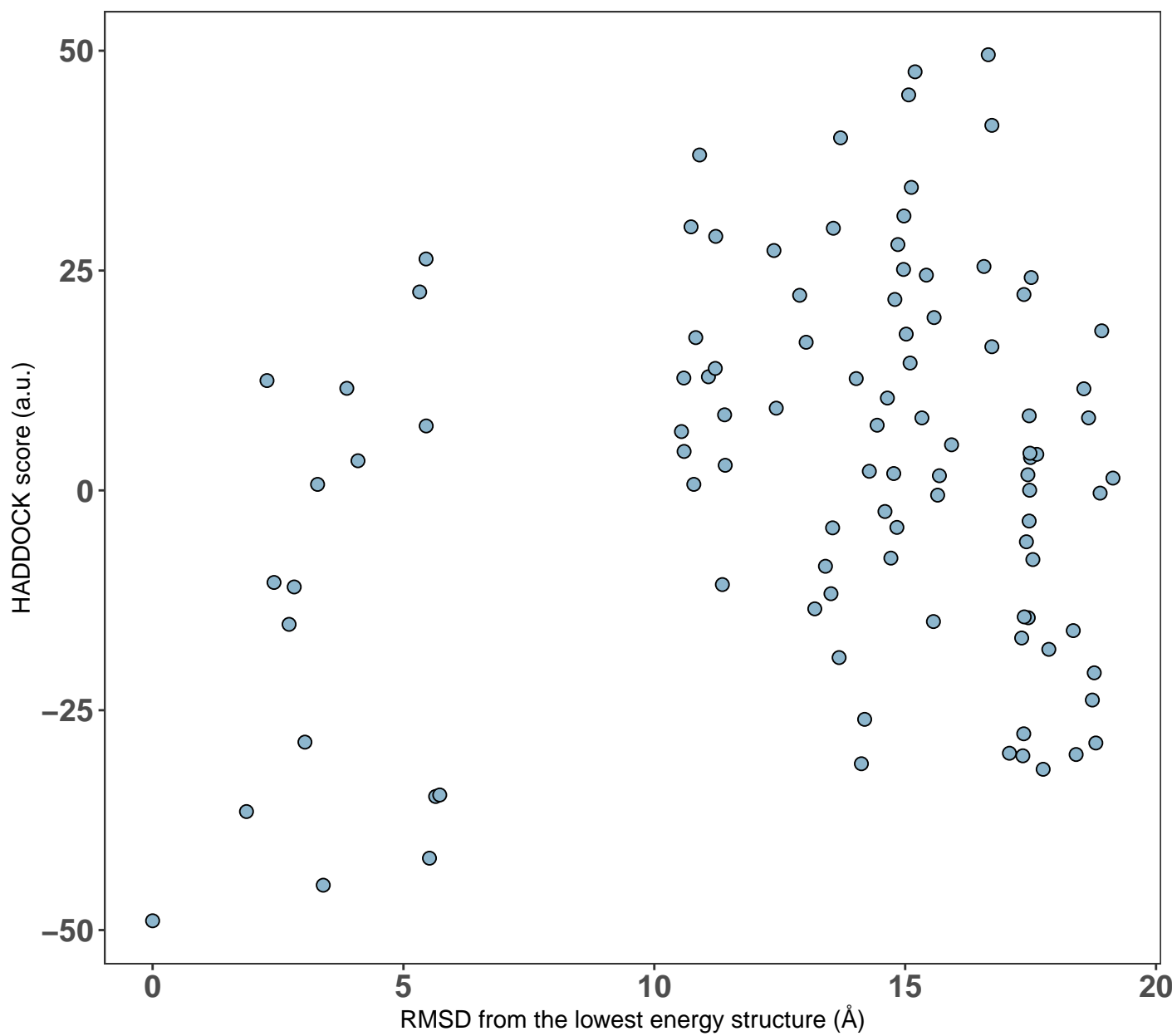

Supplement: Supplementary file 13 — Supplementary Data 11 [file 41467_2021_21636_MOESM13_ESM.zip › supplementary_prediction/cep_tp00607_KDPC_KDPB/cep_tp00607_KDPC_KDPB.pdf]

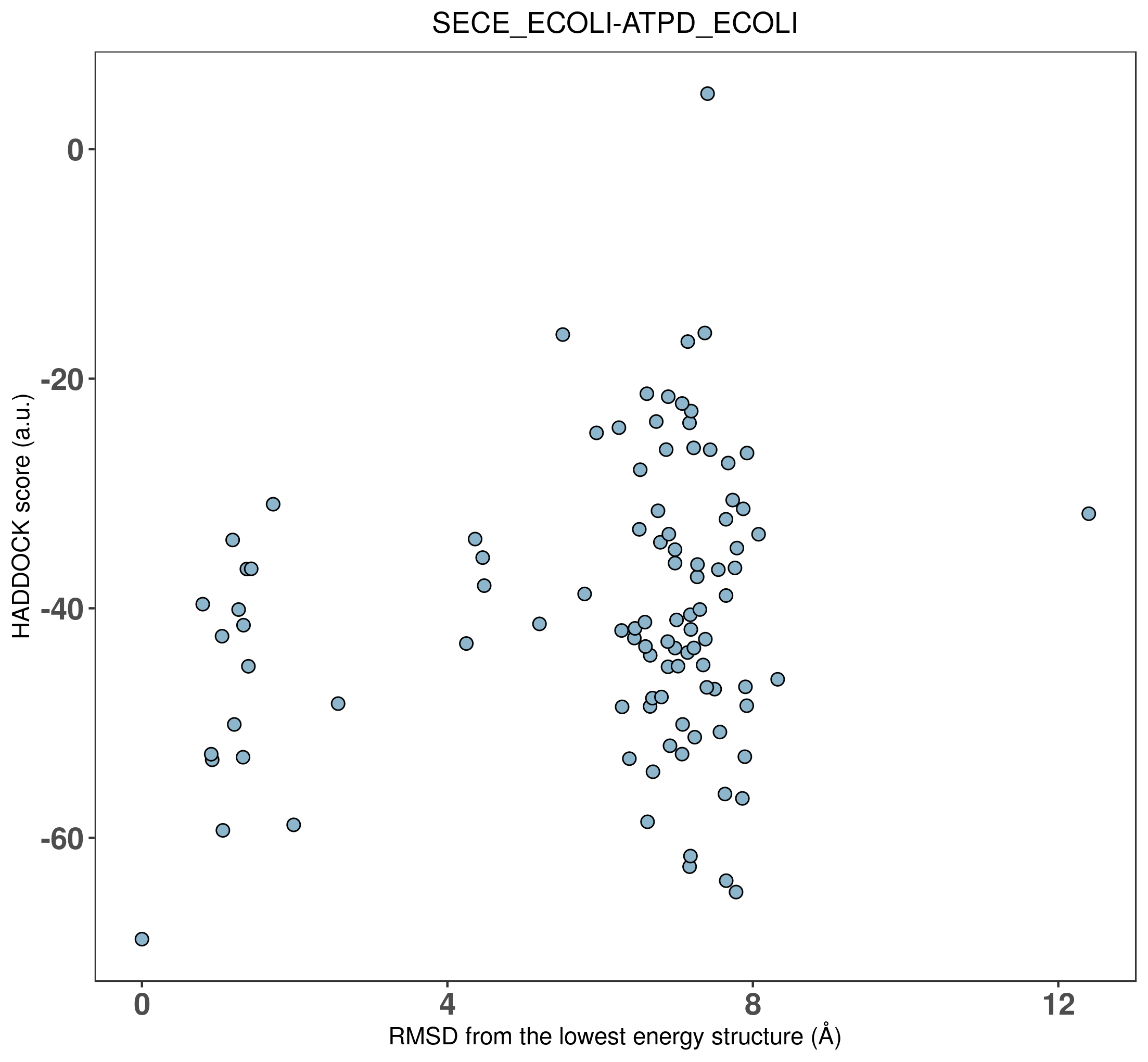

Supplement: Supplementary file 13 — Supplementary Data 11 [file 41467_2021_21636_MOESM13_ESM.zip › supplementary_prediction/membrane127199_SECE_ATPD/membrane127199_SECE_ATPD.png]

## SECE\_ECOLI-ATPD\_ECOLI

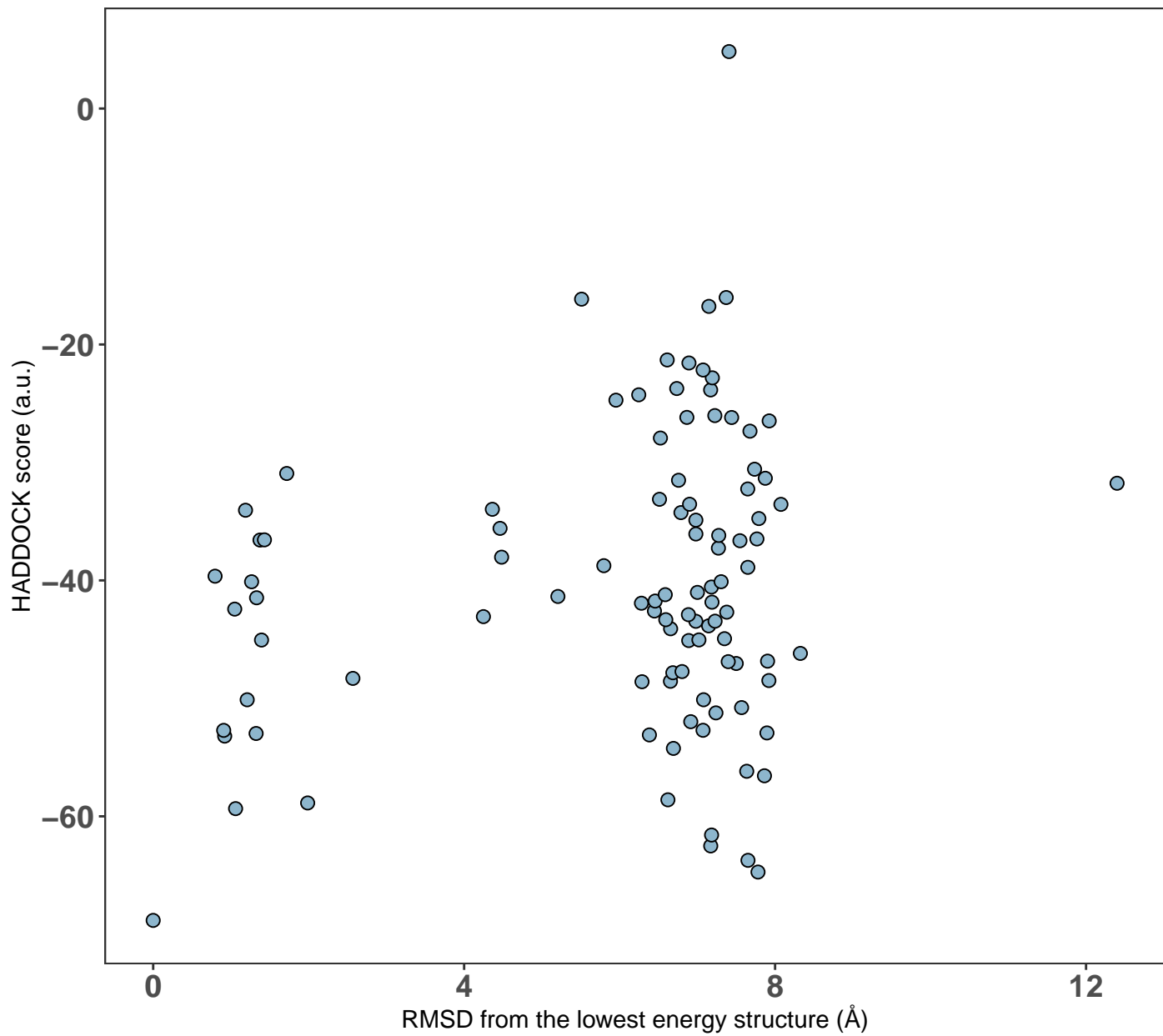

Supplement: Supplementary file 13 — Supplementary Data 11 [file 41467_2021_21636_MOESM13_ESM.zip › supplementary_prediction/membrane127199_SECE_ATPD/membrane127199_SECE_ATPD.pdf]

## SECE\_ECOLI-ATPD\_ECOLI

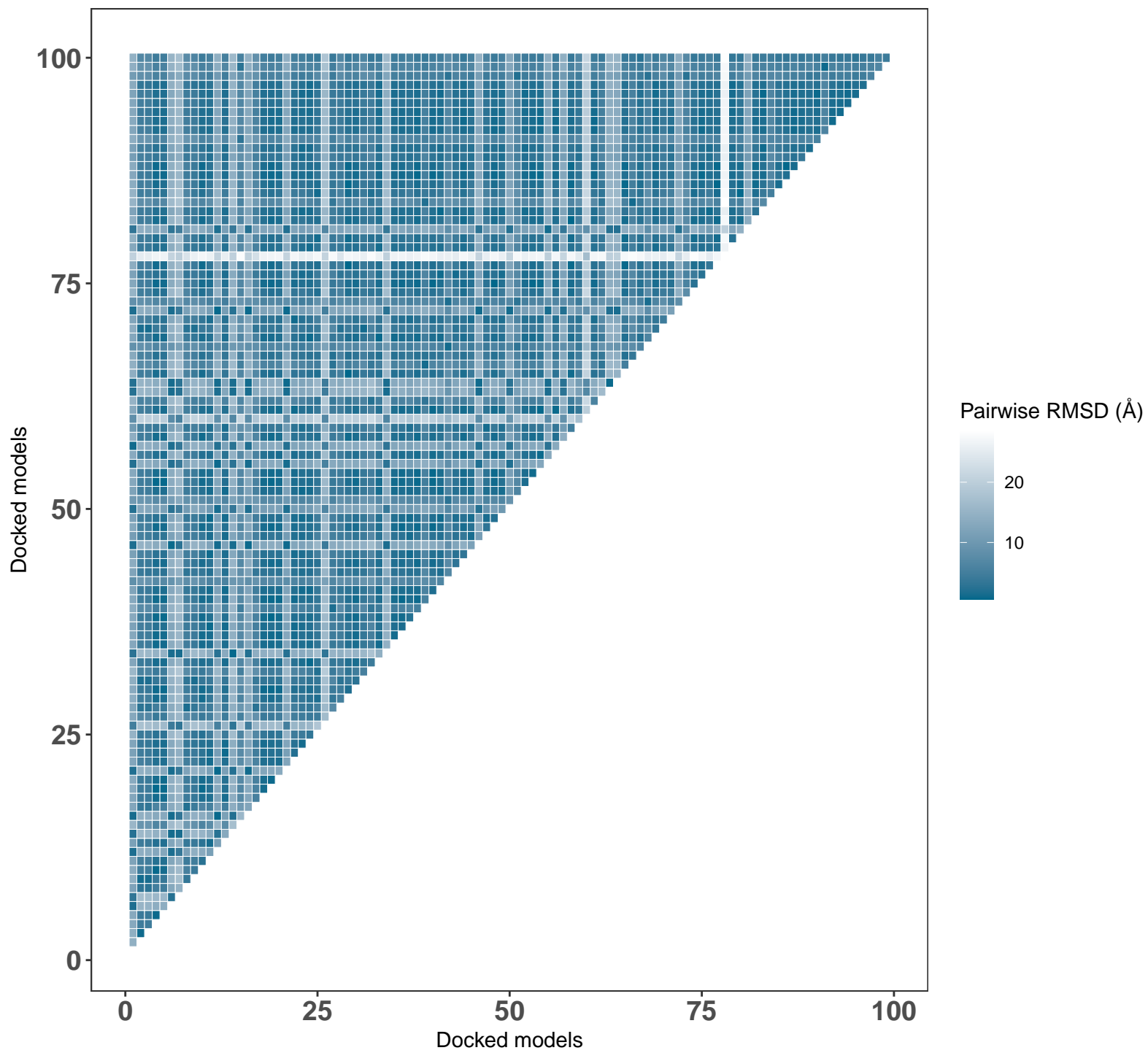

Supplement: Supplementary file 13 — Supplementary Data 11 [file 41467_2021_21636_MOESM13_ESM.zip › supplementary_prediction/membrane127199_SECE_ATPD/pwrmsd_membrane127199_SECE_ATPD.pdf]

## LNT\_ECOLI-GSPH\_ECOLI

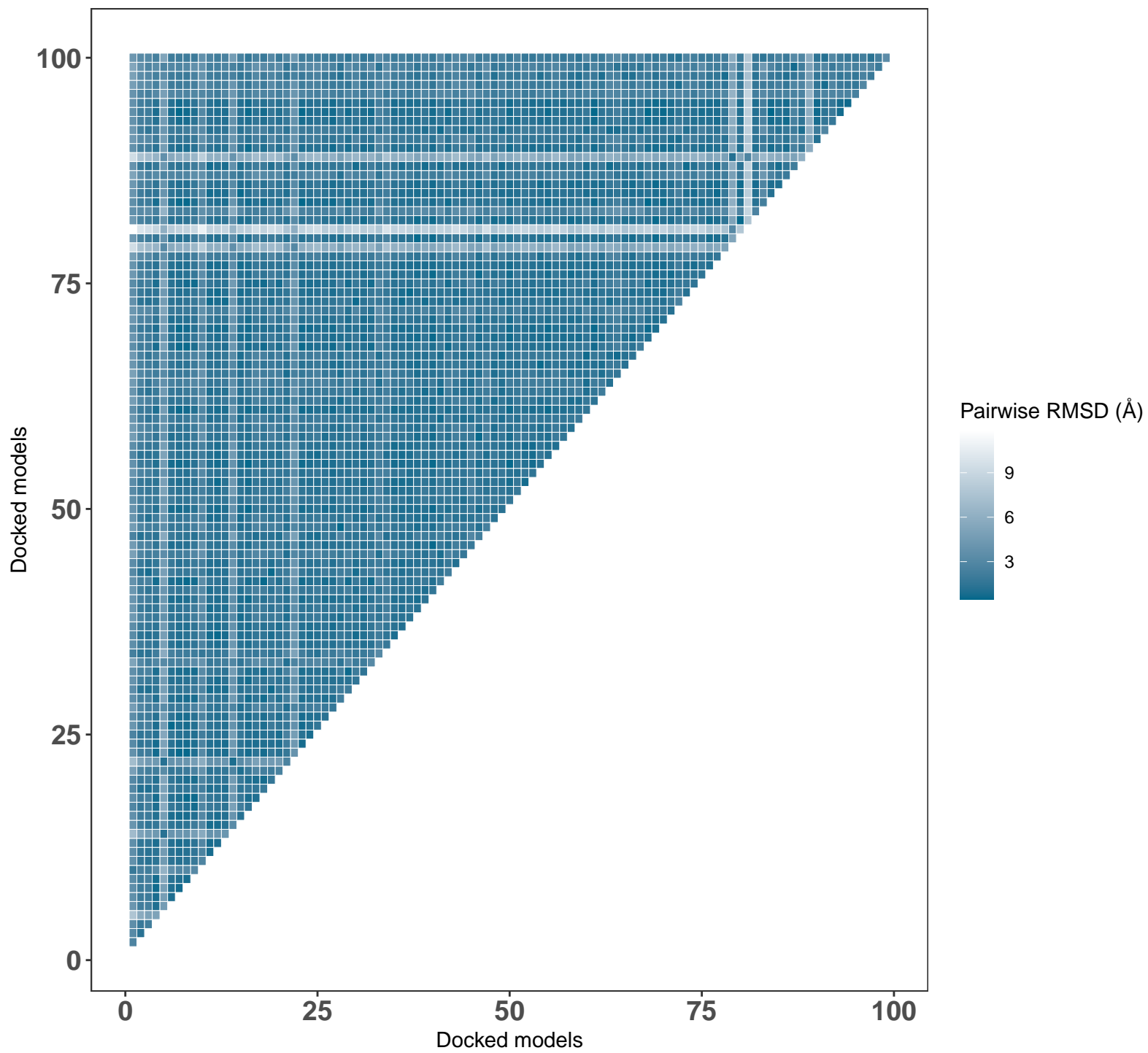

Supplement: Supplementary file 13 — Supplementary Data 11 [file 41467_2021_21636_MOESM13_ESM.zip › supplementary_prediction/membrane153326_LNT_GSPH/pwrmsd_membrane153326_LNT_GSPH.pdf]

## LNT\_ECOLI-GSPH\_ECOLI

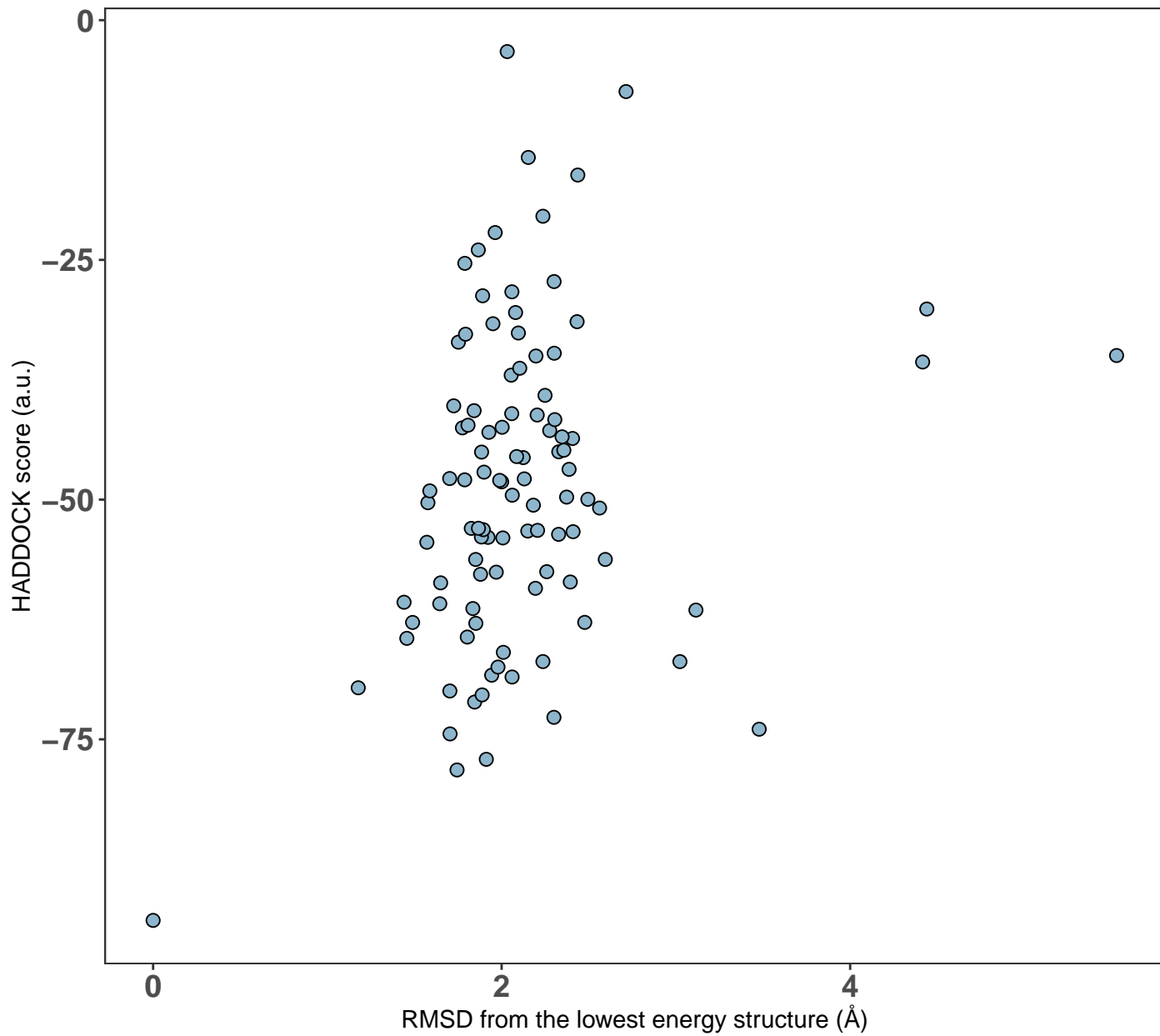

Supplement: Supplementary file 13 — Supplementary Data 11 [file 41467_2021_21636_MOESM13_ESM.zip › supplementary_prediction/membrane153326_LNT_GSPH/membrane153326_LNT_GSPH.pdf]

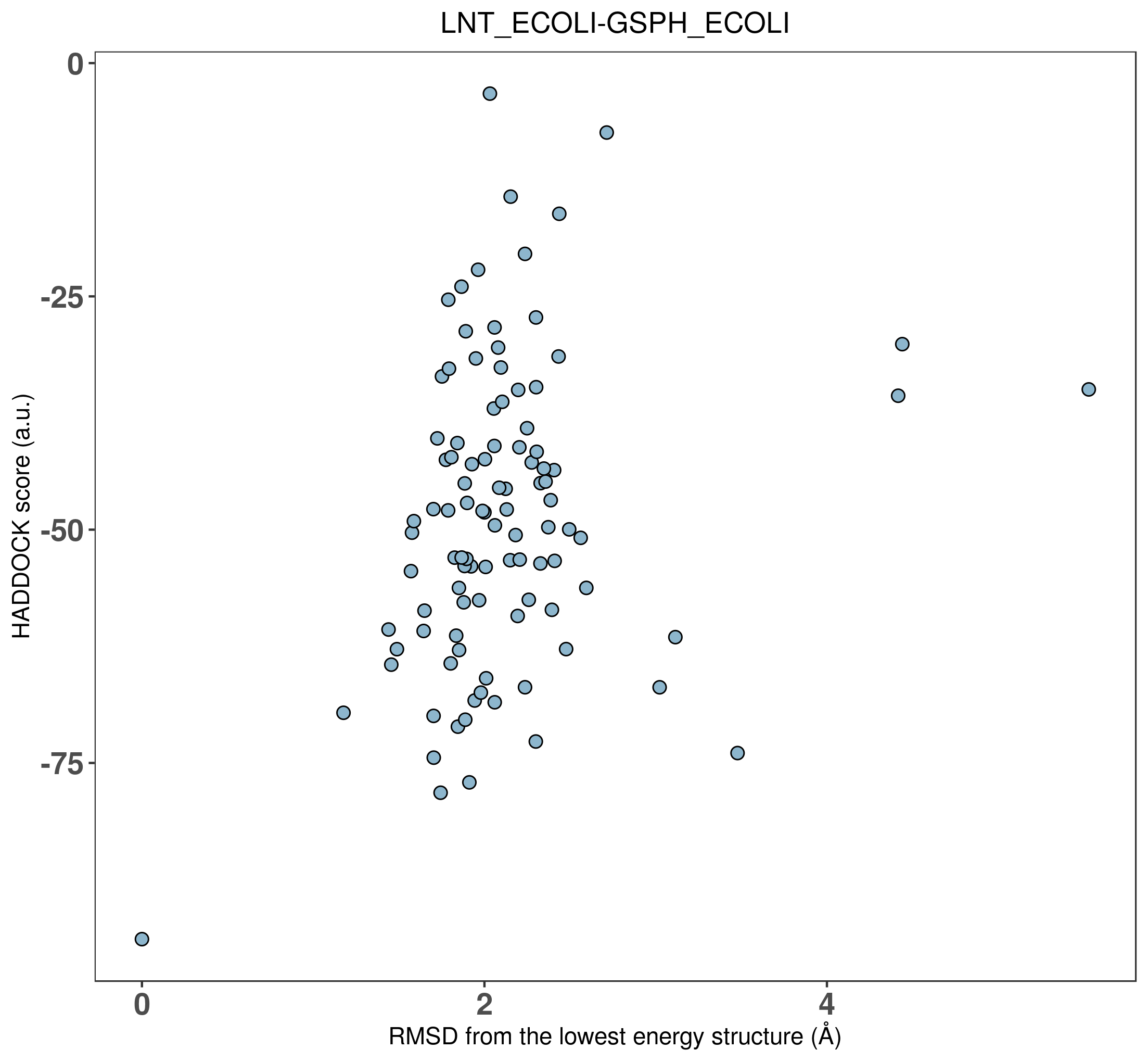

Supplement: Supplementary file 13 — Supplementary Data 11 [file 41467_2021_21636_MOESM13_ESM.zip › supplementary_prediction/membrane153326_LNT_GSPH/membrane153326_LNT_GSPH.png]

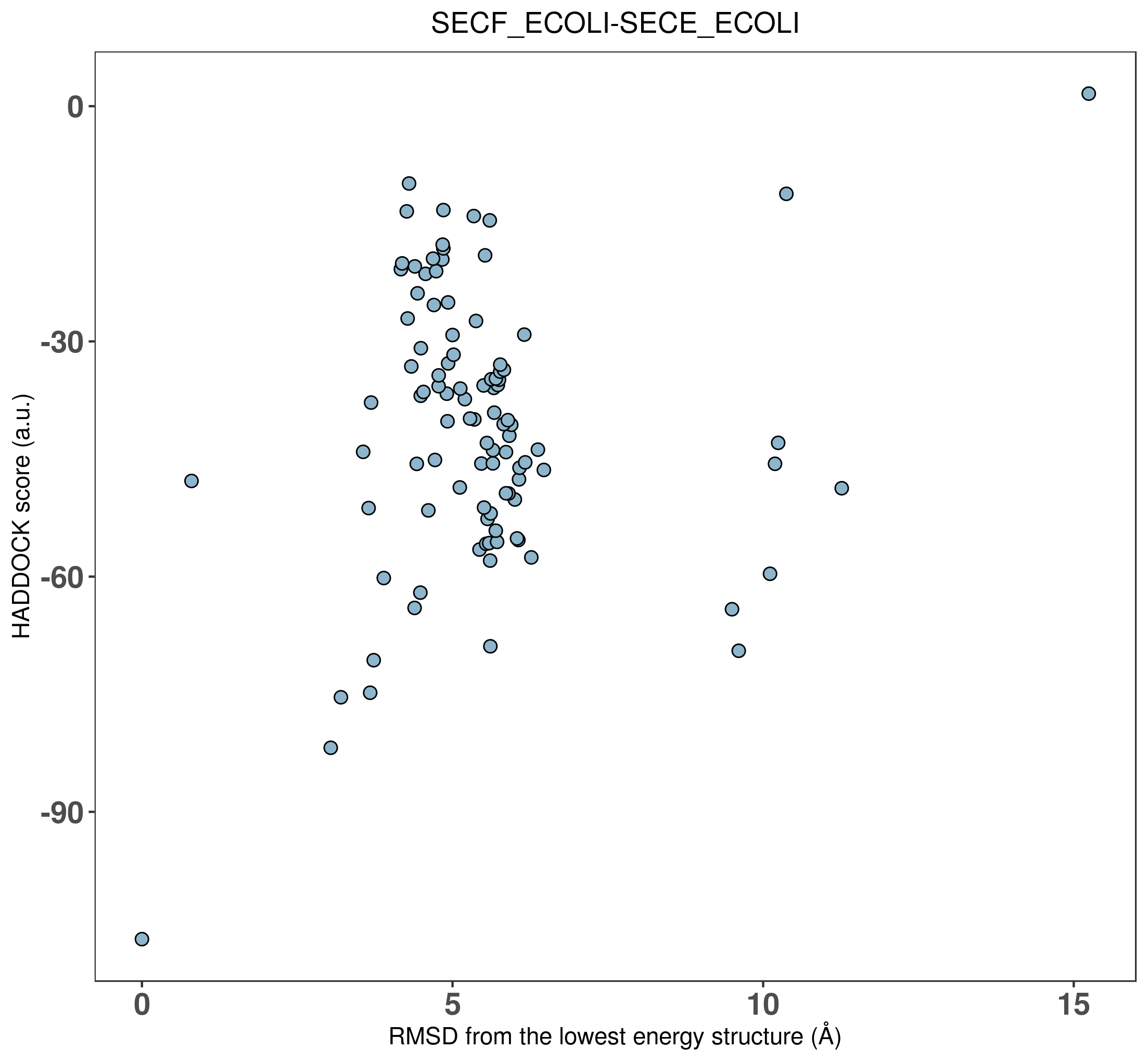

Supplement: Supplementary file 13 — Supplementary Data 11 [file 41467_2021_21636_MOESM13_ESM.zip › supplementary_prediction/cep_tp00809_SECF_SECE/cep_tp00809_SECF_SECE.png]

## SECF\_ECOLI-SECE\_ECOLI

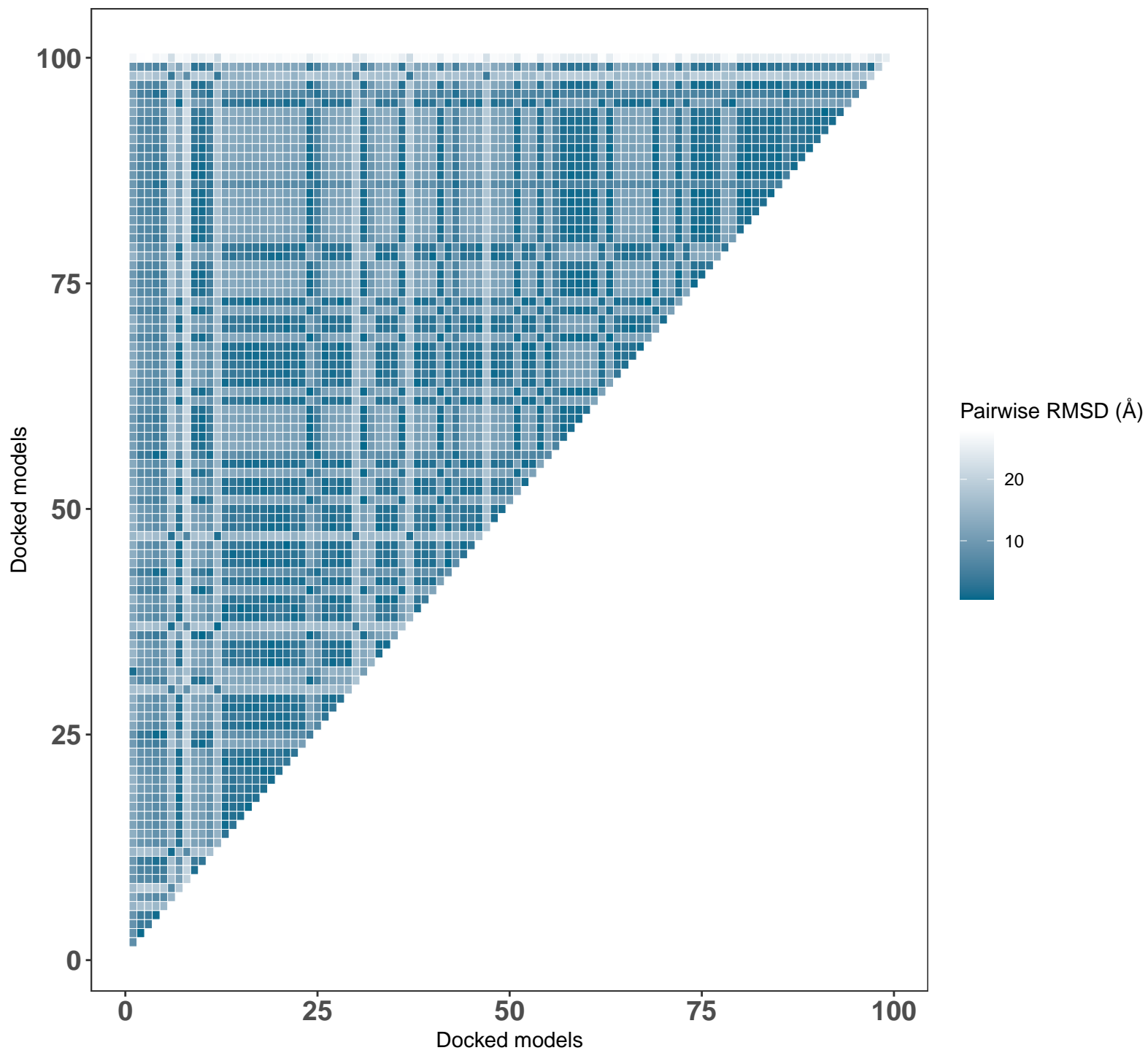

Supplement: Supplementary file 13 — Supplementary Data 11 [file 41467_2021_21636_MOESM13_ESM.zip › supplementary_prediction/cep_tp00809_SECF_SECE/pwrmsd_cep_tp00809_SECF_SECE.pdf]

## SECF\_ECOLI-SECE\_ECOLI

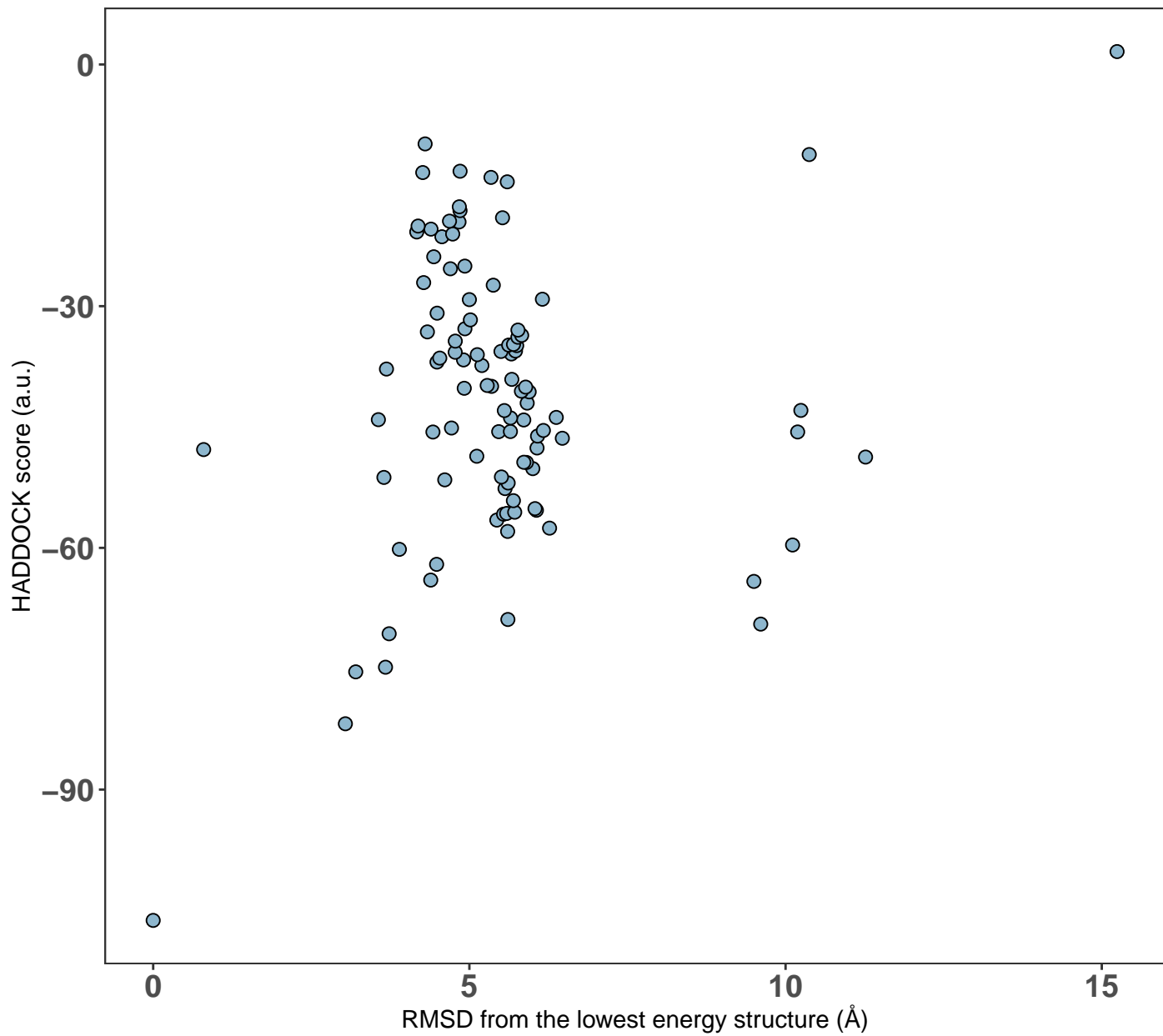

Supplement: Supplementary file 13 — Supplementary Data 11 [file 41467_2021_21636_MOESM13_ESM.zip › supplementary_prediction/cep_tp00809_SECF_SECE/cep_tp00809_SECF_SECE.pdf]

## SECG\_ECOLI-LPTF\_ECOLI

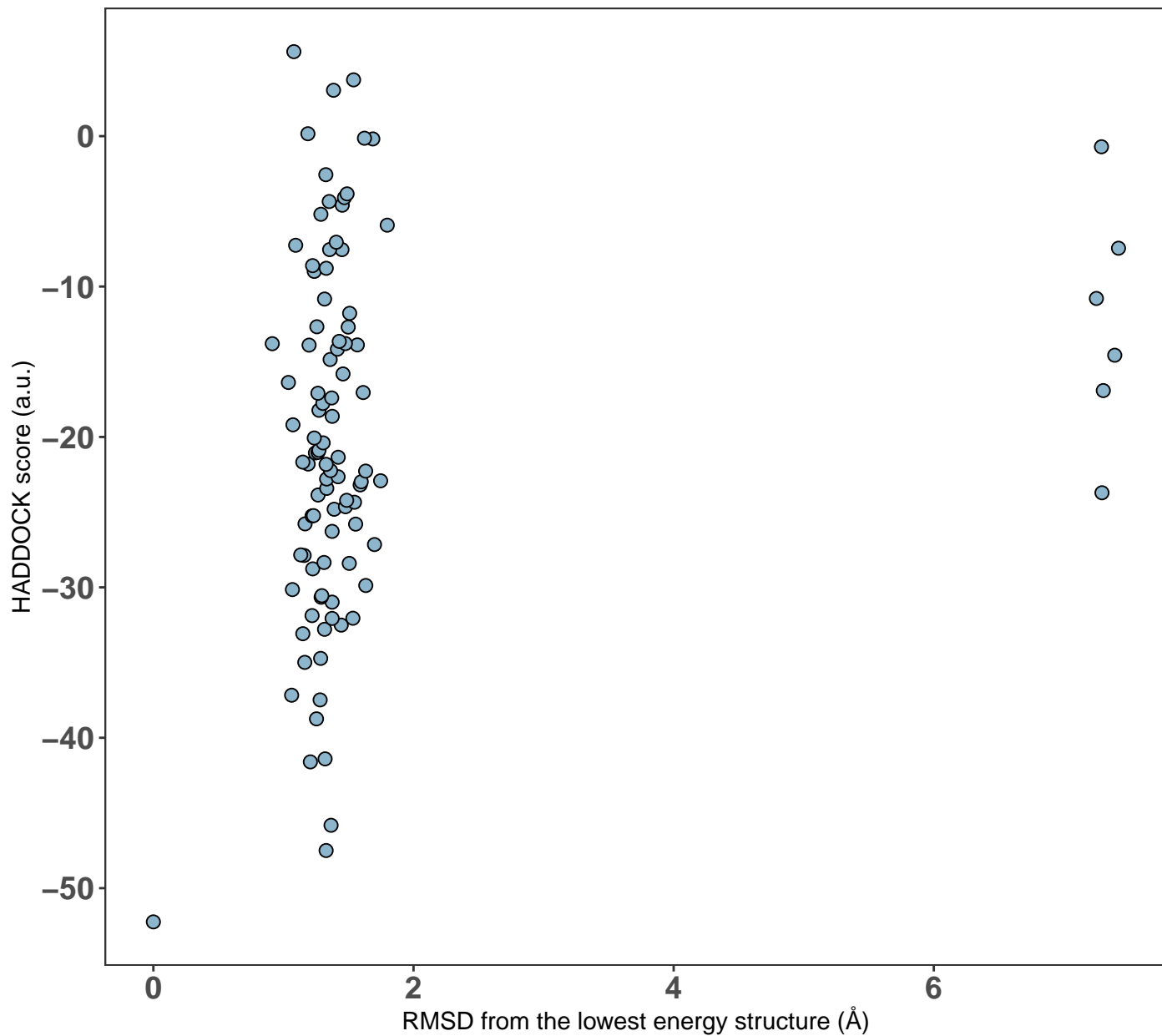

Supplement: Supplementary file 13 — Supplementary Data 11 [file 41467_2021_21636_MOESM13_ESM.zip › supplementary_prediction/membrane128329_SECG_LPTF/membrane128329_SECG_LPTF.pdf]

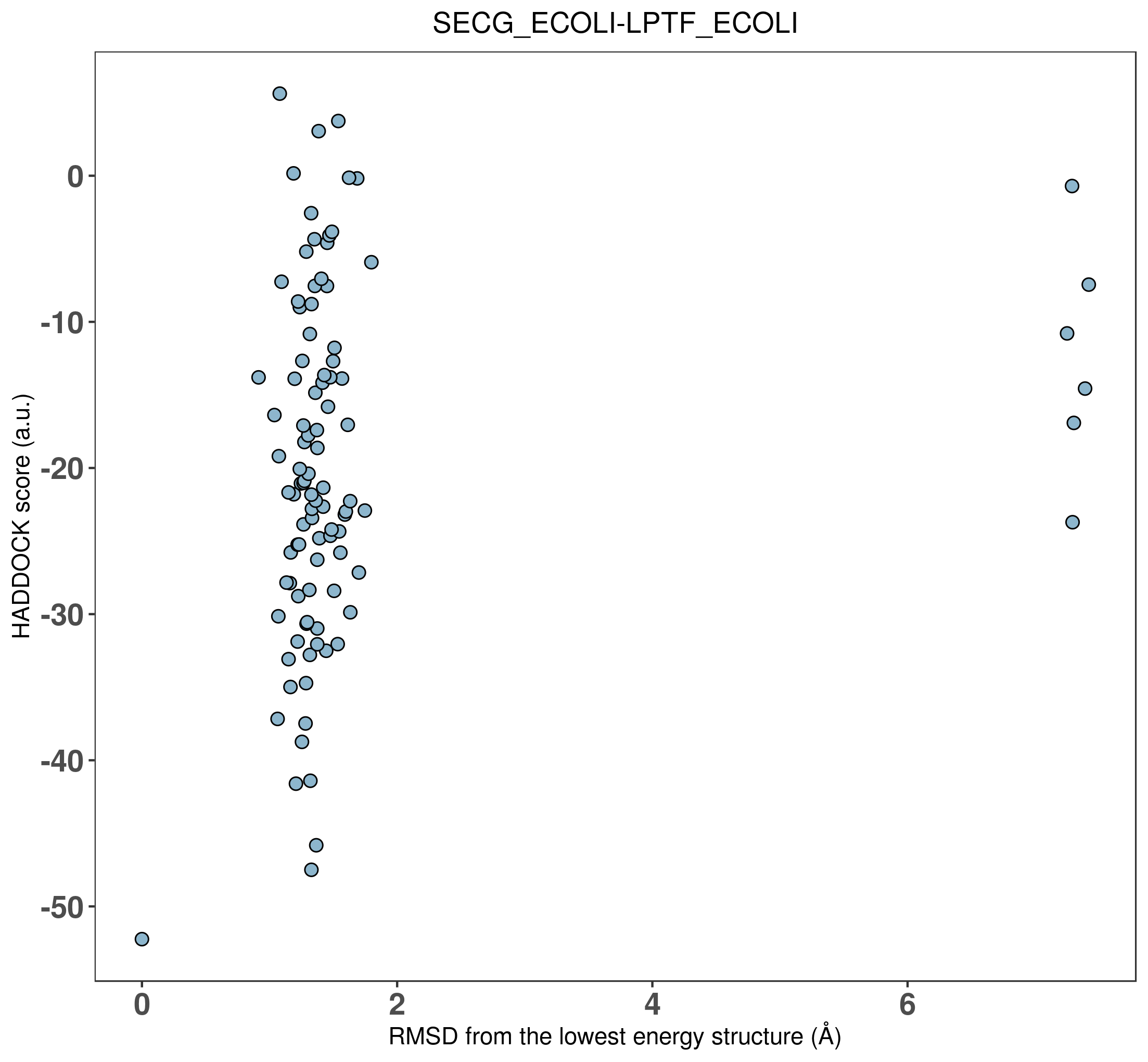

Supplement: Supplementary file 13 — Supplementary Data 11 [file 41467_2021_21636_MOESM13_ESM.zip › supplementary_prediction/membrane128329_SECG_LPTF/membrane128329_SECG_LPTF.png]

# SECG\_ECOLI-LPTF\_ECOLI

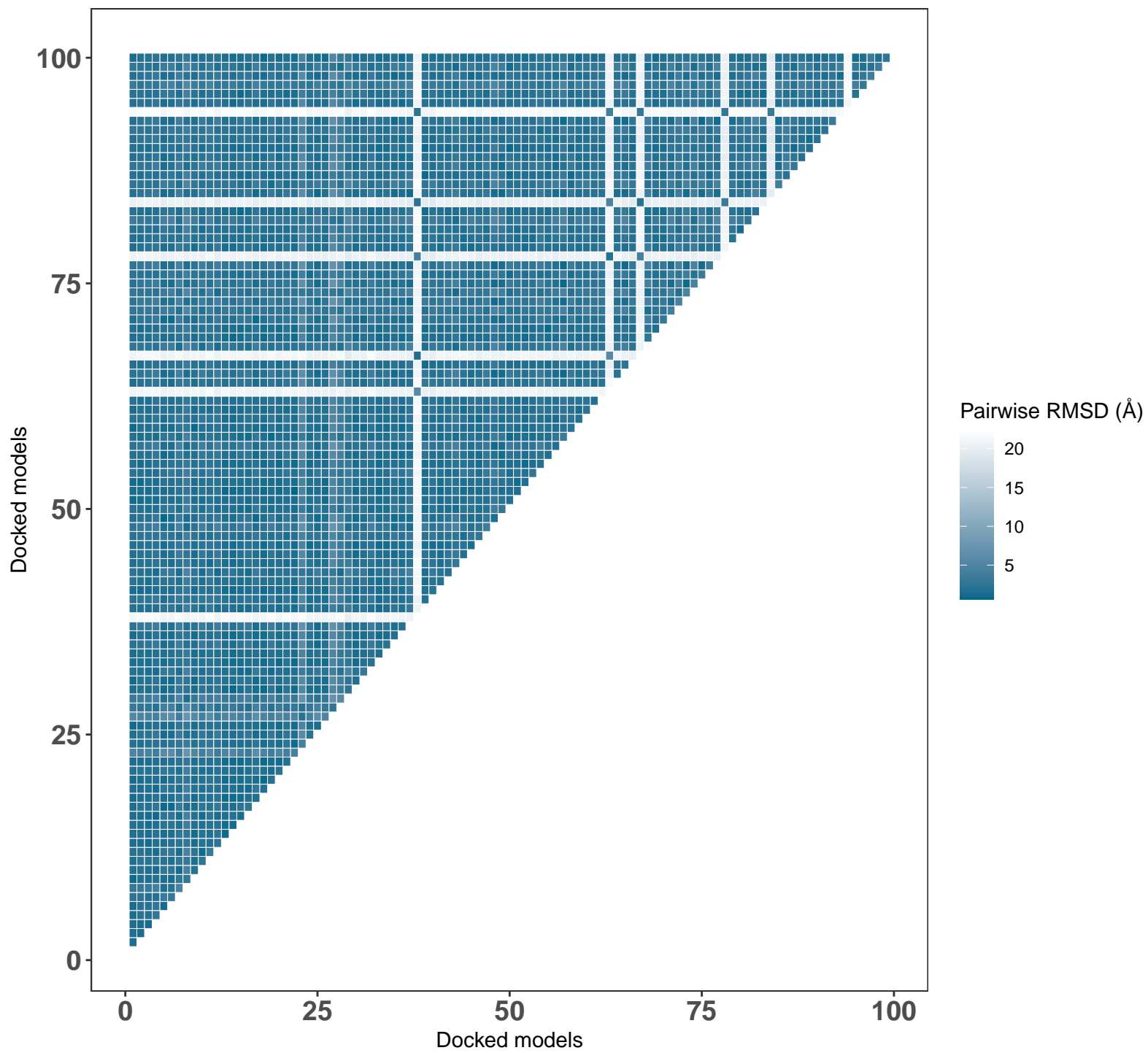

Supplement: Supplementary file 13 — Supplementary Data 11 [file 41467_2021_21636_MOESM13_ESM.zip › supplementary_prediction/membrane128329_SECG_LPTF/pwrmsd_membrane128329_SECG_LPTF.pdf]

## SUFC\_ECOLI-SUFB\_ECOLI

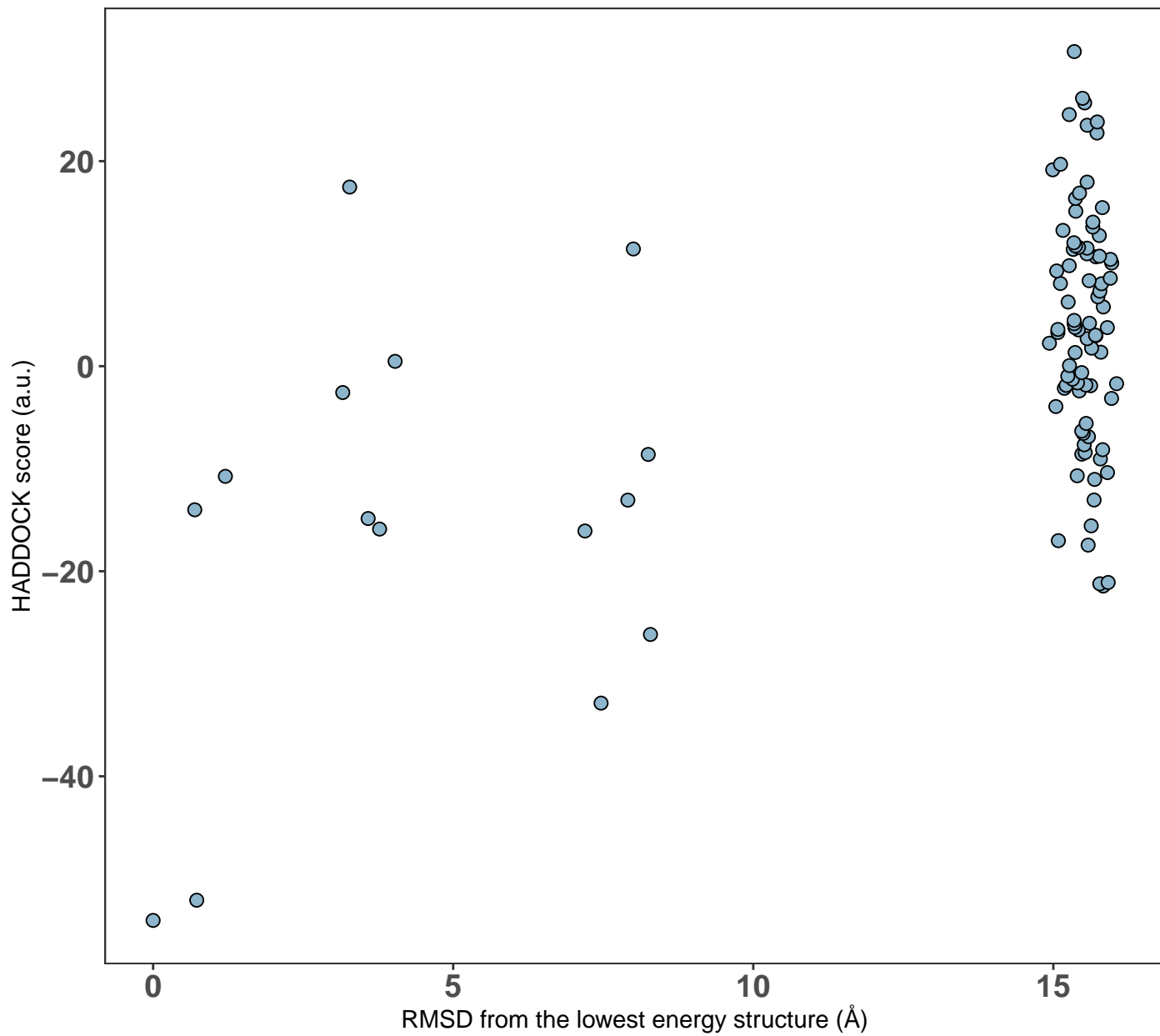

Supplement: Supplementary file 13 — Supplementary Data 11 [file 41467_2021_21636_MOESM13_ESM.zip › supplementary_prediction/cep_exp06715_SUFC_SUFB/cep_exp06715_SUFC_SUFB.pdf]

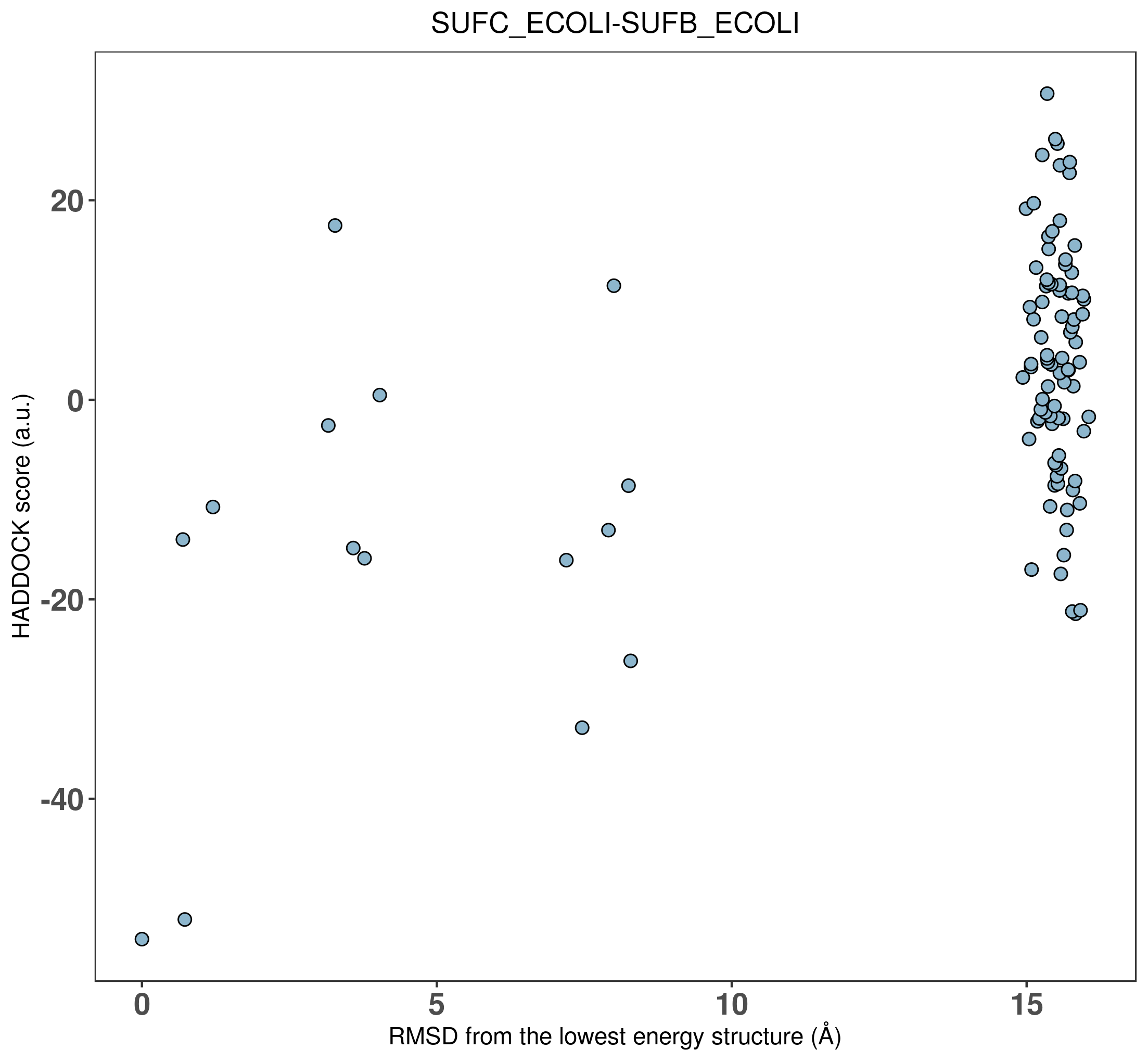

Supplement: Supplementary file 13 — Supplementary Data 11 [file 41467_2021_21636_MOESM13_ESM.zip › supplementary_prediction/cep_exp06715_SUFC_SUFB/cep_exp06715_SUFC_SUFB.png]

# SUFC\_ECOLI-SUFB\_ECOLI

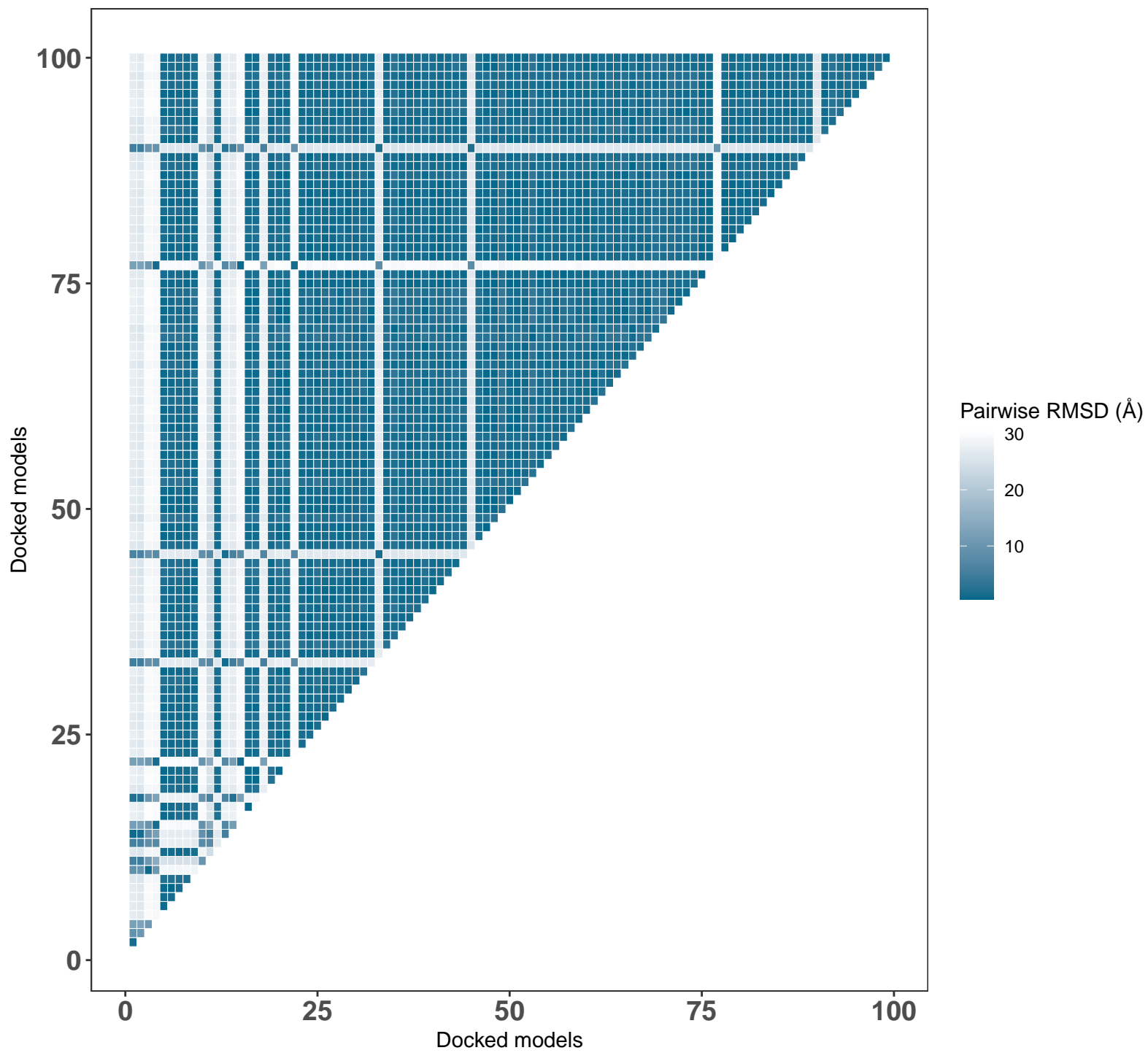

Supplement: Supplementary file 13 — Supplementary Data 11 [file 41467_2021_21636_MOESM13_ESM.zip › supplementary_prediction/cep_exp06715_SUFC_SUFB/pwrmsd_cep_exp06715_SUFC_SUFB.pdf]

## UUP\_ECOLI-MURJ\_ECOLI

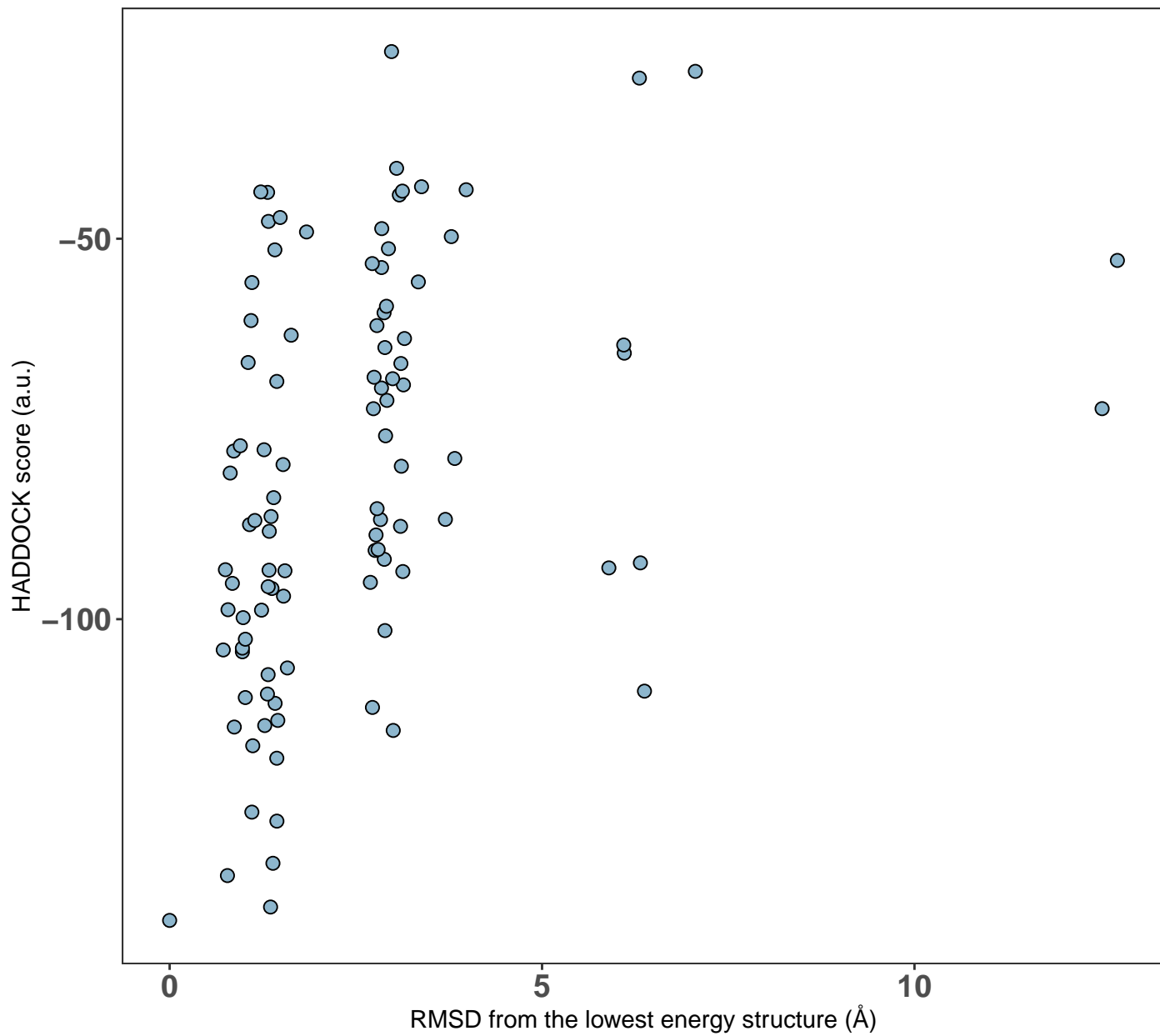

Supplement: Supplementary file 13 — Supplementary Data 11 [file 41467_2021_21636_MOESM13_ESM.zip › supplementary_prediction/addtl_membrane013341_UUP_MURJ/addtl_membrane013341_UUP_MURJ.pdf]

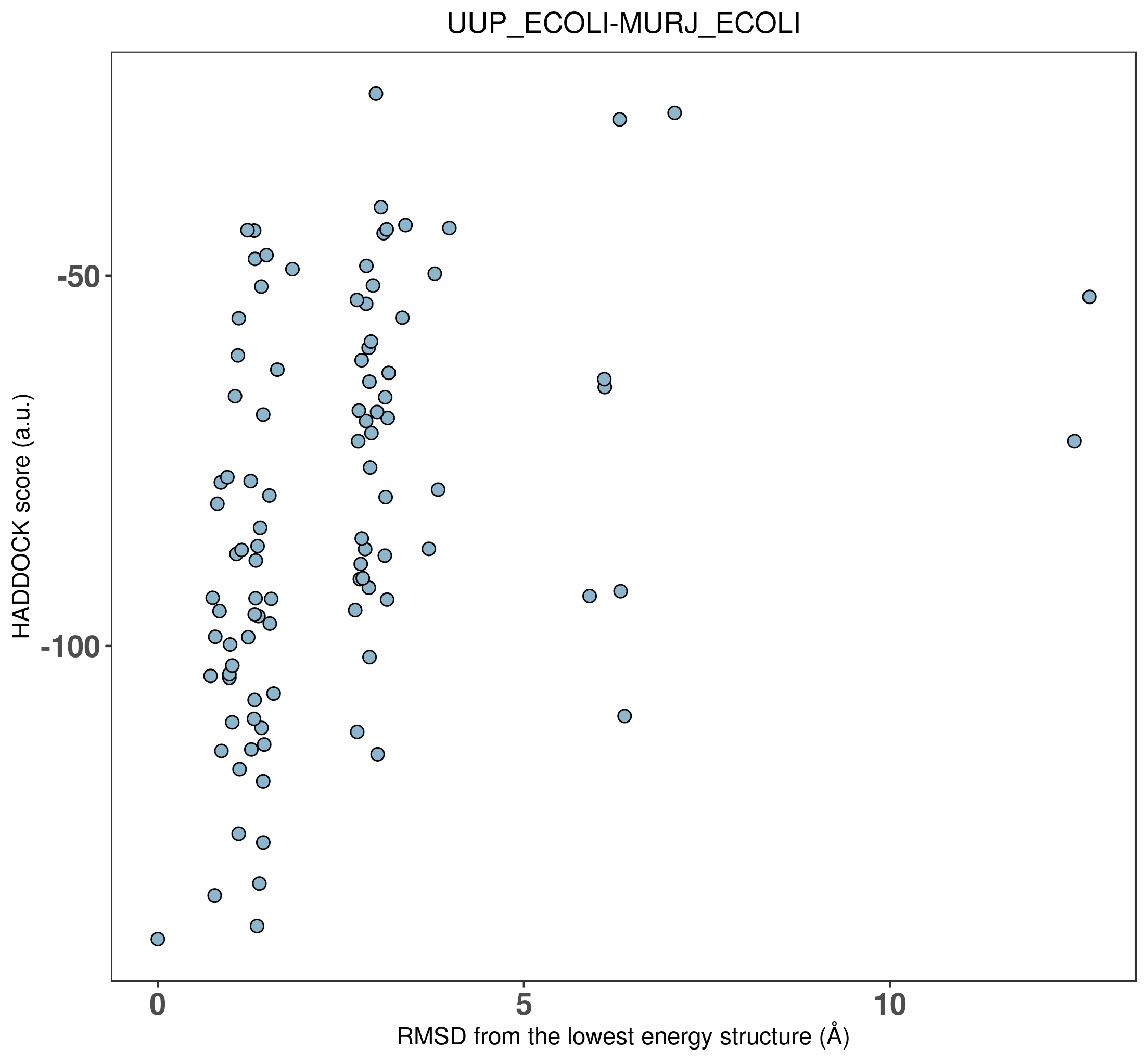

Supplement: Supplementary file 13 — Supplementary Data 11 [file 41467_2021_21636_MOESM13_ESM.zip › supplementary_prediction/addtl_membrane013341_UUP_MURJ/addtl_membrane013341_UUP_MURJ.png]

# UUP\_ECOLI-MURJ\_ECOLI

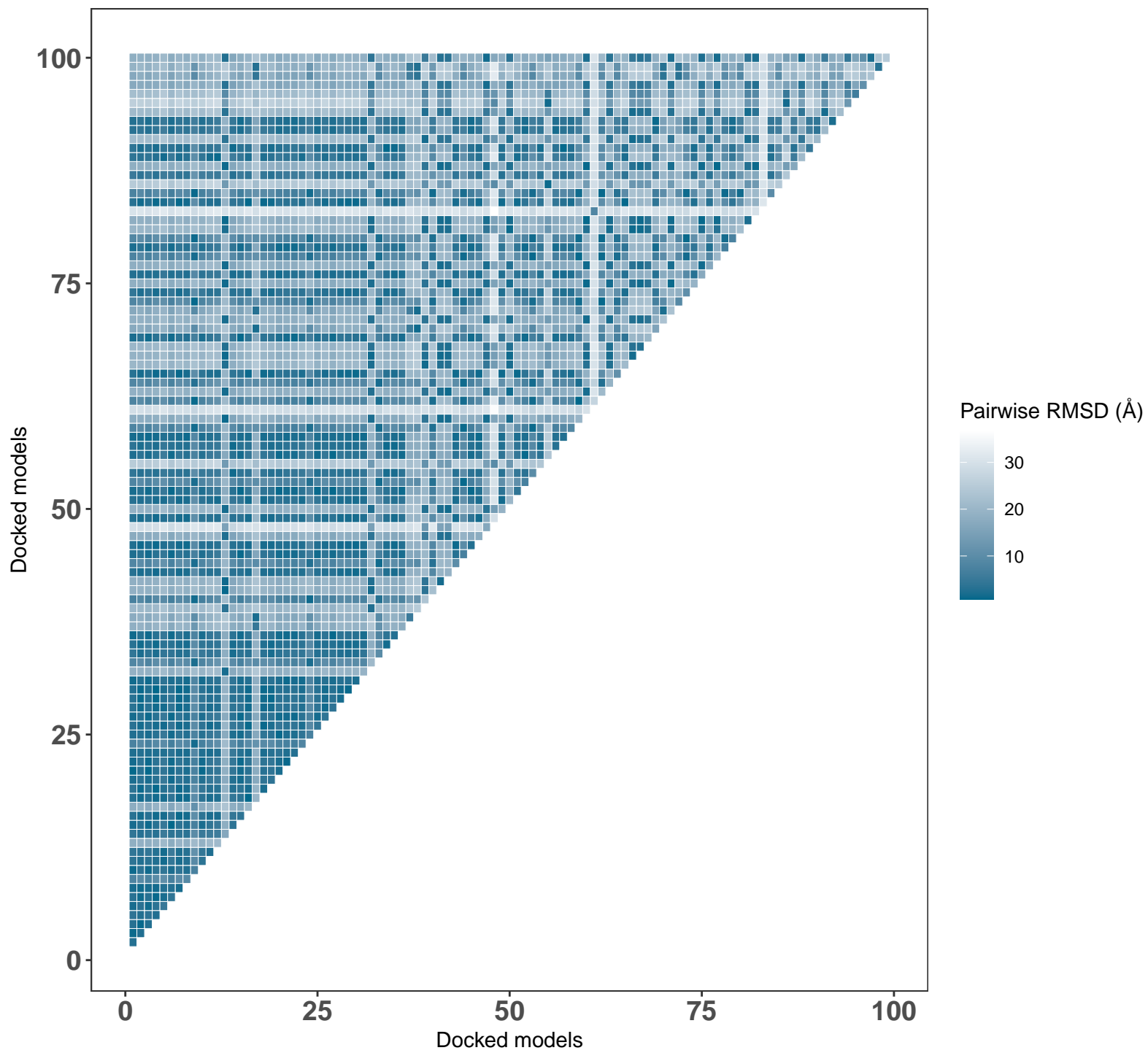

Supplement: Supplementary file 13 — Supplementary Data 11 [file 41467_2021_21636_MOESM13_ESM.zip › supplementary_prediction/addtl_membrane013341_UUP_MURJ/pwrmsd_addtl_membrane013341_UUP_MURJ.pdf]
