# Supplementary material for: Large-scale discovery of protein interactions at residue resolution using co-evolution calculated from genomic sequences
Source: Nat Commun. 2021 Mar 2;12:1396. doi: 10.1038/s41467-021-21636-z (PMC7925567; doi:10.1038/s41467-021-21636-z)
Supplement: Supplementary file 14 — Reporting Summary [file 41467_2021_21636_MOESM14_ESM.pdf]

nature research

Corresponding author(s): *Debora S. Marks*
Oliver Kohlbacher

Last updated by author(s): *2020-12-15*

Reporting Summary

Nature Research wishes to improve the reproducibility of the work that we publish. This form provides structure for consistency and transparency in reporting. For further information on Nature Research policies, see our Editorial Policies and the Editorial Policy Checklist.

Statistics

For all statistical analyses, confirm that the following items are present in the figure legend, table legend, main text, or Methods section.

n/a	Confirmed	
☐	☒	The exact sample size (*n*) for each experimental group/condition, given as a discrete number and unit of measurement
☒	☐	A statement on whether measurements were taken from distinct samples or whether the same sample was measured repeatedly
☒	☐	The statistical test(s) used AND whether they are one- or two-sided<br>*Only common tests should be described solely by name; describe more complex techniques in the Methods section.*
☒	☐	A description of all covariates tested
☒	☐	A description of any assumptions or corrections, such as tests of normality and adjustment for multiple comparisons
☒	☐	A full description of the statistical parameters including central tendency (e.g. means) or other basic estimates (e.g. regression coefficient) AND variation (e.g. standard deviation) or associated estimates of uncertainty (e.g. confidence intervals)
☒	☐	For null hypothesis testing, the test statistic (e.g. *F*, *t*, *r*) with confidence intervals, effect sizes, degrees of freedom and *P* value noted<br>*Give P values as exact values whenever suitable.*
☒	☐	For Bayesian analysis, information on the choice of priors and Markov chain Monte Carlo settings
☒	☐	For hierarchical and complex designs, identification of the appropriate level for tests and full reporting of outcomes
☒	☐	Estimates of effect sizes (e.g. Cohen's *d*, Pearson's *r*), indicating how they were calculated

Our web collection on statistics for biologists contains articles on many of the points above.

Software and code

Policy information about availability of computer code

Data collection	Data was collected using jackhmmer version 3.1b2, hmmsearch 3.1b2, DSSP (CMBI version by M.L. Hekkelman/2010-10-21), HADDOCK v2.2, and a custom branch of the EVcouplings Python package, https://github.com/debbiemarkslab/EVcouplings/pull/225
Data analysis	Data was analyzed using Python 3.8.3, Scikitlearn 0.23.2, Numpy 1.18.5, Pandas 1.1.0, and the EVcouplings Python package, https://github.com/debbiemarkslab/EVcouplings/pull/225

For manuscripts utilizing custom algorithms or software that are central to the research but not yet described in published literature, software must be made available to editors and reviewers. We strongly encourage code deposition in a community repository (e.g. GitHub). See the Nature Research guidelines for submitting code & software for further information.

Data

Policy information about availability of data

All manuscripts must include a data availability statement. This statement should provide the following information, where applicable:

- Accession codes, unique identifiers, or web links for publicly available datasets
- A list of figures that have associated raw data
- A description of any restrictions on data availability

The sequences and sequence annotation data analyzed during this study are available from UniProt (download date: Apr 1, 2017). The sequence location data analyzed during this study are available from ENA (download date: Feb 2017). The sequence taxonomy data analyzed during this study are available from the NCBI taxonomy database (download date: May 2020). The 3D-Structural information data analyzed during this study are available from the Protein Data Bank and SIFTS (download date Feb 1, 2018). The identities of complexes in the benchmark and prediction datasets generated are available in the Supplementary Material, and their full results are available online at https://marks.hms.harvard.edu/ecolicomplex/. All other data is available from the corresponding authors on reasonable request.

Field-specific reporting

Please select the one below that is the best fit for your research. If you are not sure, read the appropriate sections before making your selection.

☒ Life sciences ☐ Behavioural & social sciences ☐ Ecological, evolutionary & environmental sciences

For a reference copy of the document with all sections, see nature.com/documents/nr-reporting-summary-flat.pdf

Life sciences study design

All studies must disclose on these points even when the disclosure is negative.

Sample size

Sample sizes were determined based on amount of publicly available data. No new data was collected for this study.

Data exclusions

The following exclusion criteria were applied to all experiments in the paper, including analysis of the positive and negative benchmark sets, analysis of the spliceosome and previous *E. coli* experimental data, and analysis of the membrane proteome: To avoid analyzing protein pairs where the two proteins are paralogous, we implemented three filters. (1)removed all pairs of proteins where the first protein contains a PFAM domain that is found in the second protein. This exclusion criteria was predetermined based on our objective of finding coevolution between paralogous proteins (2) remoed all pairs where our structure comparison protocol found hits to the same chain of the same PDB structure. This exclusion criteria was not predetermined, but implemented after the observation that hits to the same chain displayed pathologically high coevolution scores, indicating distant homology. (3) removed all pairs of protein that display high-scoring coevolution along a diagonal between the two proteins. We consider these contacts to be artifactual due to their very high scores relative to known interactions. This exclusion criteria was not predetermined, but implemented after the observation that these hits displayed pathologically high coevolution scores.

For analysis of the *E. coli* membrane proteome, we chose to exclude genes with a non-traditional character in the E. coli genome, because these can indicate pseudogenes. This exclusion criteria was predetermined.

Replication

The findings of this project are purely computational. Assessment of scoring was performed on a held-out test set to maximize reproducibility in future datasets. Replications of the training-test partitioning were not performed, in keeping with ML best practices.

Randomization

Data points were randomly partitioned into the training and test sets.

Blinding

Investigators were not blinded to group membership (interacting vs. non-interacting) as this would have rendered development and analysis of scoring functions impossible.

Reporting for specific materials, systems and methods

We require information from authors about some types of materials, experimental systems and methods used in many studies. Here, indicate whether each material, system or method listed is relevant to your study. If you are not sure if a list item applies to your research, read the appropriate section before selecting a response.

Materials & experimental systems

n/a	Involved in the study
☒	☐ Antibodies
☒	☐ Eukaryotic cell lines
☒	☐ Palaeontology and archaeology
☒	☐ Animals and other organisms
☒	☐ Human research participants
☒	☐ Clinical data
☒	☐ Dual use research of concern

Methods

n/a	Involved in the study
☒	☐ ChIP-seq
☒	☐ Flow cytometry
☒	☐ MRI-based neuroimaging
